# Learning from regulatory failure: How Ostrom’s restorative justice design principle helps naïve groups create wiser enforcement systems to overcome the tragedy of the commons

**DOI:** 10.1371/journal.pone.0307832

**Published:** 2024-08-23

**Authors:** Daniel A. DeCaro, Marci S. DeCaro, Marco A. Janssen, Allen Lee, Alanea Graci, Devin Flener

**Affiliations:** 1 Department of Psychological and Brain Sciences, University of Louisville, Louisville, KY, United States of America; 2 Department of Urban and Public Affairs, University of Louisville, Louisville, KY, United States of America; 3 School of Sustainability, Arizona State University, Tempe, AZ, United States of America; 4 School of Complex Adaptive Systems, Arizona State University, Tempe, AZ, United States of America; 5 Center for Behavior, Institutions, and the Environment, Arizona State University, Tempe, AZ, United States of America; Ovidius University of Constanta: Universitatea Ovidius din Constanta, ROMANIA

## Abstract

Rule enforcement is critical in democratic, self-governing societies. Many political disputes occur when citizens do not understand the fundamental rationales for enforcement (e.g., COVID-19 pandemic). We examined how naïve groups learn and develop wise enforcement systems. Based on theories from behavioral economics, political science, psychology, and education, we predicted that groups need to experience failure of an enforcement system, but be guided on restorative justice principles to collectively learn from this failure. Undergraduate students (*N* = 288) from a Midwestern U.S. metropolitan university self-governed a simulated common-pool resource with real financial payoffs. Groups began with one of three conditions designed to create different experiences with enforcement and regulatory failure: (a) no enforcement (no communication or peer sanctioning), (b) lax enforcement (communication with peer-sanctioning), or (c) regulatory abuse (peer sanctioning without communication). Half then received facilitated guidance on restorative justice principles (e.g., discuss whether/why to use sanctions). To examine cooperation, we measured how well participants maintained the resource. To examine group learning, we created a novel coding system, which tracked groups’ constitutional decisions about conservation agreements and enforcement, conceptual understanding, and the enforcement systems they created. The no-enforcement and lax-enforcement conditions quickly yielded moderate cooperation via voluntary agreements. However, such agreements prevented groups from discovering how and why to use enforcement (peer sanctioning) to improve performance. Initial exposure to regulatory failure had different effects depending on facilitation. Unfacilitated groups fixated on initial misconceptions, causing them to abandon or create less sophisticated enforcement systems, hindering cooperation. Facilitated groups learned from prior failure—discovering principles of wise enforcement (e.g., collective efficiency, self-restraint)—and created more sophisticated enforcement systems (e.g., coordinated sanctions) that improved cooperation. Guidance on restorative justice principles and experience with regulatory abuse may be necessary preconditions for naïve individuals to understand and develop wiser collective enforcement systems.

## Introduction

Rule enforcement is essential to public order. Failure to enforce vital public policies creates insecurity and decreases societal cooperation [1, 2]. However, enforcement requires the use of coercion and force, “instruments of evil,” to ensure compliance [3, 4]. This reality creates a fundamental dilemma for modern democracies, which seek to enforce public order while promoting public autonomy and societal self-governance [5–7]. Democratic enforcement must be used wisely. Punitive surveillance and punishment systems that are ill-conceived, poorly justified, misused, or abused undermine societal cooperation and regulatory compliance by eroding legitimacy and crowding-out internal motivations [8–10].

Political disputes and public resistance to regulation have ballooned in recent decades [e.g., COVID regulations, 11, 12], along with scientific debate [13]. For many, it is unclear how to enforce rules without creating more conflict and undermining democratic freedoms and cooperation. According Vincent and Elinor Ostrom, Nobelist in political science and economics, these disputes are fundamentally constitutional disputes over foundational social contracts and regulatory systems [4, 7, 14]. Members of society must decide when, why, and how to use enforcement systems to encourage cooperation. This kind of collective dilemma cannot be adequately resolved if the stakeholders do not understand the basic rationales of effective enforcement [8, 15, 16]. Prior research has not examined how naïve groups learn from prior regulatory failure to update their understanding of enforcement systems.

We therefore employed a simulated common-pool resource experiment to examine how groups of university students—naïve citizens who typically lack enforcement experience—learn to effectively use and understand enforcement systems. We did so by modifying Elinor Ostrom’s preferred experimental paradigm, which is to create a financially important social-ecological dilemma in a laboratory setting [17, 18]. In particular, we varied groups’ initial experiences with enforcement, simulating specific forms of regulatory failure (no enforcement, lax enforcement, and regulatory abuse). We also used one of Ostrom’s “design principles,” restorative justice [19], to provide some groups with guidance about best practices for maintaining their enforcement strategies via open, democratic discussion about enforcement goals and rationales. This is the first laboratory experiment to confirm that groups benefit from prior regulatory failure and abuse if guided by Ostrom’s design principle for restorative justice.

We developed several novel research methods and analytical procedures to examine learning and the evolution of enforcement systems as a function of group decision-making. First, we developed a novel coding technique to enable researchers to analyze group communication, in order to measure and track how naïve individuals’ conceptual understanding of enforcement changes over time. This approach enables researchers to analyze group decisions that drive institutional evolution in cooperative dilemmas. Second, we integrate this approach with STEM education science. Doing so allows us to inform behavioral theory of collective action as well as education and learning theory. Specifically, we describe how group learning depends on both prior failure and conceptual guidance. We are the first to demonstrate that Elinor Ostrom’s design principles for self-governance can be used as an educational scaffold in a laboratory setting to help naïve individuals learn better ways to self-govern. Finally, most studies of enforcement systems place individuals in a passive role to accept or reject exogenously created governance systems. Such studies do not allow the individuals to create their own enforcement systems. We therefore attempt to advance scientific inquiry by reorienting researchers to examine the active role citizen learners play in creating and maintaining their own governance systems. Though imperfect, the current study attempts to advance scientific understanding and methodology by taking first steps. Specifically, our study approach encourages researchers to return to Vincent and Elinor Ostrom’s most important research practice—examining constitutional decision-making processes to understand how naïve individuals and groups create effective governance systems, such as enforcement [14, 20, 21].

In the following sections, we review prior research on enforcement and provide rationales for our experimental manipulations. Afterward, we describe the current study, hypotheses, and research methods. We conclude with implications, lessons learned, and future directions.

## Literature review

### Behavioral foundations of enforcement

Experiments examining the provision and effects of enforcement systems have proliferated as social scientists try to understand the behavioral foundations of enforcement. Some early experiments by Nobel laureate Elinor Ostrom examined provisioning of enforcement systems as a process of constitutional choice, with naïve groups creating peer-sanctioning systems by communicating and self-governing in common-pool resource dilemmas [e.g., 6, 18]. However, most experiments use constrained public good dilemmas, in which participants have limited agency to select from among a pre-determined set of enforcement options. Many of these experiments involve centralized enforcement, removing participants entirely from the direct role of governor or enforcer [22]. Prior research highlights the potential pitfalls of peer sanctioning (e.g., revenge-seeking, retaliatory punishment) and benefits of centralized, government-controlled enforcement systems (i.e., Hobbes’ “Leviathan”) to correct such regulatory abuse and prevent non-cooperation. Overall, prior research argues that the typical civic agent may be too naive and self-interested to rationally govern [7, 16, 23]. For instance, social science frequently claims that university students, though politically active and involved in various societal pursuits, may be particularly naïve or inexperienced as creators and maintainers of governance systems such as those that pertain to enforcement systems [24].

However, centralization does not overcome the fundamental problem, it merely shifts the focus of attention away from the root problems with enforcement [14, 25]. First, there is the *paradox of provision*. Case observation and laboratory experiments indicate that naïve civic agents, including university students and other types of citizens, resist ceding control to governments (“Leviathan”). They exhibit preferences to disobey or dismantle such regulatory systems, not create and comply with them [2, 11, 26, 27]. Similar observations have been made for city planning, and other sectors, in which enforcers and/or the public fail to enforce rules due to such factors as effort costs, lack of understanding, and low political will [15, 28–30]. Second, citizen participation (self-governance) and regulatory agency are inseparable in democratic societies [13, 19]. Democratic decision-making processes are used to create, implement, legitimize, and accept enforcement [12, 31–33]—as Hardin (1968) said, “mutual coercion, mutually agreed upon” [5]. These same naïve civic agents elect the public officials that design and implement regulatory systems. They serve as jurors, police officers, lawyers, and judges. They shape broad regulatory goals and procedures via public discourse. They judge the legitimacy of the enforcement systems governing them [14, 23, 32, 34]. Thus, even with centralization, citizens must understand enforcement systems at some point, whether by direct civil experience or via experience as students in classrooms in universities or elsewhere.

#### Conceptual understanding and acceptance

Observation and case study research indicates that citizens of all types must comprehend the rationales for enforcement, or they will lose the ability to hold central governments and their delegated enforcers accountable [14, 21]. When misconceptions are widespread, diverse citizens refuse to comply with reasonable rule enforcement and laud regulatory slippage as “freedom.” Such actors cannot constructively critique regulatory systems or discern which spheres of personal and societal activity are best left to altruism and voluntary cooperation. Prominent reviews of field studies and lab experiments indicate that individuals may reject and fail to comply with regulatory systems that are imposed on them without their full consent or participation in the design of those systems [8, 23, 29, 30].

For example, DeCaro and DeCaro [12] recently demonstrated that U.S. citizens (registered voters) in New York, California, Texas, and Florida who perceived federal and state COVID regulations as imposed enforcement systems failed to accept or comply with those regulations, compared to citizens who perceived those same regulations as democratically chosen [see also, 11]. DeCaro and colleagues [9, 10] have replicated this finding in common-pool resource laboratory experiments. Specifically, they found that naïve groups accept and comply better with enforcement systems (e.g., conservation agreements and financial fines) when they vote on the systems or directly create them via open communication and democratic (i.e., shared) constitutional decision-making. Groups that have conflicting voting outcomes or do not communicate openly and make group decision democratically tended to achieve poor compliance and cooperation, as individuals rejected the enforcement systems. In short, it appears that individuals may lose the civic understanding and will to maintain effective enforcement systems that support widespread societal cooperation.

Elinor and Vincent Ostrom’s foundational case study research indicates that effective enforcement systems emerge from direct experience and conceptual insight gained by trial and error, open communication, and democratic deliberation [14, 21]. Such experience must spread to all types of citizens and permeate civic education [21, 33]. As dilemma stakeholders experiment with self-governance, they encounter regulatory failures and learn the advantages and disadvantages of different regulatory approaches. This experience informs rationales and methods to create wiser enforcement systems. In the most robustly cooperative systems documented in research, stakeholders use shared decision-making processes to decide rules and agreements and the means of enforcement [19].

### Restorative justice and legitimization

Stakeholders in these successful cooperative systems also typically adhere to principles of restorative justice [19, 23]. First, they hold multilateral hearings, giving enforcers and defectors opportunity to discuss why they behave in particular ways and raise any concerns they have about rule enforcement. Second, they use this information to reconcile conflict and maintain productive interpersonal relationships, via mutual understanding, forgiveness, and well-reasoned changes to existing rules and enforcement systems. Third, they pair sanctions with warnings, penalizing only if violations persist [cf. 35]. These mechanisms constitute a broader system of democratic enforcement and graduated sanctions. This process is thought to promote cooperation by legitimizing enforcement, ensuring that everyone understands and internalizes social contracts, and helping to reduce animosity and, therefore, vengeful sanctions [for interdisciplinary reviews, see 23, 31, 32, 36].

For example, in the previously mentioned laboratory experiments [9, 10] and COVID regulation field study [12] by DeCaro and colleagues, it was found that perceived legitimization of enforcement by democratic decision-making and restorative justice were strongly associated with psychological acceptance, internalization (i.e., intrinsic motivations), compliance, and cooperation. Similar findings have been reported in the criminal justice system, problem-solving courts, and policing [34, 37]. Theses finding have been replicated in field studies examining compliance with regulatory governance of common-pool resources in a variety of domains (e.g., coral reef and forest management) [38, 39].

#### Civic education and experiential learning

In theory, these principles can be taught. However, field evidence suggests that “design principles” fail because groups do not understand or appreciate the wisdom behind the principles or how to apply them, unless discovered via direct experience [40, 41]. This observation mirrors recent studies of discovery-based, exploratory learning in STEM education. For example, research indicates that when trying to learn complex systems or concepts, students who attempt to solve novel physics or mathematics problems before being taught key principles learn the concepts better than students being taught with more traditional lecture-then-practice methods [42–46]. This idea of reversing the order of lesson and practical experience may appear counterintuitive. However, research suggests that the trial-and-error process of exploratory learning highlights the need to make sense of the situation. Learners become aware of what they do, and do not know, raising awareness of knowledge gaps. This process motivates learners to explore and test the problem space, discerning important features [47]. In short, individuals begin to better understand what solutions work and do not work, and why. Importantly, this insight appears to be limited to the problem dimensions they explored firsthand [38].

Bush, DeCaro, and DeCaro [38] applied these education principles to learning in social-ecological dilemmas. Participants played a game that simulated a dilemma involving deforestation and water degradation by uncontrolled cattle farming. At the beginning of the experiment, the researchers educated all participants about the basic ecological dimensions of the dilemma (e.g., effects of deforestation, ecological thresholds). Afterward, half of the groups played the game once (Game 1), before being taught a formal lesson about the social dimensions of the dilemma (e.g., interdependence, rivalry). They then played the game a second time (Game 2). The remaining groups completed these tasks in reverse order: social dimensions lesson, Game 1, then Game 2. If the most important aspect of learning from experience is getting key concepts as early and directly as possible before practice, then the groups that received the formal lesson first should learn key concepts better. However, if experience facilitates conceptual understanding and motivation to learn, then playing the game first should better prepare participants to learn from future direct instruction. The researchers found support for the latter hypothesis. All groups understood the taught ecological dimensions equally poorly, despite having been directly taught them from the beginning. However, participants that played the game first learned the social dimensions well, and better than those who were taught these same principles upfront.

These studies have important implications for our understanding of learning and collective action in societal dilemmas, such as those that pertain to enforcement systems to govern common-pool resources. Individuals may be predisposed to learn from discovery-based exploratory learning, enabling them to learn from prior failure better than prior instruction. Such learning, and opportunity for failure, may be essential to the evolution of wiser enforcement systems in society. We now discuss the current research study and the core hypotheses that emerge from this prior literature.

## Current study

### Benefits of experiencing regulatory failure

This background literature suggests that groups may learn how to create and use wiser enforcement systems if they are allowed to experiment with and explore unwise enforcement firsthand, but no experiments have directly tested this idea. Thus, our first research question was: Are conceptual understanding and cooperation highest when groups experience abuse of enforcement? Individuals may need to experience the full pitfalls of regulatory failure to understand how and why to address them in institutional reforms.

### Conceptual benefits of facilitated guidance

STEM education on exploratory learning further indicates that guidance (e.g., lessons, guiding principles) is needed after exploration to solidify insights gained from experience [42, 43, 48]. We suggest that Elinor Ostrom’s “design principles” may be used as guidance to fulfill this educational requirement [49]. Therefore, our second research question was: Does prompting groups to consider design principles of restorative justice after initial regulatory failure help them recognize and constructively discuss critical features during communication, facilitating learning of wise enforcement principles and improving cooperation [cf. 19, 50, 51]?

### Experimental design and hypotheses

To test these ideas, we conducted a common-pool resource experiment with university students using a simulated foraging task [18]. Simulations are often used to examine basic learning, reasoning, decision-making, and cooperative processes in societal and social-ecological dilemmas [17, 18, 52]. Though unique in some ways, university students have frequently been used in Elinor Ostrom and others’ lab experiments to study behavior of naïve individuals [17, 18]. These simulations mimic fundamental ecological and social dynamics of real dilemmas by creating resource scarcity and interdependency, in which individuals’ actions affect others’ actions and outcomes. In the typical resource dilemma, poor coordination exacerbates competition for scarce resources, causing conflict and resulting in overconsumption of the resource. The resource system eventually collapses if the dilemma stakeholders fail to devise governance systems (e.g., conservation agreements, enforcement systems) to curtail competition [5].

We used treatments designed to emulate different initial regulatory environments thought to impact the types of regulatory failure groups experience. We also provided some groups with guidance in the form of Ostrom’s design principles for restorative justice. To examine potential effects of learning order, we varied the timing of key elements, including capacity for communication, peer-sanctioning, and facilitation (i.e., guidance).

We assessed cooperation in terms of resource conservation and sustainability. Groups that cooperate better sustain the resource, ensuring that group members cultivate and collect more resources [18]. We also created a novel coding system to code the group communication, examining their constitutional decisions about conservation agreements and rule enforcement, their conceptualization of enforcement, and the specific types of enforcement systems they created. Finally, we used surveys of key social-psychological variables (e.g., goals of enforcement, perceived procedural justice and self-determination, rule internalization and acceptance, and trust) to better understand group behavior.

All treatments are shown in Table 1, using three letters to signify the treatment given during each of three experiment phases: *N* (no communication or penalties), *C* (unfacilitated communication with penalties), *F* (facilitated communication with penalties), and *P* (penalties with no communication). Overall, we hypothesized that groups given facilitated communication would cooperate most following these treatments, compared to groups with unfacilitated communication. We predicted that this facilitation benefit would be found when comparing the facilitated and unfacilitated versions of each treatment (e.g., PCN vs. PFN). Second, we hypothesized that groups with the greatest degree of initial regulatory failure (i.e., use of penalties combined with lack of cooperation) would benefit most from facilitation. Our specific predictions and rationales for each treatment pairing are explained next.

**Table 1:** Experimental design.

Treatment	Phase 1	Phase 2	Phase 3	Description	Failure Type
	Rd 1–3	Rd 4–6	Rd 7–9		
CNN (*n* = 11)	**Comm (Pen)**	**N/A**	N/A	Communication followed by no communication	Lax
FNN (*n* = 11)	**Facil (Pen)**	**N/A**	N/A	Facilitated communication followed by no communication	Lax
NCN (*n* = 11)	N/A	**Comm (Pen)**	**N/A**	No communication followed by communication and no communication	Absent
NFN (*n* = 14)	N/A	**Facil (Pen)**	**N/A**	No communication followed by facilitated communication and no communication	Absent
PCN (*n* = 13)	Pen	**Comm (Pen)**	**N/A**	Penalties with no communication followed by communication and no communication	Abuse
PFN (*n* = 12)	Pen	**Facil (Pen)**	**N/A**	Penalties with no communication followed by facilitated communication and no communication	Abuse

Treatment labels are given three letters signifying treatment during each of the 3 experiment phases (Phases 1–3). N (no communication or penalties). C (communication with penalties). F (facilitated communication with penalties). P (penalties with no communication). For example, the CNN treatment is *communication followed by no communication*. N/A (all treatments removed). Bolded entries indicate diagnostic phases, during communication (Phase A) and immediately after communication (Phase B).

During Phase 1, groups in the CNN condition (Treatment 1) began with communication and the ability to penalize group members, followed by two phases (Phases 2, 3) without communication or penalties (Table 1). This condition was designed to emulate regulatory failure in the form of lax or weak (i.e., underutilized) enforcement. When groups begin with communication and peer sanctioning, they lack firsthand experience with enforcement. Because of this lack of experience, prior research suggests that it is unlikely that groups will choose to use enforcement, and instead attempt to rely on voluntary cooperative agreements [18].

Ceasing communication and sanctioning during Phases 2 and 3 allows us to observe post-treatment effects—namely, whether individuals continue to cooperate (i.e., comply with their group’s conservation agreement) when they are no longer able to communicate or enforce the agreement. This design enables us to examine potential legitimization and crowding-out effects of prior enforcement on intrinsic motivation (i.e., internalization), shedding additional light on the groups’ enforcement systems [9, 10].

All other treatments were variants of CNN. Treatment 2 (FNN) was the same as CNN, except that it included facilitated communication (i.e. facilitated communication followed by no communication). Facilitation provides some conceptual guidance, potentially helping to highlight diagnostic features of enforcement systems via social learning. Greater awareness and discussion of these features should help groups make novel insights, improving their conceptual understanding, enforcement systems, and cooperation. We therefore predicted FNN to outperform its counterpart, CNN, by comparison.

Treatments 3–4 (NCN, NFN) mirrored CNN and FNN, except that they began Phase 1 without communication or peer sanctioning. Specifically, the NCN condition began without communication (or peer sanctioning), followed by communication (and peer sanctioning), and then no communication (or peer sanctioning). The NFN condition added facilitation during the middle phase (Phase 2). These two conditions were designed to emulate initial regulatory failure due to no enforcement. Our rationales for including these comparison conditions are as follows. As we previously noted, groups that begin with communication (e.g., CNN) often decide to voluntarily cooperate before they experience the social dilemma task [6, 10]. If reasonably successful in cooperating initially, CNN and FNN groups may never witness severe non-cooperation common to social dilemmas, which is the primary justification for enforcement. In contrast, the initial lack of communication and peer sanctioning should ensure that NCN and NFN groups experience such cooperative failure before reaching the communication phase of the experiment. Thus, when these groups communicate during Phase 2, they may be better informed by firsthand experience, promoting their learning. In other words, NCN should have greater firsthand experience with the key features of the dilemma (i.e., scarcity, interdependency, competition), enabling more productive communication and social learning. Facilitation (NFN) should enhance this learning, yielding even greater insight. However, to the extent that experience with peer sanctioning is key to learning the fundamental rationales and use of enforcement, these conditions are hypothesized to lead to less learning and cooperation than treatments that begin with sanctioning.

Treatments 5–6 (PCN, PFN) began Phase 1 with peer sanctioning but no communication. For example, in the PFN condition, participants began with peer sanctioning (without communication), followed by facilitated communication (and peer sanctioning), and then no communication (or peer sanctioning). These treatments were intended to initially create circumstances for poorly coordinated and unjustified enforcement (i.e., illegitimate penalties without group discussion) and higher potential regulatory abuse and conflict [8–10, 53]. This design approach may seem counterintuitive (i.e., intentionally setting groups up for extreme failure initially). However, if assumptions about exploratory learning are correct, then these treatments have the potential to yield the greatest social-learning and cooperative outcomes. If exploratory learning in a social dilemma is optimized by exposure to all essential behavioral challenges and institutional features [19, 54], then PCN and PFN should be superior to NCN and NFN.

However, facilitation may be required for groups to constructively navigate heightened conflict and misconceptions caused by initial, unjustified enforcement, in order to learn constructive lessons from that experience. Therefore, we hypothesized that PCN would backfire, undermining learning and performance. Prior conflict and misconceptions may persist and grow, creating a vicious cycle. In contrast, we hypothesized that PFN would lead to superior learning and cooperative outcomes overall, combining dual benefits of optimized exposure and facilitation. We expected that these groups would be enlightened with firsthand knowledge of (a) why enforcement is needed, and what does not work well and why, as well as (b) insights from restorative justice needed to discover base principles of legitimate enforcement. In short, PFN may be optimally positioned to learn from prior mistakes.

In summary, overall we hypothesized that facilitation (restorative justice principles) would benefit learning and cooperation, yielding deeper conceptual understanding of enforcement and more sophisticated enforcement systems. We also specifically hypothesized that cooperative performance would be especially enhanced by facilitation when groups began with regulatory abuse (i.e., PFN), rather than no regulation at all (i.e., NCN, NFN) or weak regulation (i.e., CNN, FNN). Finally, we expected performance to be worst with regulatory abuse when such facilitation was not provided (i.e., PCN).

## Materials and methods

### Participants

Participants (*N* = 288; age: *M* = 19.00 years, *SD* = 1.81; 61% female; 64% Caucasian, 17% African-American) were undergraduate students at a U.S. Midwest metropolitan university. Participants were recruited from introductory psychology courses and approximately 60 introductory courses across other disciplines in exchange for payment ($3 show-up fee, plus in-game earnings). Some participants (59%) also received credit in their psychology course for arriving to the session on time. We selected university students as participants because we are interested in the ability of naïve individuals to learn from experience. This information is relevant for our understanding of civic and STEM education. Though it poses some limitations for generalization [24], the sampling of undergraduates for a common-pool resource experiment is consistent with many prior studies [e.g., 54], including Nobel laureate Elinor Ostrom’s research on societal self-governance and enforcement systems [e.g., 6, 17, 18].

### Procedure

The experiment was conducted in a networked computer lab with private cubicles. All materials and instructions were presented to participants on the computer and verbally by the experimenter. The sessions were run with 8 to 12 individuals at a time. When participants arrived at the lab, we first administered informed consent. Participants were prohibited from communicating with each other upon arrival. After consent, participants read introductory instructions, which explained the focal decision task (i.e., basic instructions for the resource dilemma) and payment. To ensure participants understood the task instructions and how payment would be determined, we asked them to complete a monetized quiz. Participants earned $0.10 for each correct answer (maximum of $0.50) and received immediate feedback on each question. The experimenter then answered any questions [cf. 10, 18]. In order to familiarize participants with the basic task environment and computer controls, participants completed a 4-minute practice round on a private screen without other players. After practice, the experimenter answered any remaining questions. The computer then randomly assigned the participants to four-person groups. Group members were identified by an assigned Player Number 1–4. Participants were prohibited from divulging their names or personal information. Immediately after the end of each group’s treatment phase, participants completed a confidential online survey, assessing various social-psychological factors. Demographics were recorded in an exit survey at the end of the experiment. Afterward, participants were thanked, debriefed, and confidentially paid their earnings. Each session lasted approximately 2 hours. More detailed information, such as the experiment instruction screens (S1 Appendix 2.1), survey questions (S1 Appendix 2.2), and a sample research protocol (S1 Protocol) can be found in the supplementary materials in OSF. The foraging software (v2021.08) used for this experiment is archived on Zenodo.

### Common-pool resource dilemma

**Fig 1:**
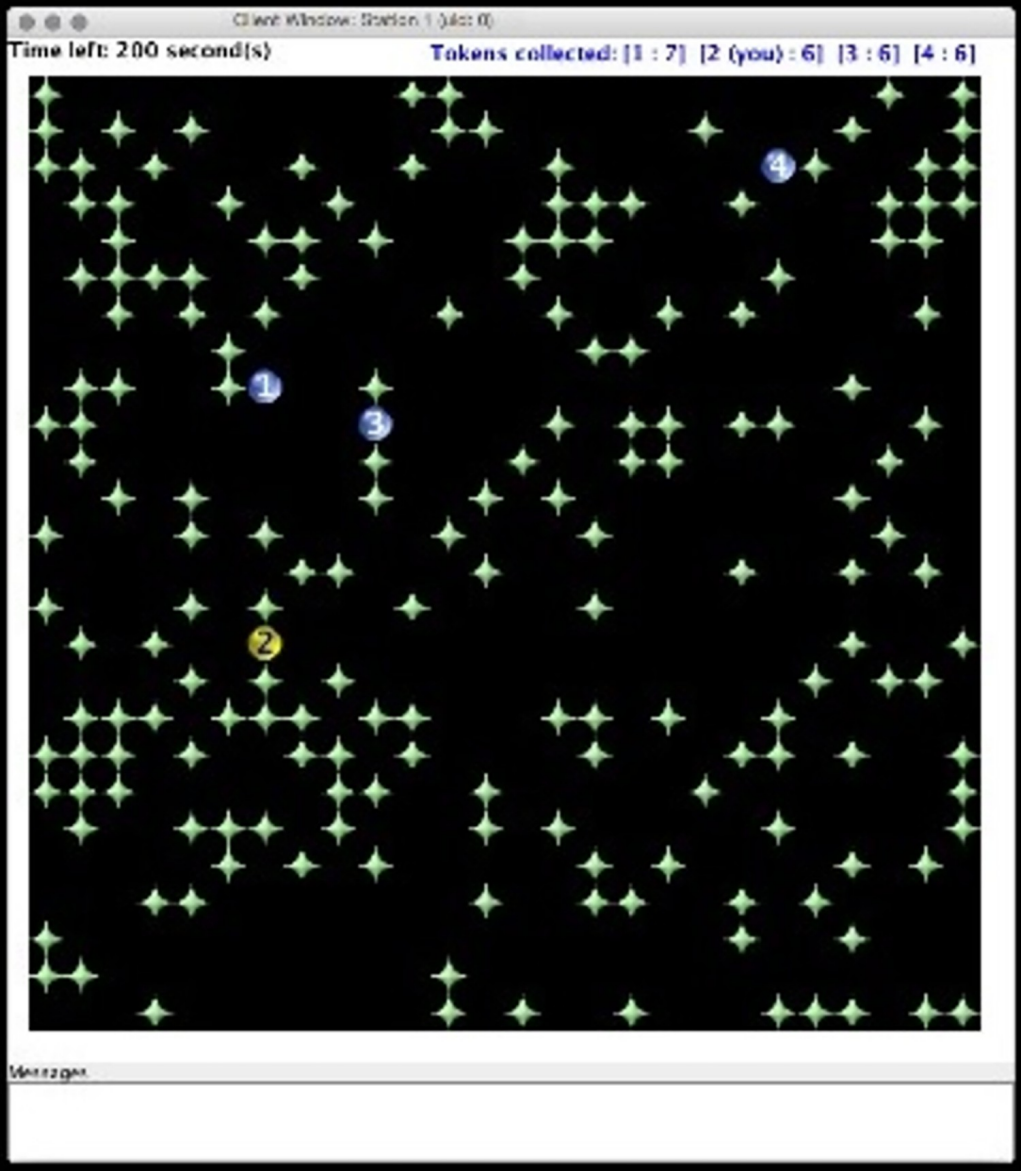
Foraging task. Four players (circles) collect resources (“star” tokens) worth $0.02 each in a real-time spatial resource dilemma. Players’ in-round total harvests appear at top right (e.g., Player 1: 7 tokens). When enabled, players communicate by typing messages in the “messages” window. When enabled, they may sanction individual players by pressing the number key corresponding to the targeted player’s Player Number (1–4).

We used a foraging task [18] to create the resource dilemma. During each round of the foraging task, group members accessed a shared resource pool consisting of tokens (“plants”), which were worth $0.02 each (Fig 1). The playing field was a 26 × 26 grid, and 25% of the field was randomly populated with 169 tokens at the beginning of each round. The tokens increased in number (“grew”), spreading to adjacent empty sections of the grid based on localized density (denser clusters produce greater growth chances). Each round lasted four minutes (240 seconds), meaning that substantially more tokens could be grown and harvested if the resource pool was properly managed. However, the tokens stopped growing, and the resource pool collapsed entirely, if all the tokens were collected (“harvested”).

The foraging task was played over a series of rounds to observe learning and performance. The optimal conservation strategy is to delay harvesting until the last 2 minutes of the round, and then harvest steadily in a checkboard pattern, thinning the clusters until the last 30 seconds of the round. This conservation strategy typically produces 548 tokens, allowing each person to collect approximately 137 tokens ($2.47) each round, for a total of 1,233 tokens ($24.66) over the course of 9 rounds (a typical experiment length). However, poor resource management often results in the resource collapsing within the first 30–45 seconds, yielding approximately 232 tokens per round, which is 58 tokens ($1.16) for each individual, and a total of approximately 522 tokens ($10.44) overall (see S1 Appendix1.0).

### Experimental treatments

In total, 72 groups participated in this experiment. The experiment consisted of 9 rounds, which were divided into 3 phases (3 rounds per phase). Each group was randomly assigned to one of the 6 possible experimental treatments (Table 1). The treatments were designed to test the effects of different initial regulatory environments (none/absent, lax, abuse) and facilitation (guiding principles of restorative justice) on the ability of groups to learn from prior regulatory failure. To emulate different regulatory environments, we altered starting conditions by manipulating the onset of communication and peer sanctioning, as well as the presence/absence of facilitation.

#### Communication

Communication was achieved via a built-in text messaging (chat) system. When communication was allowed, group members could chat for 6 minutes before each round; they could also chat during each of those rounds. All groups were instructed to use the chat as an opportunity to “consider potential strategies to manage the tokens.” We provided this prompt to ensure that unfacilitated groups and facilitated groups, which received additional guidance, each considered conservation strategies, ensuring that any potential differences of communication by treatment are due to facilitation on restorative justice principles, not unintended emphasis on conservation strategies across treatments. We instructed all groups that they could discuss as little or as much as they want but could not discuss side payments (outside the experiment), reveal their identity or personal information, or make physical threats.

#### Penalties

Peer sanctioning was achieved by a built-in costly sanctioning system, which allowed players to pay $0.02 (1 token) of their own earnings during a particular round to place a $0.04 (2 token) “monetary penalty” on another player that round. Sanctions were implemented in real time during the round and visible to all participants. Participants were informed that they could use monetary penalties as little or much as desired, as long as they had sufficient funds. Participants were not told when, why, or how much to use monetary penalties. The decision to use sanctions, if at all, was left to each participant’s discretion.

#### Facilitation

Half of the treatment groups received additional instructions to facilitate communication about the use of peer sanctions. Guidance was based on principles of restorative justice derived from Elinor Ostrom’s institutional design principle for graduated enforcement systems. These design principles commonly involve mechanisms for group “hearings” (multilateral communication, fact-seeking), clarification and justification, conflict resolution, reconciliation, and adjustments to accommodate serious concerns caused by current institutional arrangements [19, 35]. Specifically, facilitated groups were instructed to: (a) discuss whether, how, and why to use “monetary penalties,” (b) discuss any penalties that were used, giving the sanctioner and sanctioned equal opportunity to discuss why they sanctioned or defected, and (c) listen well, refrain from uncivil language, and try to constructively resolve any concerns or disputes about the group conservation strategy or its enforcement.

#### Social-psychological measures

We measured two key social-psychological constructs, restorative justice and enforcement goals, after each treatment group’s primary treatment phase (i.e., Phase A; Table 1). These measures were used to clarify potentially critical perceptions and goals specifically related to enforcement systems. These items were assessed on 7-point, Likert-type scales ranging from 1 (strongly disagree) to 7 (strongly agree). We also administered additional measures, assessing perceptions of procedural justice/self-determination, cooperative motivation (e.g., rule internalization, social pressure), group cohesion (e.g., trust), and basic perceptions of the dilemma (e.g., chaos) and need satisfaction (e.g., security, equity). We did not have strong predictions for the latter measures; these were included as additional potential descriptors to clarify potential treatment effects, in keeping with prior studies [9, 10]. However, as detailed in the supplementary materials (S1 Appendix 4.0), and indicated in the relevant results sections, we did not observe differences among treatment groups on these measures. Therefore, they are not discussed here.

*Perceived restorative justice*. In the context of rule enforcement, restorative justice is commonly conceptualized as including three major features: responsiveness, legitimization, and restitution [19, 31, 32, 36]. We used three items (α = .86) to measure perceived responsiveness (discussing whether/how to use sanctions, modifying existing arrangements to address concerns with enforcement; e.g., “If someone had a problem or concern with the way we were using monetary penalties, the group did a good job of discussing how to fix it”). Two items (α = .90) assessed legitimization (justification of enforcement and fair use; e.g, “The way my group used monetary penalties felt justified and legitimate”). Two items (α = .94) measured restitution (multi-lateral communication between sanctioners and the sanctioned; e.g., “The group did a good job of giving the person who was penalized an opportunity to discuss the situation and explain their point of view”).

*Enforcement goals*. To understand why group members may want (or not want) to use monetary penalties, we asked them about their potential goals. Three items (α = .70) measured perceived necessity for using monetary sanctions (e.g., “I felt that I did not need to use them”). One item assessed desire to prevent defection (“I wanted to prevent individuals that might disobey the group’s strategies/agreements from doing so”). One item assessed a goal to punish violators (“I wanted to punish or penalize group members that disobeyed the group’s token management strategies/agreements”). One item assessed desire to gain control (“I wanted to gain more control over the situation”). We aggregated these three items into a single indictor, representing the desire to use penalties to prevent-punish-gain control (PPC; α = .73), commonly inferred in enforcement theory [6, 55]. Finally, two separate items assessed desire to seek revenge (“I wanted to get revenge on someone in the group”) and fear of revenge-seeking (“I was worried that others would seek revenge on me, if I used monetary penalties on them”), which are common issues observed in prior peer-sanctioning studies [16, 18].

#### Communication coding

We developed a novel and updated version of DeCaro’s (2020) codebook to code in-game communication (chat messages). Our updates were designed to identify constitutional decisions, conservation agreements, enforcement systems (ES), and conceptual understanding of enforcement (CUE; see S1 File for details). Coders collaborated to organize chat into sections and identify collective decision events. Due to their complexity, conservation agreements were coded separately until all coders reached consensus. For enforcement, a second coder separately coded CUE and ES to compare with the primary coder, so that interrater reliability could be determined; interrater reliability was high (CUE: *Kappa* = .78; ES *Kappa* = .86). These coding practices align with accepted methods in this research domain [56].

*Conservation agreements*. To code each group’s conservation agreements, we identified the strategies they mentioned and agreed upon during their constitutional decision-events. In keeping with prior studies, a proposed conservation strategy was considered an agreement if at least two group members stated their support for the strategy [10, 57]. The coded categories (0–4) are described in the online supplement (S1 Appendix 3.1). They increase in complexity and optimality: (0) none (i.e., no strategy), (1) slow harvest or private property, (2) slow harvest and private property, (3) cultivate clusters, (4) checkerboard harvest pattern. We tracked changes to the conservation strategies over time (i.e., communication rounds). We also identified and recorded the final conservation strategy adopted by each group.

*Enforcement systems (ES)*. To our knowledge, prior studies have not systematically coded enforcement systems created by self-governing groups in prior experiments. We therefore developed the following categories (0–4) based on theory [55, 58], empirical case studies of community-based self-governance [19, 39], prior laboratory experiments [6, 18], and direct observation in the current experiment (S1 Appendix 3.2). We considered an enforcement system as established if at least two group members stated their support of a particular proposal during constitutional decision events about enforcement and monetary penalties:

(0) **None.** There is no formal enforcement system or agreement whatsoever; none proposed and/or agreed upon.
(1) **Do Not Use.** Group members actively decide (e.g., by voiced consensus, majority vote) not to use monetary penalties; actively decide to rely on voluntary agreements with no formal enforcement.
(2) **Independent Sanctions.** Group members decide that individuals may independently use penalties to punish individuals who violate the group’s conservation or enforcement agreement(s).
(3) **Coordinated Punishment.** Group members decide to coordinate their penalties to deter or punish anyone who violates the group’s agreement(s). Two or more players agree to jointly penalize violators.

We tracked changes to the enforcement system(s) across each communication round and recorded each group’s final enforcement system.

*Conceptual understanding of enforcement (CUE)*. We are also unaware of any experimental studies that have coded group members’ conceptualization and comprehension of enforcement systems. Therefore, we relied on the aforementioned sources (e.g., theory, case studies, direct observation) to devise coding categories (S1 Appendix 3.4). Specifically, we based CUE scores on each group’s discussion of the advantages and disadvantages, rationales, perceived effects, and believed effects or implications of using monetary penalties. Groups often discussed the merits and implications of monetary penalties within constitutional decision events. However, groups also discussed these ideas outside specific events. Therefore, when coding CUE, we included any statements made about monetary penalties or enforcement.

The coding categories (0–3) are intended to represent increasing sophistication in the group’s comprehension, ranging from no apparent understanding (i.e., fails to discuss enforcement or cannot provide any rationale), to acknowledging the costs or drawbacks of using penalties but also acknowledging potential benefits and ways to mitigate the costs.

(0) **None.** Did not discuss enforcement (use of monetary penalties) or discussed it but could not (or did not) state a rationale/purpose for its use. For example: P1, “Why would we want to use monetary penalties?” P2, “I have no idea.”
(1) **Too Costly/Harmful.** Members only state that monetary penalties are too costly (financially) or harmful. For example: P1, “Monetary penalties are stupid. We should not use them, because it only hurts us.” P2, “yeah for real.”
(2) **Useful Deterrent.** Members additionally state that monetary penalties can be useful to correct or prevent (deter) defections (i.e., violations of group agreements). For example: P1, “I don’t really like them [penalties] unless we are trying to keep each other from causing the tokens not to regenerate.” P2, “same.”
(3) **Efficient Deterrent/Credible Threat.** Members additionally state or act upon the assumption that monetary penalties are more beneficial/effective (i.e., more potent) and/or efficient (i.e., less costly to any single individual) when they are coordinated. For example: P3, “But who would administer the fine? Everyone?” P1, “Yeah, itd hurt more And you cant retaliate against 3 people as easily.”

We recorded CUE across communication rounds, as well as final CUE for each group.

### Statistical methods and analyses

The research design, procedures, and materials for this study were reviewed and approved by the University’s human subjects institutional review board to ensure ethical treatment of participants and their data (IRB #16.1232). Data collection occurred from September 6, 2018 to September 27, 2019. As previously noted, we obtained a sample of 288 participants, resulting in 11–14 groups per treatment (Table 1). This sample size matches prior research using the foraging task [9, 10, 18]. We used G*Power (version 3.1.9.7) [59] to conduct a post-hoc sensitivity analysis with our obtained sample and desired power of at least 80%. Our tests comparing facilitated vs. unfacilitated treatments could detect effects as small as *dz* = 0.28; planned comparisons between individual treatments could detect moderate effects (*d* = 0.60). Thus, obtained power was sufficient to investigate our key hypotheses.

Our core hypotheses predicted overall benefits of facilitation (principles of restorative justice) compared to unfacilitated communication. We also predicted a specific advantage among paired treatments, comparing the unfacilitated and facilitated versions of each treatment (e.g., PCN vs. PFN). Therefore, we used planned comparisons and 1-tailed tests (α < .05) in tests of significance [60]. We used IBM SPSS version 29 for analyses. Descriptive statistics for the primary variables are reported in Table 2 (see S1 Table in S1 Appendix for secondary variables). Preliminary analyses revealed substantial skew (non-normality) and heterogeneity of variance in the data. Therefore, unless otherwise noted, we used *Mood’s Median Test* to test our core hypotheses, because this test is most robust against these considerations [61, 62]. We used the *Hodges-Lehman* method to estimate confidence intervals for nonparametric tests [63] and report *r* (= *Z/(√Nobs*)) for effect size estimates [64].

**Table 2:** Descriptive statistics (Primary variables).

	T1 (CNN)	T2 (FNN)	T3 (NCN)	T4 (NFN)	T5 (PCN)	T6 (PFN)
Variable	*Md* (*IQR*)	*Md* (*IQR*)	*Md*(*IQR*)	*Md*(*IQR*)	*Md*(*IQR*)	*Md*(*IQR*)
Net Tokens 1	346.33 (50.00)	374.33 (72.34)	204.33 (15.00)	215.17 (24.83)	211.67 (34.33)	208.83 (34.17)
Net Tokens 2	351.33 (199.67)	392.00 (108.33)	361.67 (73.00)	395.17 (39.00)	351.67 (67.17)	420.50 (51.25)
Net Tokens 3	321.33 (191.00)	330.67 (134.66)	390.00 (216.00)	401.66 (77.51)	353.67 (126.99)	421.50 (81.75)
DDMI	3.50 (0.42)	3.67 (0.60)	3.33 (0.71)	3.67 (0.34)	3.80 (0.69)	3.90 (0.38)
Conservation Strategy	2.00 (2.00)	4.00 (1.00)	2.00 (2.00)	2.00 (3.00)	3.00 (2.00)	2.50 (2.00)
Conceptual Understanding of Enforcement (CUE)	0.00 (1.00)	1.00 (1.00)	1.00 (1.00)	1.00 (2.00)	0.00 (1.00)	2.00 (3.00)
Enforcement System (ES)	1.00 (0.00)	1.00 (1.00)	1.00 (0.00)	1.50 (1.00)	1.00 (1.00)	2.00 (2.00)
Necessity of Sanctions	2.25 (1.08)	2.00 (0.50)	2.00 (1.17)	2.38 (0.85)	2.92 (0.79)	3.08 (0.90)
Protect, Punish, and Control (PPC)	3.42 (1.42)	3.25 (1.25)	3.33 (1.08)	3.67 (1.19)	3.75 (1.46)	3.96 (1.65)
RJ Responsiveness	6.25 (1.08)	6.50 (0.58)	6.25 (1.08)	6.54 (0.69)	5.25 (2.04)	5.96 (1.37)
RJ Legitimacy	5.75 (1.00)	6.00 (2.50)	5.25 (1.63)	5.94 (1.06)	5.13 (0.88)	5.75 (1.31)
RJ Restitution	4.75 (0.92)	4.75 (1.50)	4.50 (0.75)	4.69 (0.84)	4.00 (2.06)	4.94 (0.91)

*Net Tokens 1–3* (average net tokens collected by the group during Phases 1–3). *DDMI* (democratic decision-making index). *Conservation Strategy* (group’s final conservation strategy). *CUE* (group’s final conceptual understanding of enforcement score). *ES* (group’s final enforcement system). *Necessity of Sanctions* (group’s perceived necessity for sanctions). *Protect*, *Punish*, *and Control* (group’s desire to use penalties to protect, punish, and gain control). *RJ Responsiveness*, *Legitimacy*, *Restitution* (group’s perceived restorative justice: responsiveness, legitimacy, restitution). *Md* (median). *IQR* (interquartile range).

We first report the results for cooperation. We then report the results for groups’ collective decision-making processes (i.e., democratic decision-making), conservation agreements, and enforcement systems. Afterward, we report the results of their conceptual understanding of enforcement (CUE) and the evolution of their enforcement systems (ES). Finally, we report perceived restorative justice (including legitimacy) of enforcement, providing additional information into the enforcement systems that groups created.

## Results

As a reminder, condition names were denoted by abbreviations signifying the treatment that occurred during each of the three experiment phases: *N* (no communication or penalties), *C* (unfacilitated communication with penalties), *F* (facilitated communication with penalties), and *P* (penalties with no communication). For example, groups in the CNN condition (“communication followed by no communication”) completed Phase 1 with basic communication and penalties, then had no further treatments (i.e., no communication or penalties) for the final two Phases 2 and 3. In contrast, groups in the FNN condition (“facilitated communication followed by no communication”) had these same treatments, except they also received guidance (facilitation) during communication (see Table 1 for review).

### Cooperation

To assess cooperation, we examined net tokens harvested (average tokens harvested each phase, minus costly penalties). Cooperation improved after communication for groups that began without communication (NCN, NFN, PCN, PFN, *n =* 50; before *Md* = 211.15, *IQR* = 31.00, after *Md* = 378.34, *IQR* = 85.91); *Md Diff =* 153.33, *95CI*[134.50, 170.17]; *Wilcoxon signed-rank test*, *Z* = 5.78, *p* < .001, *r*(1) = 0.82. This effect illustrates the well-documented benefit of communication [e.g., 10, 18, cf. 65, 66].

**Fig 2:**
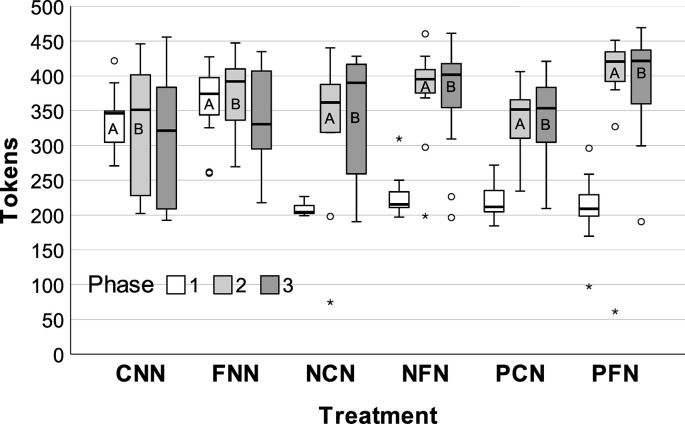
Median net tokens collected by groups as a function of phase and treatment. Phase A and B (during/after communication) are compared when examining treatment effects. Error bars = *95CI*.

However, our core hypotheses pertain to the effects of initial regulatory failure and facilitated communication during the communication and enforcement phase (Phase A) and immediately after that phase (i.e., Phase B), when communication and peer sanctions were no longer possible (Table 1, Fig 2). We therefore examined facilitated vs. unfacilitated treatment groups during Phase A and Phase B, as well as potential change across these phases.

During Phase A, the facilitated groups (FNN, NFN, PFN) outperformed the unfacilitated groups (CNN, NCN, PCN): unfacilitated *Md* = 348.00 (*IQR* = 67.00), facilitated *Md* = 397.67 (*IQR* = 51.50); *Md Diff =* 48.00, *95CI*[23.66, 71.00], *Md test* = 16.07, *p* < .001, *r*(1) = 0.47. Furthermore, each facilitated treatment outperformed its unfacilitated counterpart: *Md Tests* ≥ 6.76, *ps* ≤ .033 (e.g., PFN vs. PCN: *Md test* = 23.09, *p <* .001). PFN, the condition which began with penalties (and no communication) followed by facilitated communication, performed the best (*Md* = 420.50, *IQR* = 51.25), followed by NFN (*Md* = 395.17, *IQR* = 39.00, *p =* .018) and FNN (*Md* = 374.33, *IQR* = 72.34, *p =* .006). CNN (*Md* = 346.33, *IQR* = 50.00) and PCN (*Md* = 351.67, *IQR* = 67.17) performed worst.

During Phase B, most treatment groups improved, but not significantly: Phase A *Md* = 374.00 (*IQR* = 82.92), Phase B *Md* = 386.67 (*IQR* = 111.16); *Md Diff =* 12.67, *95CI*[-12.34, 13.51], *Wilcoxon signed-rank test*, *n* = 72, *Z* = 0.12, *p* = .902, *r*(1) = 0.01. Hence, groups maintained consistent levels of cooperation after communication and enforcement ended.

Thus, as hypothesized, facilitated groups cooperated better. PFN appeared to perform better overall, followed by NFN and FNN. Importantly, NCN and PCN, the two conditions that began with the most distinct types of regulatory failure (i.e., absent, abuse) and lacked facilitation, performed worst. Collectively, these results align with our hypotheses that initial regulatory failure (especially regulatory abuse) is important for group learning but may need to be scaffolded with guidance on principles of restorative justice to ensure positive outcomes. We ruled-out democratic process, motivation, and conservation strategy as core explanations. We describe this information next.

### Democratic decision-making (DDMI)

Democratic decision-making is an institutional design principle associated with improved collective decision-making, institutional legitimacy, acceptance, and cooperation [9, 12, 32]. As previously noted, democratic decision-making has been shown to promote provisioning and acceptance of enforcement systems in some situations [2, 9, 12]. We determined whether groups made decisions democratically by identifying their constitutional decision events. These are events where group members deliberate and decide fundamental institutional arrangements, such as their conservation agreements and enforcement systems. The number of group members that contribute to each decision can be used as an indicator of democratic decision-making, and a proxy for procedural justice and self-determination [10, 67]. Therefore, to analyze democratic decision-making, we counted how many group members contributed to each decision on average, creating a democratic decision-making index (DDMI, S1 Appendix 3.4).

Overall, the treatment groups achieved high levels of democratic decision-making. On average, approximately 3.67 of the four group members (*Md DDMI =* 3.67, *IQR* = 0.52) contributed to their groups’ constitutional decisions. Furthermore, DDMI did not differ by facilitation: unfacilitated *Md* = 3.60 (*IQR* = 0.67), facilitated *Md* = 3.67 (*IQR* = 0.46); *Md Diff =* 0.13, *95CI*[0.00, 0.33], *Md Test* = 0.97, *p* = .324, *r*(1) = 0.09.

Thus, democratic decision-making, while likely essential to overall improvement [10, 19], was not a determining factor for the observed treatment differences associated with facilitation. Groups also reported high perceived procedural justice/self-determination (*Md PJSD* = 6.37 on 7-pt scale, IQR = 0.38) regardless of facilitation: unfacilitated *Md* = 6.32 (*IQR* = 0.43), facilitated *Md* = 6.23 (*IQR* = 0.38); *Md Diff =* 0.07, *95CI*[-0.14, 0.14], *Md Test* = 0.62, *p* = .803, *r*(1) = 0.00. Treatment groups reported similar perceptions of the dilemma, need satisfaction, cooperative motivation, and group cohesion (e.g., trust), ruling out these as possible psychosocial explanations for the observed effect of facilitation as well (S1 Appendix 4.1) [9, 10, 68].

### Conservation agreements

Most groups (96%) created a conservation agreement to manage the resource pool. The most common agreements were checkerboard (32 groups, 44%) and slow-plus-private-property (21 groups, 29%). However, with the exception of FNN vs. CNN, the treatment groups did not differ in the conservation agreements they used (S1 Appendix 4.2). Therefore, conservation strategy cannot be the critical determining factor between treatments.

### Enforcement (ES)

Only 23 groups (33%) developed an enforcement system: 9 (13%) failed to discuss penalties or did not reach consensus (*none*); 39 (54%) chose not to use penalties (*do not use*); 15 (21%) used independent sanctions; 9 (13%) used coordinated sanctions. Groups that had an enforcement system to enforce their conservation agreements cooperated better (no enforcement system *Md* = 357.67 tokens (*IQR* = 79.17); any enforcement system *Md* = 397.50 tokens (*IQR* = 55.34), *Md Diff* = 27.66, *95CI*[1.34, 54.67], *Md Test* = 6.25, *p* = .012, *r*(1) = 0.27).

**Fig 3:**
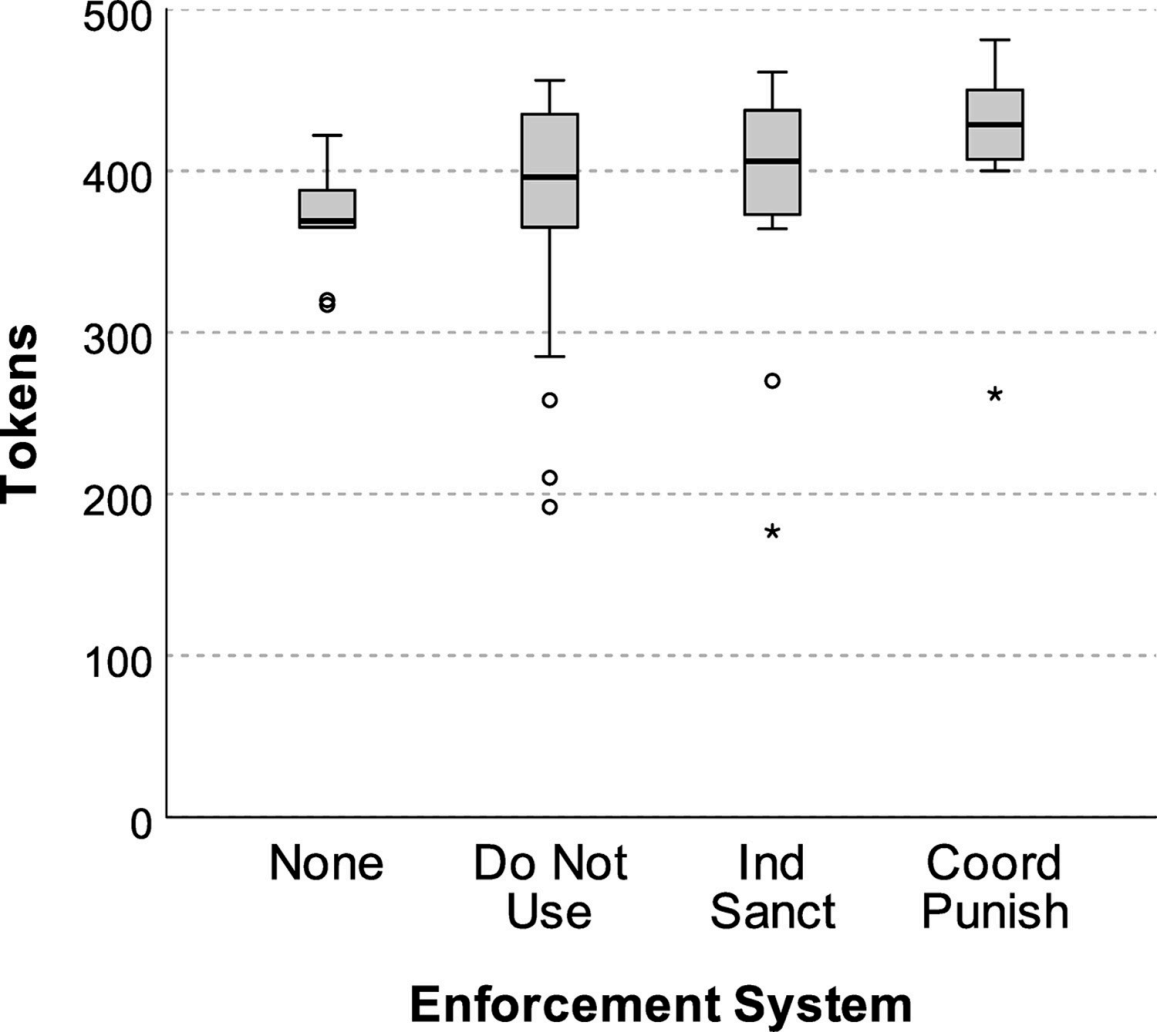
Net average (median) tokens collected as a function of enforcement system. An extreme outlier in the NCN treatment (the only group to achieve net zero tokens) was excluded from analysis. Error bars = *95CI*.

**Fig 4:**
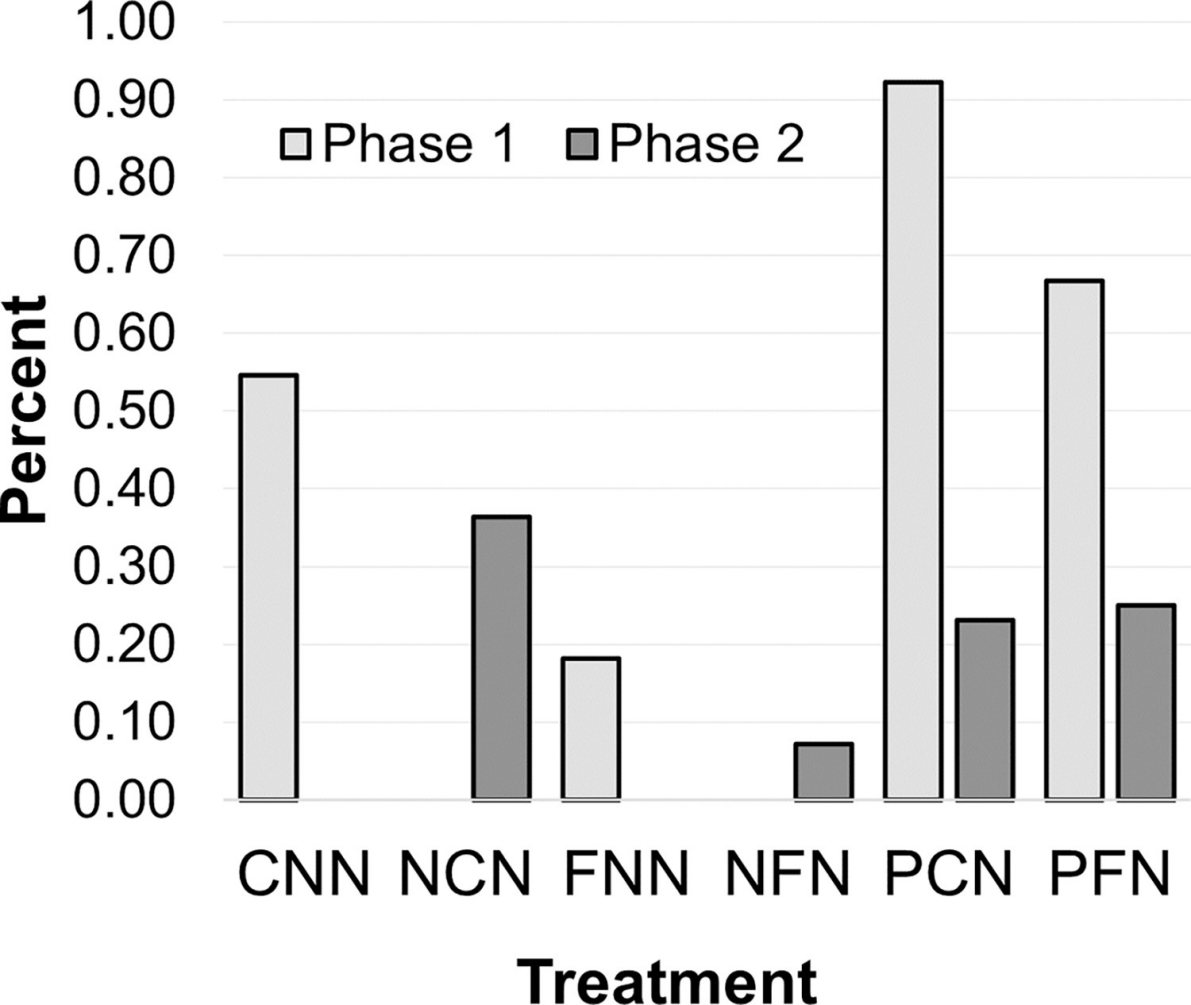
Percentage of groups that sanctioned during Phase 1 and 2. Groups with no communication but initial sanctioning (PCN, PFN) were more likely to sanction. Sanctioning decreased over time.

During communication, most groups achieved peak cooperation (conservation performance) in the final round of communication (i.e., Round 3 of Phase A). During this final round of communication (Fig 3), coordinated punishment (*Md* = 428.50, *IQR* = 46.50) outperformed all other types of enforcement (*Md Tests* ≥ 12.86, *ps* ≤ .029). The total number of penalties also tended to decrease each round, reaching its lowest frequency during the final round of communication (Fig 4). These findings suggest that these particular groups succeeded in creating a credible threat of enforcement [6, 19], which elicited fairly robust compliance without undermining voluntary cooperation in Phase B.

### Evolution of enforcement and conceptual understanding

There were 350 sanctions total across all treatments. PFN sanctioned the most (195 penalties, 58%), followed by NCN (70, 20%) and PCN (67, 19%). PFN and PCN had the highest percentage of groups that used at least one sanction in each treatment (Fig 4; PCN: 12 groups, 92%; PFN: 8, 67%), and this occurred primarily during their initial period of enforcement without communication (i.e., Phase 1). Thus, groups that began Phase 1 with penalties (PCN and PFN) created initial circumstances of regulatory abuse as intended in the research design.

Facilitated treatment groups, overall, exhibited more sophisticated enforcement systems (ES), facilitated *Md* = 2.00 (*IQR* = 1.00), unfacilitated *Md* = 1.00 (*IQR* = 0.00), *Md Test* = 11.12, *p* < .001, *r*(1) = 0.36. Approximately half (51%) of facilitated groups developed and used independent or coordinated sanctioning systems, compared to just 14% of unfacilitated groups. Facilitated groups also had more sophisticated conceptual understanding (CUE), facilitated *Md* = 1.00 (*IQR* = 2.00), unfacilitated *Md* = 0.00 (*IQR* = 1.00)), *Md Test* = 11.74, *p* < .001, *r*(1) = 0.37.

**Fig 5:**
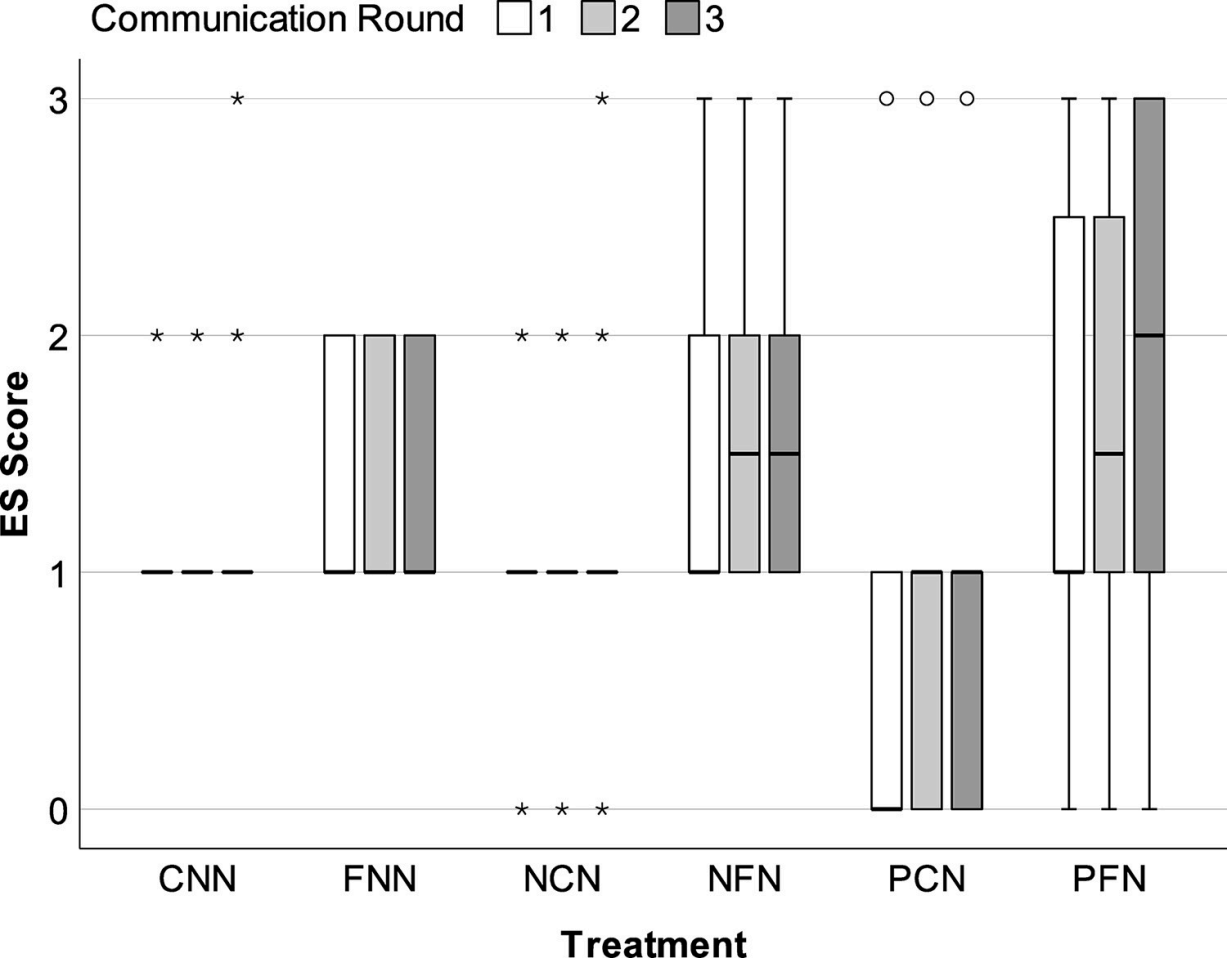
Evolution of enforcement system. Median enforcement system (ES) score during each communication round (i.e., Rounds 1–3 of Phase A). Error bars = *95CI*.

**Fig 6:**
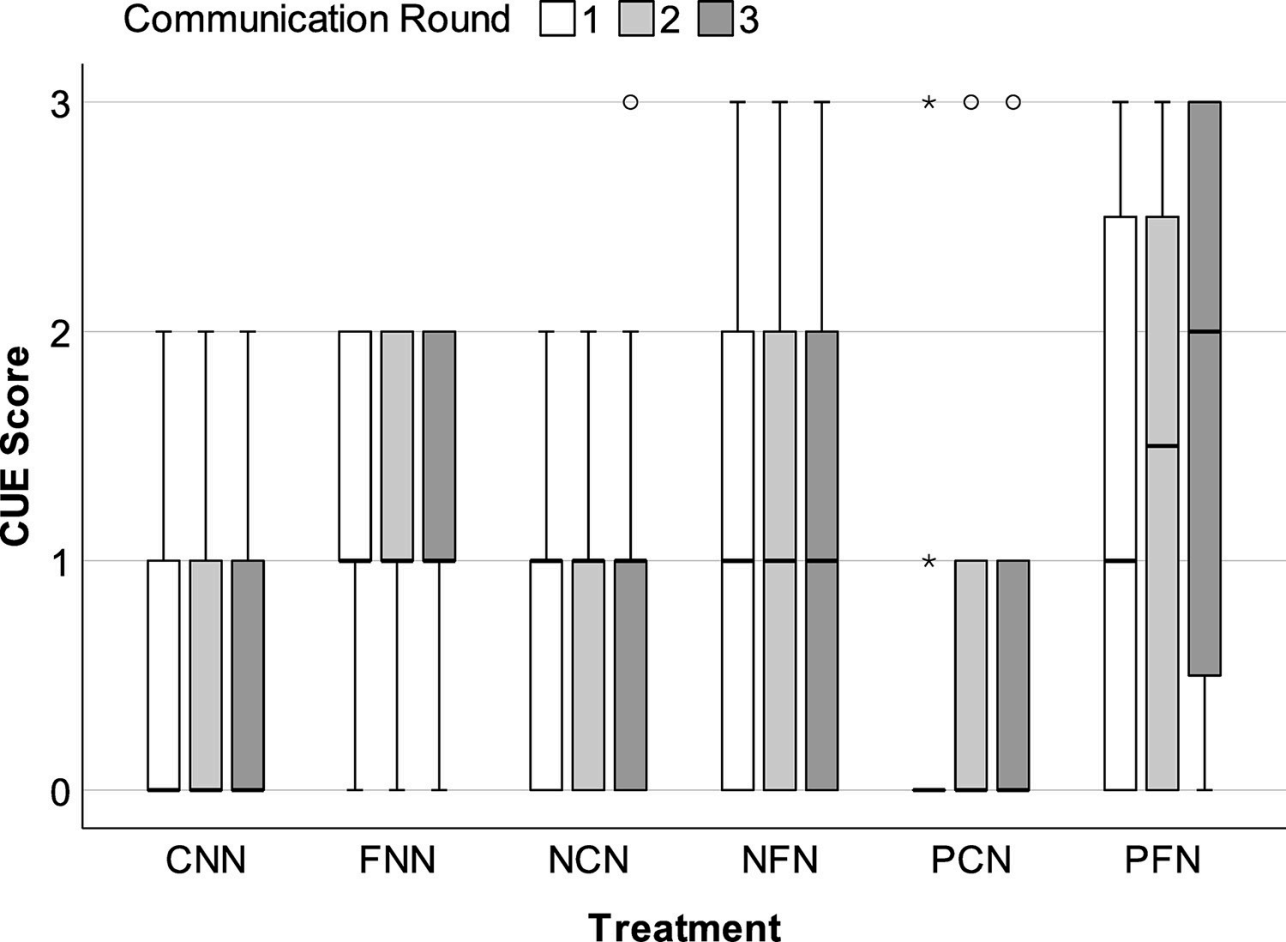
Evolution of conceptual understanding of enforcement. Median conceptual understanding of enforcement (CUE) score during each communication round (i.e., Rounds 1–3 of Phase A). Error bars = *95CI*.

The evolution of each treatment group’s enforcement system(s) (ES) and conceptual understanding of enforcement (CUE) during each round of communication is illustrated in Figs 5 and 6. Most groups’ ES and CUE scores plateaued during the first round of communication (i.e., Round 1 Phase A). Specifically, most groups failed to acknowledge any potential benefit for using monetary penalties (i.e., CUE = 1 *too costly/harmful*) and collectively decided not to use penalties to enforce agreements (i.e., ES = 1 *do not use*). These groups only mentioned the financial costs of sanctions, harm to each person’s net earnings, and/or the potential for regulatory abuse (e.g., revenge-seeking/retaliatory penalties).

However, PFN, the treatment condition that began with penalties (i.e., abuse) followed by facilitation, showed improved ES and CUE scores over time. By the final round of communication (Round 3), PFN exhibited higher ES scores (*Md* = 2.00, *IQR* = 2.00) than the other treatments (*Mds* = 1.00, *IQRs* ≤ 1.00, *Md Tests* ≥ 7.77, *ps* ≤. 025), followed by NFN (*Md* = 1.50, *IQR* = 1.00, *Md Test* = 2.64, *p* = .125). In total, 58% of PFN groups used independent (25%) or coordinated sanctioning systems (33%). This rate contrasts sharply with its direct comparator, the PCN condition, which also began with penalties (i.e., abuse) but lacked facilitation. Specifically, no groups in the PCN condition used independent sanctions (0%) and only 8% used coordinated sanctions (Table 3). In fact, 46% of the PCN groups had no formal ES of any kind (i.e., ES = 0 *none*). Finally, PFN groups also exhibited greater conceptual understanding (CUE). As communication rounds progressed, these groups went from primarily discussing the potential costs and harms of monetary penalties to discussing ways to use coordinated penalties to maximize deterrent efficiency, reduce individual costs, and mitigate potential harms of peer sanctioning.

**Table 3:** Final enforcement system.

	None	Do Not Use	Independent	Coordinated
CNN	0 (0%)	9 (82%)	1 (9%)	1 (9%)
FNN	0 (0%)	6 (55%)	5 (46%)	0 (0%)
NCN	2 (18%)	7 (64%)	1 (9%)	1 (9%)
NFN	0 (0%)	7 (50%)	5 (36%)	2 (14%)
PCN	6 (46%)	6 (46%)	0 (0%)	1 (8%)
PFN	1 (8%)	4 (33%)	3 (25%)	4 (33%)

Number of groups that used particular enforcement systems in each treatment. None (no enforcement system). Do Not Use (decided not to use peer sanctions). Independent (individuals will independently sanction defectors). Coordinated (group members will jointly sanction defectors). Treatment labels are consist of three letters that signify the treatment during each of the 3 experiment phases (Phases 1–3). N (no communication or penalties). C (communication with penalties). F (facilitated communication with penalties). P (penalties with no communication). For example, CNN (Phase 1: communication with penalties, Phase 2–3 no communication or penalties).

### Enforcement goals

When surveyed about the perceived necessity for using monetary penalties, the two groups that began with penalties, PCN and PFN, reported greater necessity (PCN *M* = 2.96, PFN *M* = 3.06, *SEs* = 0.18 vs Others *Ms* ≤ 2.42, *SEs* = 0.19), *t*s(66) ≥ 1.29, *ps* ≤ .007, *ds* ≥ 0.86). The heightened perceived necessity for monetary penalties is presumably due to PCN and PFN’s higher exposure to penalties during Phase 1, *r(70)* = 0.42, *p* < .001 [69, 70]. PFN also reported a stronger desire (*M* = 4.21, *SE* = 0.24) to use penalties to prevent defection, punish violators, and gain control over the situation than the other treatments (*Ms* ≤ 3.64, *SEs* = 0.25), *t*s ≥ 1.13, *ps* ≤ .050. Overall, the treatment groups reported equally low desire for revenge (*Mds* ≤ 2.25, *IQR*s = 0.88 to 2.19), *p*s ≥ .180 and moderate fear of revenge (*Mds* ≤ 5.25, *IQR*s = 1.75 to 3.25), *p*s ≥ .107. This finding indicates that most group members did not seek revenge but feared it somewhat.

### Perceived restorative justice

**Fig 7:**
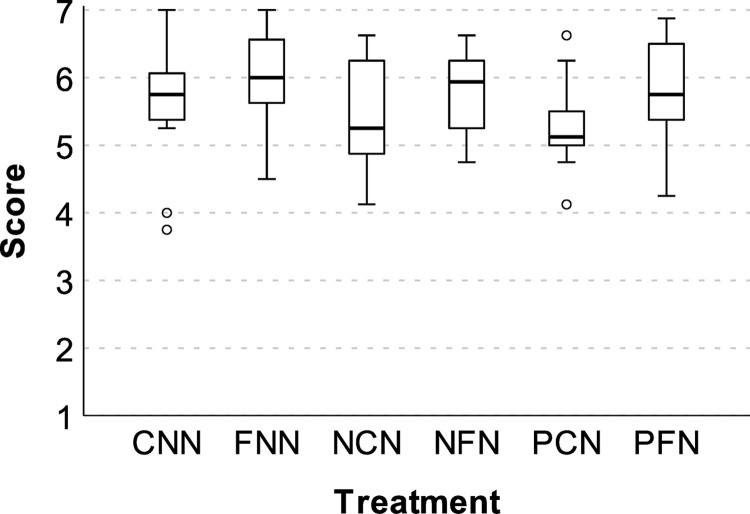
Perceived legitimacy of enforcement. Error bars = *95CI*.

Overall, the treatment groups reported high levels of restorative justice in terms of responsiveness and legitimacy. However, facilitated groups reported the highest levels. Specifically, for responsiveness: facilitated (*Md* = 6.42, *IQR* = 0.79) vs. unfacilitated (*Md* = 6.00, *IQR* = 1.33), *Md Diff* = 0.42, *95CI*[0.08,0.75], *Md Test* = 5.55, *p* = .048, *r*(1) = 0.24. For legitimacy: facilitated (*Md* = 5.88, *IQR* = 0.94) vs. unfacilitated (*Md* = 5.50, *IQR* = 1.25), *Md Diff* = 0.38, *95CI*[0.08,0.75], *Md Test* = 5.69, *p* = .046, *r*(1) = 0.28. These effects were driven by an apparent boost to facilitated groups and deficit to PCN. Specifically, PCN (*Md* = 5.13, *IQR* = 0.88), which began with penalties and no communication (i.e., regulatory abuse), reported lower legitimacy (Fig 7) than each of the facilitated treatments, which received guidance on principles of restorative justice (*Mds >* 5.75, *IQRs* ≤ 1.63, *ps* ≤ .016).

A similar pattern emerged for restitution. In the current experiment, restitution refers to making amends for potential harms caused by penalties. When we restrict our examination to groups that actually used monetary penalties (i.e., group members sanctioned one another), facilitated groups report higher perceived restitution: facilitated (*M* = 4.95, *SE* = 0.25) vs. unfacilitated (*M* = 4.37, *SE* = 0.18), *t*(68) = 3.76, *p* = .032, *d* = 0.67. Thus, overall, facilitation was associated with greater perceived restitution.

Some treatments used too few sanctions to support reliable analysis of restitution for all individual treatments (Fig 4). However, medians for PCN and PFN—the treatments with the greatest number of penalties (PCN, *n* = 12; PFN, *n* = 9)—suggest a potential PFN advantage (PCN *Md* = 3.81 (*IQR* = 2.16), PFN *Md* = 5.25, (*IQR* = 1.63)), *Md Test* = 2.07, *p* = .154.

## Discussion

Modern democracies face significant challenges in designing effective enforcement systems. Enforcement systems frequently face problems of illegitimacy, misuse/abuse, and non-compliance. These problems emerge from deeply-rooted misconceptions about enforcement that hinder humanity’s ability to govern itself and sustain vital social-ecological systems [8, 13, 29]. Science struggles to explain when and how effective enforcement systems emerge, further hindering societal progress [7, 13, 23]. For example, two influential review articles by Bowles [8] and Elinor Ostrom [23] suggest that scientific confusion about the proper design and use of enforcement has resulted in decades of poorly designed enforcement systems that undermine, rather than improve, compliance and cooperation [13]. This is a chronic issue for many sectors of law enforcement and environmental regulation [7, 8, 28]. This confusion recently played out on a global scale as societies such as the U.S. struggled to regulate COVID safety behaviors, and populations increasingly rejected otherwise effective enforcement systems [11, 71].

In the current study, we sought to inform these issues by examining important cognitive and behavioral foundations of wise enforcement, group learning, and constitutional choice. To do so, we modified a standard common-pool resource experiment. First, we placed groups of naïve individuals who typically lack enforcement experience—undergraduate student participants—in treatments representing different types of initial regulatory failure. Second, we used Elinor Ostrom’s design principle for restorative justice to guide half of the groups, when they made decisions about how to use peer sanctions to enforce their agreements. We created a novel coding system to examine each treatment group’s learning process, as well as their constitutional decision-making process. This approach allowed us to track conceptual understanding of enforcement over time and relate this understanding to the enforcement systems that evolved. In support of our core hypotheses, we found that initial regulatory failure (especially abuse) was important for group learning and cooperation. However, groups needed to receive guidance in the form of restorative justice principles in order to enhance learning and performance.

### Evolutionary trajectories

Previous experiments have identified that naïve groups may eventually use enforcement systems (e.g., monetary penalties) to improve cooperation after many trials of failure without enforcement [e.g., 1, 6, 16, 18]. However, such experiments have not adequately explained why or how this pattern emerges from a learning and regulatory failure standpoint. And, they have not fully identified common trajectories. We identified three important evolutionary trajectories and social-learning processes that need proper description. These trajectories have different and potentially critical implications for group learning, institutional evolution, and performance.

#### Voluntary commitment

First, many groups tried to rely on voluntary cooperative agreements, with varying success. The differences in performance were driven by the type of initial regulatory failure and the presence or absence of facilitated communication. Specifically, treatment groups that started with lax or no enforcement and no facilitation (i.e., CNN, NCN) cooperated poorly, reducing their resource sustainability and group earnings.

The CNN treatment condition had their first round of communication before ever experiencing the resource dilemma as a collective. Our analysis of group discussion and constitutional decision-making indicates that these groups performed poorly because they entirely lacked experience with enforcement (good or bad) before they started to communicate about enforcement. Specifically, without prior enforcement experience, they saw no reason to use monetary penalties, and therefore could not effectively justify (legitimize) its use *a-priori*. Like many other treatment groups, they also tended to focus on the potential harms of enforcement. Therefore, they decided to rely purely on voluntary commitments. However, because these groups did not receive facilitation in the form of guidance on Ostrom’s restorative justice design principle to ensure more constructive discussion of enforcement, they failed to create well-conceived voluntary commitments. This misstep resulted in poor learning and cooperation.

The NCN treatment condition fared a bit better. This group had some experience with the resource management dilemma (Phase 1) before they were able to communicate. Because they entirely lacked any means of communication or enforcement during Phase 1, they witnessed firsthand the problems that can arise without any enforcement (i.e., absent enforcement). However, because they lacked facilitation, these groups also failed to robustly discuss enforcement and, therefore, created imperfect voluntary commitments. This also hindered their subsequent learning and performance.

In contrast to the two previous treatments, the FNN treatment condition received facilitation upfront during the first round of communication (and thereafter). FNN groups also tended to rely on voluntary commitments, but outperformed CNN and NCN. We observed that facilitated communication helped FNN groups deliberate about and, therefore, better conceive the rationales and objectives for their voluntary agreements (i.e., *why* they did not want to use penalties). We call this strategy, “mutual cooperation mutually agreed upon,” to contrast Hardin’s (1968) [5] classic idiom of collective enforcement, “mutual coercion mutually agreed upon.” This approach helped FNN groups achieve robust, yet moderate levels of cooperation and resource sustainability. However, similar to real-world groups involving citizens that fail to develop more sophisticated peer-sanctioning systems [6, 19], FNN’s groups of naïve students failed to recognize the advantages of costly enforcement. Voluntary cooperation rarely ensures that all individuals cooperate [6]. FNN’s lack of understanding prevented them from considering and, therefore, learning how to use peer sanctions to deter the remaining individuals who refused to cooperate voluntarily. This oversight limited their overall performance.

#### Abandonment and mutual coercion

The last two evolutionary trajectories emerged in PCN and PFN, the treatment conditions that began with initial regulatory abuse. Facilitation was the decisive factor. We first explain the common starting point for these conditions. We then explain why their evolution differed.

During Phase 1, the PCN and PFN treatment group members could use monetary penalties in the resource management dilemma, but they could not communicate. This lack of communication led them to use the penalties unwisely, without apparent rationale or coordination. This arrangement triggered spiteful sanctions, exacerbating initial conflicts that spilled over to later communication (Phase 2). Specifically, participants initially remained spiteful at the beginning of communication in Phase 2. This is the type of situation that is most in need of restorative justice [32, 34, 36]. The trajectory of the PCN and PFN groups differed because one group received guidance on restorative justice, and the other did not.

Specifically, the PCN groups, which did not receive guidance, struggled to reconcile and engage in constructive deliberation about enforcement. These groups fixated on their prior regulatory abuses and initial misconceptions, leading to abandonment of enforcement. Many of these groups (46%) failed to create enforcement systems of any kind; they were unable to reach any agreement about enforcement whatsoever (voluntary or otherwise). PCN groups also reported lower perceived legitimacy of enforcement, indicating that they could not justify the use of monetary penalties well, whenever they were used.

In contrast, PFN groups learned from their mistakes, developing fairly sophisticated collective enforcement systems, previously identified by Ostrom [6, 19]. Facilitated discussion helped these groups discover insights from their collective failure. In addition to discussing potential disadvantages of enforcement, they discussed the potential advantages. Specifically, they identified that they needed to use enforcement to achieve higher levels of cooperation by preemptively preventing potential defection and by deterring the remaining defectors. They were also more likely to discuss how to mitigate revenge-seeking, increase fairness, and improve efficiency and credible threat (i.e., deterrence force), specifically by banding together (i.e., coordinated sanctions).

Hardin 1968 [5] previously identified that mutual coercion is necessary for societal cooperation. However, Hardin and other classical theorists (e.g., Hobbes) [3] strongly predicted, based on economic rational choice theory, that regular individuals cannot discover these principles themselves or use communication to create enforcement systems [6, 7]. Instead, outside intervention by more enlightened leaders and policymakers is required (i.e., Leviathan). Hence, naïve university students, who typically lack enforcement experience, would not be predicted to develop such sophisticated systems of self-governance and enforcement. Our experiment, which was designed to emulate some basic aspects of real-world dilemmas, demonstrated that regulatory abuse, combined with some guidance on principles of restorative justice, helped naïve students to begin to discover these principles themselves.

Furthermore, PFN groups made this achievement while reporting greater perceived restorative justice. This accomplishment is remarkable, because PFN groups used more stringent peer sanctioning systems. Our results indicate that the PFN groups were able to use enforcement without undermining perceptions of legitimacy, procedural justice/self-determination, or internalized motivations. In other words, these groups achieved robust cooperation without undermining perceived fairness of the democratic process or crowding-out internal motivations. As we noted earlier, these are difficult achievements to secure in the laboratory or broader society [e.g., 12, 10, cf. 8, 23].

*Civic education and social learning*. Regulatory failure was therefore a double-edged sword. Groups that experienced a more extreme form of failure (regulatory abuse) had vital social, behavioral, and institutional information available to them, in order to devise wiser, more effective enforcement systems. However, they were only able to do so when they were guided by principles of restorative justice. Without this guidance, the groups tended to learned maladaptive lessons (misconceptions) from their prior failure—perpetuating prior abuses and/or inspiring continued regulatory failures, such as lax enforcement and abandonment. In contrast, facilitated groups (especially PFN) reported greater institutional responsiveness to fix perceived shortcomings of prior enforcement, enhance legitimacy, and ensure reconciliation and restitution (i.e., atonement, forgiveness). For PFN groups in particular, facilitation prompted the group members to reflect on wise and unwise use of penalties, encouraging conceptual development. These findings, though preliminary, mirror similar patterns observed in real-world dilemmas [29, 30].

This achievement is consistent with observations made about discovery-based learning in STEM education. Naïve students appear to learn complex STEM concepts better after an initial phase of exploration, prior to direct conceptual instruction [43, 47, 72]. The achievement also demonstrates individuals’ latent capacity for learning and self-governance, which has been previously demonstrated in prior lab experiments and field studies using naïve participants, such as university students and individuals situated in real-world dilemmas [6, 17–19]. Importantly for both science and society, this achievement exemplifies the neglected Ostrom concept of human governance as an act of “societal problem-solving” and civic learning.

No matter the structure, enforcement systems are created and implemented by fallible human beings in order to resolve complex societal dilemmas [14, 21]. Enforcement systems are bound to backfire when individuals—policymakers, enforcers, and enforced—do not understand how to design or use them wisely [8, 23]. This includes students, who are members of society and civic agents responsible for democratic institutions, such as enforcement. The current experiment, and prior field research it is designed to emulate [see 17, 52, 54 for review], suggests that people cannot fully comprehend underlying principles of good institutional design (e.g., enforcement) without direct experience and the right learning conditions [23, 40].

This observation has important implications for civic education. Learners may need to explore social-ecological dilemmas and practice principles of enforcement themselves to fully comprehend their meaning and application. Such exploratory learning seems to more fully engage innate trial-and-error (i.e., Bayesian) social-learning processes [54, 73]. STEM education research suggests that exploratory learning improves conceptual understanding by making core problem features more salient, raising awareness of the pros and cons of potential solution strategies, heightening awareness of one’s own knowledge gaps, and increasing motivation to learn [42–45, 47, 74, 75]. Educators and practitioners are increasingly using simulated dilemmas (e.g., serious social-ecological dilemma games), like the one we used in this experiment, to teach students and dilemma stakeholders about the complex features and processes of social-ecological dilemmas [76, 77]. This approach has so far proven to be beneficial [e.g., 54].

However, to our knowledge, we are the first to use gamification in a social-ecological dilemma setting to teach design principles of human governance. Our study indicates that gamification may enable naive groups to gain experience in self-governance and institutional design, informing their conceptual development. Unfortunately, civic education typically lacks this practice [21], arguably stymying human progress [54, 76]. The current study hints to an additional possibility. It may be possible to use such social dilemma games and exploratory learning to facilitate conceptual understanding of other Ostrom design principles (e.g., shared decision-making, equity).

## Limitations and future directions

We observed promising information about human learning and enforcement in this experiment. However, our findings are limited to a controlled experimental setting with an undergraduate student population from a particular US university. A great deal of knowledge about self-governance, including Elinor Ostrom’s own understanding, comes from similar experimental paradigms using university students as naïve citizen-participants [e.g., 6, see 17, 18, 78 for review]. However, university students represent a particular type of citizen or civic agent, with potentially different knowledge and experience from other kinds of citizens and learners in society [79, 80]. These differences could influence their conceptual understanding and preferences for enforcement, compared to broader society [24, 81].

Furthermore, we focused on democratic governance and enforcement systems in a particular national and cultural context. Different nations and subcultures have different overarching governance systems, norms, and histories regarding the “wise” or “unwise’ use of enforcement systems [e.g., 19, 67]. These backgrounds likely influence their preferences for enforcement systems and may influence their learning processes and trajectories in ways not anticipated by the current study [for broader discussion, see 8–10, 23]. It is therefore important to replicate and extend this work to other populations and scenarios, including actual social-ecological dilemmas involving real stakeholders. For example, Vollan (2008) observed that different villages occupying areas of South Africa with different historic experiences with regulatory failure and abuse, and therefore trust, responded differently to different forms of enforcement (i.e., monetary sanctions versus rewards). Batrance et al. (2022) observed that university students were more likely to comply with tax enforcement systems when they perceived a strong government authority, whereas business entrepreneurs (with lived experience dealing with taxes and tax enforcement) responded more positively to the perceived trust and legitimacy of the enforcement system and its enforcers [see also, 82]. However, to our knowledge, these and most other studies of enforcement did not examine individuals’ conceptual understanding of enforcement, which is critical for future research, if the results of the current study are to be generalized for behavioral theory and application.

### Policy implications

Human capacity for self-governance and enforcement—specifically, constitutional decision-making and institutional creation—deserves greater scientific and public policy attention [14, 21]. Contemporary enforcement experiments typically examine preferences for enforcement systems in highly constrained, predefined choice experiments that do not give participants autonomy to discuss or autonomously create enforcement systems [2, 22, 83–85]. This practice has led many scientists to believe the general population is incapable of governing themselves or creating wise enforcement systems [7, 21]. Vincent and Elinor Ostrom suggest that this practice is not only limiting to scientific inquiry, but is also a reflection of misconceptions about the democratic basis for enforcement in modern democracies [6, 7, 14].

It is important to disabuse these misconceptions from science [7, 21]. Though complex, imperfect, and somewhat difficult to recognize, enforcement systems in democratically governed societies are fundamentally manifestations of citizen-driven (i.e., self-governing) and co-productive (i.e., government-citizen collective action) processes [86, 87]. Citizens elect officials, which decide rules governing society and the enforcement systems that hold citizens accountable. Citizens also become the police officers, jurors, judges, lawyers, and other “enforcers” that implement and maintain these systems. Finally, citizens ultimately approve or disapprove the rules and regulatory systems governing them, and society’s social-ecological systems, stabilizing or destabilizing particular regulatory regimes by popular will [2, 23, 25, 32, 34]. If civic agents play a variety of roles in democratic enforcement systems [81], then each role should conceivably receive scientific attention and figure prominently in civic education. This recommendation is especially pertinent because studies consistently demonstrate that individuals respond to and accept enforcement systems qualitatively differently when externally imposed versus end ogenously created [9, 12].

The current results add to the larger philosophical discussion, which suggests that regulatory policies that limit citizen participation in the conceptualization, design, implementation, and reform of the regulatory systems that govern them, deny civic agents valuable experience needed to learn from prior regulatory failures to develop wiser, more effective systems [23, 53, 88]. Few empirical case studies and even fewer experiments engage this critique [12, 88] or attempt to understand how people conceptualize and learn from regulatory failures [6, 10]. We have attempted to correct this deficit with an initial experiment.

## Conclusion

The current study demonstrates that naïve individuals in a simulated social-ecological dilemma are capable of developing wiser rule enforcement to enhance both social and ecological sustainability. We identify two potentially important factors for helping individuals achieve this learning. First, individuals may benefit from prior failure with an enforcement system. Without failure, they may not identify the important reasons enforcement is needed, or understand how to use enforcement constructively. Second, individuals may benefit from guidance that helps them discuss and deal with the harms and benefits of enforcement in a productive manner. We found Ostrom’s design principle for restorative justice to beneficial for this guidance.

## Supporting information

S1 Appendix (DOCX)

S1 Protocol (DOC)

S1 File (PDF)
