# Supplementary material for: Learning from regulatory failure: How Ostrom’s restorative justice design principle helps naïve groups create wiser enforcement systems to overcome the tragedy of the commons
Source: PLoS One. 2024 Aug 23;19(8):e0307832. doi: 10.1371/journal.pone.0307832 (PMC11343373; doi:10.1371/journal.pone.0307832)
Supplement: S1 Appendix — (DOCX) [file pone.0307832.s001.docx]

**SUPPLEMENT**

**CONTENTS**

[1.0 RESOURCE DILEMMA AND SOCIAL OPTIMUM 2](#_Toc135231375)

[1.1 Resource Pool 2](#_Toc135231376)

[1.2 Resource Characteristics 2](#_Toc135231377)

[1.3 Social Optimum 3](#_Toc135231378)

[2.0 EXPERIMENT MATERIALS 4](#_Toc135231379)

[2.1 Experiment Instructions 4](#_Toc135231380)

[2.2 Psychological Measures (Surveys) 13](#_Toc135231381)

[2.2.1 Survey 1 13](#_Toc135231382)

[2.2.2 Survey 2 20](#_Toc135231383)

[3.0 CODING METHODOLOGY 21](#_Toc135231384)

[3.1 Conservation Agreements 21](#_Toc135231385)

[3.2 Enforcement Systems (ES) 22](#_Toc135231386)

[3.3 Conceptual Understanding of Enforcement (CUE) 22](#_Toc135231387)

[3.4 Democratic Decision-Making (DDMI) 23](#_Toc135231388)

[4.0 ADDITIONAL ANALYSES 24](#_Toc135231389)

[4.1 Perceptions and Motivations 24](#_Toc135231390)

[4.1.1 Perceptions of the Dilemma 24](#_Toc135231391)

[4.1.2 Need Satisfaction 25](#_Toc135231392)

[4.1.3 Cooperative Motivations 26](#_Toc135231393)

[4.1.4 Group Cohesion: Self-Other Merging and Trust 29](#_Toc135231394)

[4.2 Conservation Strategies 29](#_Toc135231395)

**Datasets archived with this manuscript:**

- **Dataset 1.** Raw Decision Data. Provides the raw decision data (i.e., number of tokens collected by each individual) in the common-pool resource dilemma experiment.
- **Dataset 2.** Chat and Codes. Provides the group communication (chat text) with the content and functional codes that were applied (e.g., decision events, conservation agreements, democratic decision-maing index, enforcement system).
- **Dataset 3.** Main Dataset. Final organized dataset including individual survey responses and group aggregated decision data and computed indicators.

# 1.0 RESOURCE DILEMMA AND SOCIAL OPTIMUM

In this section, we present the key characteristics of the resource dilemma and results of an optimization analysis to determine the social optimum of performance in the dilemma under different starting conditions and constraints [for additional details regarding the foraging task and software, 1].

## 1.1 Resource Pool

Our experiment created a real-time, renewable common-pool resource (CPR) environment. Group members see one another, and all of their actions (e.g., harvests, sanctions), on screen in real-time. They can collect tokens worth $0.02 each by moving their avatar over a token and pressing spacebar. The CPR itself was represented by a 26 × 26 grid of cells, and the tokens occupy cells within the grid. Participants’ payment was determined by how many tokens they harvested during the experiment, plus a $3 show-up fee and earnings from a quiz (up to $0.50) at the beginning of the experiment that tested participants’ knowledge of the task instructions. Participants’ avatars began each round lined up horizontally in the middle row (13^th^ cell) of the screen. The avatars and tokens were displayed on the screen for each group member to see. Therefore, group members (a) had complete information on the spatial location of the tokens (i.e., CPR), (b) knew how many tokens had been collected by each group member, and (c) could clearly see each person’s movements and harvesting actions. This information was displayed in real-time; a timer counting down how much time was left in each round was also displayed.

**
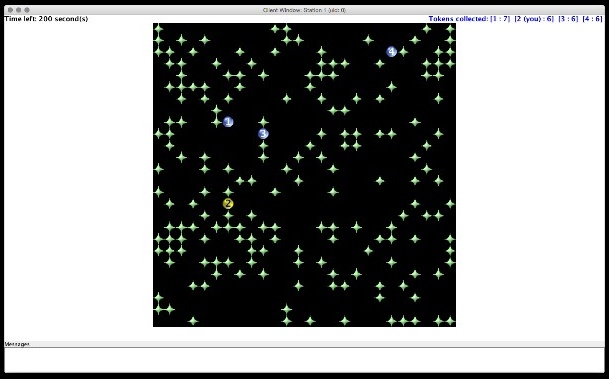
**

**Foraging Task.** Star-shaped tokens are resource units (“plants”). Circles are participant avatars. Participants see their avatar in yellow. Tokens collected by each person are displayed in the upper right-hand corner each round (e.g., Player 2 (6 tokens) is “[2 (you), 6]”).

## 1.2 Resource Characteristics

The design features of this CPR—probabilistic distribution and renewal of tokens, with collapsibility—capture some key characteristics of many spatially dependent renewable resources [2]. At the beginning of each round 25% of the 26 × 26 CPR grid was occupied by tokens (for a total of 169 tokens); the location of these tokens was randomly generated. The CPR is renewable because CPR tokens can regenerate (or grow). The probability that any particular empty cell on the CPR grid generates a new token is dependent on the number of adjacent cells that are still occupied by a tokens. Thus, regrowth is density dependent. Specifically, the probability *p_t_* is linearly related to the number of neighboring tokens: *p_t_* = *p*n_t_*/N, where *n_t_* is the number of neighboring cells containing a token, and N is the number of neighboring cells (N = 8, because we use a Moore neighborhood with a range of 1, and *p* = 0.01). Therefore, empty cells that are completely surrounded (by eight tokens) regenerate with higher probability than empty cells with fewer abutting tokens. The CPR is finite because there must be at least one CPR token on the screen (i.e., adjacent to an empty cell) for that cell to regenerate a new token. When participants harvest every token on the grid, they exhaust the resource, and it will produce no additional tokens for that round. Groups that harvest all of the tokens on the screen must wait for time to expire before continuing to the next round. These conditions reset at the beginning of each round, providing participants with a new CPR environment to manage.

## 1.3 Social Optimum

In our experiment, the optimum level of harvests to generate the greatest individual earnings for all players, while perpetuating the CPR, depends on the initial starting conditions, the rate and locations at which individuals harvest tokens, and the subsequent rate of regrowth. However, if we ignore spatial variability (essentially averaging across initial states), then the optimal harvesting strategy is to wait two minutes before harvesting to let the CPR cover 50% of the CPR grid. After two minutes, participants should use selective harvesting in a checkerboard pattern to maintain this same level of coverage until the last 30 seconds of the round; at that time, they can harvest as tokens as quickly as possible (to yield an estimated 548 total tokens overall) (Figure A.2). If participants do this, and they each get an equal share of the CPR, then each person can harvest about 137 tokens ($2.74) each round, for a total of 1233 tokens, or $24.66 (plus $3 show-up fee and quiz earnings up to $0.50), over the course of the experiment (for an overall total of $28.16).

**Social Optimum.** Optimal harvest strategy and resource level in an idealized spatial configuration of the tokens.

Researchers have demonstrated that similar estimates, which instead calculate the average harvests from simulations that incorporate spatial variability of token distributions and their renewal, closely approximated the simplified situation we just described [2,3]. Hence, 548 tokens can be used as a benchmark for the social optimum in a round. Here are the estimated Nash and social optimum (i.e., 548 tokens) for common conservation strategies in our experiment when there is perfect cooperation [cf. 4]. Group level estimates for different strategies are:

- Default (no rule): Nash 177 tokens
- 60 Second Rule (wait 60 seconds before harvesting any tokens): Nash 274 tokens
- Private Property Rule: Nash 548 tokens
- Optimal Strategy (wait 120 seconds to harvest; harvest in checkerboard pattern until last 30 seconds; then collect as quickly as possible). Nash 1233 tokens

# 2.0 EXPERIMENT MATERIALS

In this section, we provide the experiment task instructions shown to participants. We also show and annotate (i.e., label and provide the citations for) the psychological measures used in each survey. Our experimental protocol and software is archived publicly online at [Zenodo](https://doi.org/10.5281/zenodo.5165359).

## 2.1 Experiment Instructions

[*INTRODUCTORY TASK INSTRUCTIONS*]

*These are the introductory task instructions shown to participants at the beginning of the experiment.*

**Welcome** [*Screen 1*]

Welcome to the experiment. The experiment will begin shortly.

Please **wait quietly**, and **do not close this window, open any other applications, or communicate with any of the other participants**.

**General Instructions** [*Screen 2*]

**Your Earnings**

You have already earned $3 by showing up at this experiment. You can earn more, up to a maximum of about $10-$30, by participating in this experiment, which will take about an hour and a half (90 minutes) to two hours. The amount of money you earn depends on your decisions, as well as the decisions of your group members during the nine rounds of the experiment.

**The Token Task**

Today's experiment is on the computer. In the experiment, you can collect green, diamond-shaped tokens
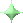
, and you will earn **$0.02** for each token you collect. When the token task begins, you will appear on the screen as a yellow dot
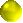
. To collect a token, move your yellow dot over a green token and **press the space bar.** If you move over a token without pressing the space bar you will NOT collect that token. You can move up, down, left, or right by pressing the four arrow keys (up, down, left, or right) on your keyboard. Or, you can use the W, S, A, D keys (or I, K, J, L).

Later, you will be assigned to a group. The other group members will appear as blue dots
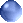
.

**Tokens**

The tokens that you collect have the potential to regenerate. After you have collected a green token, a new token can re-appear on that empty space. The rate at which new tokens appear depends on the number of tokens that are nearby, on adjacent spaces. The more tokens there are in the eight spaces that surround an empty space, the faster a new token will appear to fill the empty space.

For example, take a look at Image 1 and Image 2 below. The empty space in Image 1 (marked with X) will regenerate faster than the empty space in Image 2, because it is surrounded by more tokens.

Finally, tokens will not regenerate in an empty space if it is surrounded by empty spaces on all sides, as shown in Image 3. At least one token is needed for new tokens to appear.


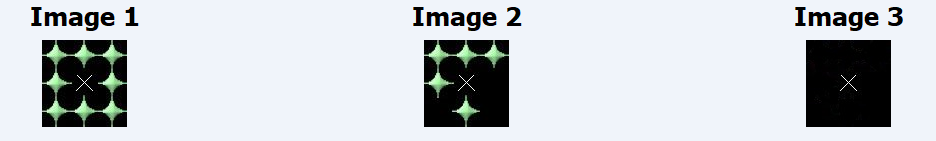


If you have any questions at this time, please raise your hand and someone will come over to your station and answer it. If you have no questions, continue to the quiz below.

**Quiz**

In a moment, you will do a practice round of the token task. Before we go to the practice round, answer the following questions to make sure you understand the instructions. You will receive $0.10 for each correct answer. Click the “submit” button at the bottom of the screen, when you are ready to submit your answers.

**Q1. Once you have collected all the tokens on the screen, no new tokens will appear.**

Correct

Incorrect

**Q2. Tokens regenerate faster (new tokens appear faster) when empty spots are surrounded by more tokens.**

Correct

Incorrect

**Q3. Which of the following sequences is not possible, A, B, or C?**

*A*

*B*

*C*

**Q4. Each token you collect is worth? (select the correct value)**

**
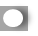
**$.01

**
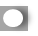
**$.02

**
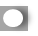
**$.03

**
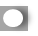
**$.04

**
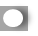
**$.05

**Q5. The amount of money you can earn today only depends on the decisions you make?**

**Quiz Results** [*Screen 3*]

*Immediately after the quiz, the computer showed each participant their quiz feedback. This feedback included correct answer to each question. In this example, the participant got only Question 2 correct.*

You answered 1 out of 5 questions correctly and earned **$0.10.** The questions you answered incorrectly are highlighted in red below. The correct answer to each question is also shown below.

When you have finished reviewing your responses, click the “Continue” button below to continue.

**Q1. Once you have collected all the tokens on the screen, no new tokens will appear.**

Not correct. New tokens only appear if there is at least one token on the screen.

**Q2. Tokens regenerate faster (new tokens appear faster) when empty spots are surrounded by more tokens.**

Correct. Tokens regenerate faster when empty spots are surrounded by more tokens.

**Q3. Which of the following sequences is not possible, A, B, or C?**

Not correct. Sequence B is not possible, because new tokens will not appear once all the tokens are gone.


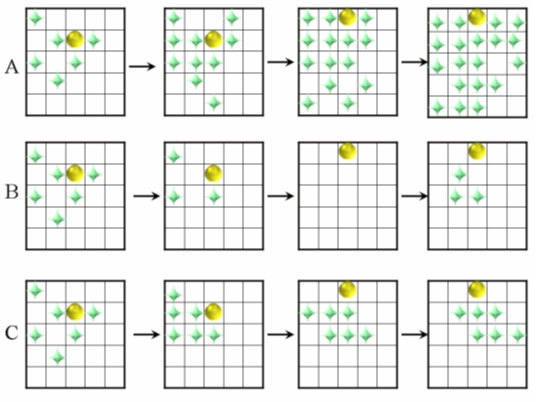


**Q4. Each token you collect is worth? (select the correct value)**

**Not correct. Each token you collect will be worth $0.02. For example, if you collect 10 tokens you will earn $0.20 (twenty cents). If you collect 50 tokens you will earn $1.00 (one dollar), and if you collect 100 tokens or more, you would earn $2.00 or more.**

**Q5. The amount of money you can earn today only depends on the decisions you make?**

Not correct. The amount of money you earn today will depend on the decisions you make and the decisions that other people in your group make. Each person’s decisions to collect tokens affects the number of tokens (money) available for the other people in group.

Continue

**Practice Round Instructions** [*Screen 4*]

Once everyone has finished the quiz, we will start a practice round of the token task.

During the practice round, you will have four minutes (240 seconds) to practice with the experimental environment. The decisions you make in this round will NOT influence your earnings. The practice screen consists of an area that is 13 × 13 spaces. 25% of the spaces will be occupied by green tokens at the beginning of the practice round.

During this practice round, and **only during** this practice round, you will be able to reset the tokens displayed on the screen. When you push the **R** key you will reset the distribution of the tokens to randomly occupying 25% of the cells with green tokens again.

**Do you have any questions?** If you have any questions at this time, please raise your hand and someone will come over to your station and answer it.

**Round 1 Instructions** [*Screen 5*]

**Please read the following instructions carefully.**

**Groups**

In this round the renewable resource will become four times bigger. You will share this larger environment with three other random players in this room. In particular, **each participant in this room has been randomly assigned to one of several equal-sized 4 person groups**. And everyone in your group has been randomly assigned a number from 1 to 4. **You will stay in the same group for the entire experiment**, and each person's number from 1 to 4 will remain the same throughout the experiment. The other members of your group will appear on the screen as blue dots
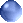
 with a white number embedded in the dot.

In each round of the token task, you can see how many tokens each player has collected at the top right corner of the screen. On the top left corner of the screen, you will see the remaining time in the round.

**Anonymity**

Because group membership was randomly assigned by the computer, neither you nor the experimenter will be able to identify which person in the room has been assigned to a particular group or number within a group. Your anonymity is guaranteed.

**Tokens**

Each group has its own set of token resources.

**Do you have any questions so far?** If you have any questions at this time, raise your hand and someone will come over to your station and answer it.

**[*TREATMENT INSTRUCTIONS*]**

*From this point forward, the instructions differed by experimental treatment. There were six treatments created with different combinations of these factors: order (when treatments were introduced during the three phases of the experiment), communication (C), facilitated communication (F), monetary penalties (P), nothing (N, no treatment): CNN, FNN, NCN, NFN, PCN, PFN. Note that all treatments could use monetary penalties during communication phases.*

*We show the key instructions for communication, facilitation, penalties, and nothing. Treatments were created by presenting the relevant instruction elements in the proper order.*

*To demonstrate key differences, we show the instructions for CNN, FNN, and PCN.*

**[*TREATMENT 1: CNN*]**

*Phases: 1 (Communication with monetary penalties), 2 (Nothing), 3 (Nothing)*

**Group Communication** [*Phase 1: Screen 1*]

**From this point forward, you will be able to communicate with the members of your group for 6 minutes (360 seconds) before each round.** Use this time to consider potential strategies to manage the tokens. You will also be able to communicate during each round. You will also be able to communicate **during each round.**

**In a moment, you will see a Group Communication window.** If you want to communicate with the people in your group, type your message in the line labeled "Chat" (located at the top left of the screen), and then press Enter.

**Basic Guidelines for Communication**

You can discuss anything you want. And, you are free to communicate as much or little as you want. However, please do not (1) discuss side payments that would result in exchange of money outside the experiment, (2) reveal your identity, or (3) make physical threats to other participants.

**Do you have any questions so far?** If you have any questions at this time, raise your hand and someone will come over to your station and answer it.

**Monetary Penalties** [*Phase 1: Screen 2*]

**Applying fines to other players**

In addition to communication, you will now have the option to apply monetary penalties to other participants. When you penalize someone, it will reduce their earnings by 2 tokens (4 cents) and cost you 1 token (2 cents). You can do this as much as you want as long as you have at least 1 token, and the other person has some tokens to be reduced.

Each person in your group has a number from 1 to 4. If you press a number key 1-4, you will give a monetary penalty to that person, reducing the number of tokens that person has collected during the round by 2. It will also reduce your own token amount by 1. The decision whether or when to use this option is up to you.

- When you give a monetary penalty to another person, they will receive a message stating that you have reduced their tokens. Likewise, if another person gives you a monetary penalty, you will also receive a message. These messages will be displayed on the right side of your screen.
- If you are being penalized or you are penalizing another person, you will see some visual cues. When someone else is penalizing you, your avatar will turn red briefly with a blue background. The dot of the person applying a monetary penalty to you will turn purple with a white background.
- You may apply monetary penalties to other participants as long as (1) there are tokens remaining on the screen and (2) both you and the other person have a positive number of tokens collected during the round. **Each time you press the numeric key corresponding to another person, your tokens will be reduced by 1, and their tokens will be reduced by 2.**

**Do you have any questions so far?** If you have any questions at this time, raise your hand and someone will come over to your station and answer it.

[*Chat Screen Instructions*]

*These are the summary instructions displayed on the screen during the 6-minute dedicated communication period that occurred before each round during the Communication phase (i.e., Phase 1 in this treatment). This screen also displayed the time left in chat (timer, top of screen).*

**Group Communication**

You now have 6 minutes (360 seconds) to communicate with the members of your group. Type your message in the line above, and press ENTER. Your messages will appear in the box on the right, which is visible to all your group members. Messages from Player 1, 2, 3, or 4 are labeled, 1, 2, 3, or 4 in the message. Box

**Guidelines**

1. Discuss anything you want.
2. Consider strategies to manage the tokens.

**You may not:**

1. Make side payments that would result in exchange of money outside of the experiment.
2. Reveal your actual identity.
3. Make physical threats.

We are monitoring the chat traffic. If we discover any violation of these particular rules (side payments, physical threats, revealing your identity), we will have to stop the experiment and remove the offending group from the room.

[*Between Round Reminder Instructions*]

*Participants were briefly reminded of the communication and penalty instructions before each round, in addition to seeing the Chat Screen instructions during each chat period in Phase 1.*

Round [1, 2, 3] is about to begin. Like before, it is 4 minutes long (240 seconds). You will be **able to communicate** with your group members during the round by using the Chat window on the right side of the screen. You can send a message quickly by pressing the enter key, typing your message, and then pressing the enter key again. You will also **have the ability to use monetary penalties**. To do so, press the number 1, 2, 3, or 4, of the desired person.

**Please raise your hand if you have any questions.**

**Round 3 Instructions** [*Phase 2*]

*After Phase 1, in this treatment (CNN), players could no longer communicate or use penalties. The following instructions were shown at the beginning of Round 3 (the first round of Phase 2).*

**Round 3 is about to begin. Like before, it is 4 minutes long (360 seconds).**

**Important new instructions!**

**From this point forward, you will NOT be able to communicate with your group members. And, you also will NOT have the option to use monetary penalties.**

**Do you have any questions so far?** If you have any questions at this time, raise your hand and someone will come over to your station and answer it.

*Note: These instructions were repeated (as a reminder) before each round in Phase 2. Similar instructions were used in Phase 3.*

**[*TREATMENT 2: FNN*]**

*Phases: 1 (Facilitated communication with monetary penalties), 2 (Nothing), 3 (Nothing)*

**Additional Guidelines for Communication and Monetary Penalties** [*Phase 1: Screen 3*]

*Groups in the FNN treatment received additional instructions (guidelines) for facilitate communication and use of monetary penalties. These instructions were given in addition to the basic guidelines for communication (Screen 1) and monetary penalties (Screen 2) used in the CNN treatment.*

**Important Instructions!**

The 6 minute (360 seconds) communication period is about to begin. **Please read the following instructions carefully and do your best to follow these guidelines:**

- Consider potential strategies to manage the tokens.
- As a group, consider if you want to use monetary penalties. If your group decides to use monetary penalties, discuss when and how they will be used.
- Be sure to discuss the reasons you want to use particular strategies to manage the tokens, or why you want to use monetary penalties. Strategies and monetary penalties should be justified as a group.
- Have an open discussion. Give each person the opportunity to discuss, and try your best to be open to different viewpoints and ideas.
- Finally, do your best to be polite and respectful when discussing (try not to use overly harsh language or heated arguments).

**Do you have any questions so far?** If you have any questions at this time, raise your hand and someone will come over to your station and answer it.

[*Chat Screen Instructions*]

*These are the summary instructions displayed on the screen during the 6-minute period of facilitated communication that occurred before each round during the facilitated communication phase (i.e., Phase 1 in this treatment). This screen also displayed the time left in chat (timer, top of screen).*

**Group Communication**

You now have 6 minutes (360 seconds) to communicate with the members of your group. Type your message in the line above, and press ENTER. Your messages will appear in the box on the right, which is visible to all your group members. Messages from Player 1, 2, 3, or 4 are labeled, 1, 2, 3, or 4 in the message. Box

**Guidelines**

1. Discuss anything you want.
2. Consider strategies to manage the tokens.
3. Consider if the group wants to use monetary penalties. If so, when and how?
4. Discuss your reasons. Strategies and monetary penalties should be justified as a group.
5. Give each person the opportunity to discuss. Try to be open to different viewpoints.
6. Try to be polite and respectful.

**You may not:**

1. Make side payments that would result in exchange of money outside of the experiment.
2. Reveal your actual identity.
3. Make physical threats.

We are monitoring the chat traffic. If we discover any violation of these particular rules (side payments, physical threats, revealing your identity), we will have to stop the experiment and remove the offending group from the room.

**Communication Instructions for Round (2, 3).** [*Phase 1*]

*These are the additional instructions FNN groups received after Round 1 and 2 during Phase 1.*

The 6 minute (360 seconds) communication period for the next round of the token task is about to begin.

**Please read the following instructions carefully:**

**Discussing Monetary Penalties**

If your group used monetary penalties, please discuss this. Consider whether you want to change how monetary penalties will be used, in order to improve the situation.

Give each person who received monetary penalties the opportunity to discuss, and try your best to address any concerns they mention. The group members that used monetary penalties should also have the opportunity to explain their perspective. For example, someone may be dissatisfied with the group’s strategy for managing the tokens, or have a problem with the monetary penalties. Or they might not understand the group’s strategy.

Whatever the issue, try your best to explain the reasons for your decisions and ideas. And, if possible, try to find a solution that satisfies the group and resolves potential conflicts.

**Finally, discuss anything else you wish.** And, if you have any concerns with the way the group is managing the tokens (such as the strategies and agreements), or the way the group has decided to use monetary penalties, be sure to discuss those issues, too. Try to explain the reasons behind your thoughts. If possible, see if the group can find a solution that satisfies everyone.

**Do you have any questions so far?** If you have any questions at this time, raise your hand and someone will come over to your station and answer it.

**Note: Instructions for Phase 2 and 3 were the same as CNN.*

**[*TREATMENT 6: PFN*]**

*Phases: 1 (Penalties without communication), 2 (Facilitated communication with penalties), 3 (Nothing)*

**Monetary Penalties** [*Phase 1: Screen 1*]

*Groups in this treatment began with the same basic penalty instructions used, for example, in CNN, but did not have the ability to communicate (so no communication instructions were shown).*

**Applying fines to other players**

In addition to communication, you will now have the option to apply monetary penalties to other participants. When you penalize someone, it will reduce their earnings by 2 tokens (4 cents) and cost you 1 token (2 cents). You can do this as much as you want as long as you have at least 1 token, and the other person has some tokens to be reduced.

Each person in your group has a number from 1 to 4. If you press a number key 1-4, you will give a monetary penalty to that person, reducing the number of tokens that person has collected during the round by 2. It will also reduce your own token amount by 1. The decision whether or when to use this option is up to you.

- When you give a monetary penalty to another person, they will receive a message stating that you have reduced their tokens. Likewise, if another person gives you a monetary penalty, you will also receive a message. These messages will be displayed on the right side of your screen.
- If you are being penalized or you are penalizing another person, you will see some visual cues. When someone else is penalizing you, your avatar will turn red briefly with a blue background. The dot of the person applying a monetary penalty to you will turn purple with a white background.
- You may apply monetary penalties to other participants as long as (1) there are tokens remaining on the screen and (2) both you and the other person have a positive number of tokens collected during the round. **Each time you press the numeric key corresponding to another person, your tokens will be reduced by 1, and their tokens will be reduced by 2.**

**Do you have any questions so far?** If you have any questions at this time, raise your hand and someone will come over to your station and answer it.

**Note: During Phase 2, groups in this treatment received the facilitated communication instructions illustrated for FNN. During Phase 3, groups in this treatment received the standard instructions used for “Nothing” when communication and penalties were disabled (e.g. see Phase 2 of CNN).*

## 2.2 Psychological Measures (Surveys)

*The psychological measures used in the surveys of the experiment and their sources are listed here* [cf. 4]*. We also report the internal consistency scores (Cronbach α) for each subscale. Section instructions were shown on their own screen. Subscales were presented in the order shown. Individual items were presented one-by-one in randomized order, to counteract order effects. Unless noted otherwise, participants responded to all items on a 7-point response scale, ranging from 1 (strongly disagree) to 7 (strongly agree). Some additional, unvalidated items were administered as exploratory measures but performed poorly psychometrically; preliminary analyses revealed that these items neither add nor subtract (alter) from reported patterns or conclusions. Therefore, we report the validated measures. We did not show survey titles, section titles, subscale names, or item names to participants. Survey 3 measured standard demographic information (e.g., gender, age, race/ethnicity, education, household income) and is therefore not shown.*

### 2.2.1 Survey 1

*Administered immediately after the treatment’s communication phase. CNN and FNN, Survey 1 occurred after Phase 1. NCN, NFN, PCN, and PFN, Survey 1 occurred after Phase 2.*

**[SECTION 1: Conservation Agreements]**

**Did Your Group Create Some Rules and Agreements to Manage The Tokens?**

When groups communicate in a situation like the token task, they sometimes create some rules or agreements to manage the tokens together. Next, we would like to ask you some questions about the rules and agreements your group made, if any.

*We are interested in your honest opinion. There are no right or wrong answers. Your responses are anonymous. Other participants will not know your responses, and the experimenter cannot trace them specifically back to you.*

***[Question:]***

Please describe and explain any strategies and agreements your group made to manage the tokens.

Please describe the strategies/agreements even if some of the people in your group did not obey them very well.

*If your group did NOT create any strategies or agreements, please type "None".

*Click the box below to type your response. Please be sure to describe and explain your group's rules/agreements well enough for us to understand.*

**[SECTION 2: Acceptance of the Agreement]**

**How Do You Feel About Your Group’s Strategies and Agreements?**

Next, we would like to ask you how you feel about the strategies or agreements your group decided to use to manage the tokens.

**Your responses will not be shared with the group**, and will remain confidential. Please remember that there are no right or wrong answers. We sincerely want to learn your opinion about the situation.

**Acceptance (α = .90)**

*Item prompt:* Please indicate how much you agree or disagree with the statement below at this moment.

1. I approve of the strategies/agreements my group made to manage the tokens.
2. I am satisfied with the strategies/agreements my group made to manage the tokens.
3. I support the strategies/agreements my group made to manage the tokens.

Colquitt (2001): “The grade I am currently receiving in this course is acceptable.” “I am satisfied with my current grade in the course.” (Outcome satisfaction items, p. 391)

**Rule Effectiveness**

Under ideal conditions (if everyone followed them perfectly), how effective do you think your group’s rules/agreements would be for managing the collection of tokens?

*Response scale:*  Very Ineffective (1) to Very Effective (7)

**[SECTION 3: Motivations]**

**Why Did You Obey Your Group's Strategies/Agreements the Way You Did?**

People may follow or obey a group’s strategies/agreements to different extents and for different reasons. We would like to ask you about your reasons for obeying the group’s rules/agreements the way you did.

*Your responses will not be shared with the group, and will remain confidential. Please remember that there are no right or wrong answers. We sincerely want to learn your opinion about the situation.*

**Economic Sanctions (Fear Penalties) (α = .76)**

*Item prompt:* The reason I followed my group's strategies/agreements, the way I did, was because...

1. I did not want to be punished with monetary penalties for disobeying the strategies/agreements.
2. I thought I would be penalized if I did not follow the strategies/agreements.

**Social Sanctions (α = .76)**

1. I did not want to be criticized for disobeying the strategies/agreements.
2. I thought the others would "yell" at me (or shame me), if I did not follow the strategies/agreements.

**Social Pressure (α = .76)**

1. I thought my group members would disapprove of my behavior, if I did not obey the strategies/agreements.
2. I did not want to disappoint the other people in my group, by disobeying the strategies/agreements.

**Guilt (α = .89)**

1. I did not want to feel guilty for disobeying my group’s strategies/agreements.
2. I did not want to feel bad about myself for disobeying my group’s strategies/agreements.

**Anticipated Earnings (α = .93)**

1. I felt that the strategies/agreements would help me get more tokens (more money).
2. I felt that the strategies/agreements would increase the number of tokens (amount of money) I could get.

**Internalized (α = .71)**

1. I really believed these were important strategies/agreements to have.
2. I felt that they matched with my values and desires.

**Security (α = .77)**

1. I felt that the strategies/agreement would keep me safer from uncertainties.
2. I felt that strategies/agreement would make me feel more secure.

**Note: we asked these same questions targeting perceptions of the group members (i.e., why do you think the other members of your group members followed the rules the way they did?). Responses for oneself and others were highly similar, yielding the same overall patterns.*

Sources this section:

DeCaro, D.A., Janssen, M.A., Lee, A. (2015). Synergistic effects of voting and enforcement on internalized motivation to cooperate in a resource dilemma, *Judgment and Decision Making*, 10(6) 511-537.

Sheldon, K. M., & Elliot, A. J. (1998). Not all personal goals are personal: comparing autonomous and controlled reasons for goals as predictors of effort and attainment. *Personality and Social Psychology Bulletin, 24,* 546–557.

Soenens, B., Vansteenkiste, M., & Niemec, C. P. (2009). Should parental prohibition of adolescents’ peer relationships be prohibited? *Personal Relationships, 16,* 507–530.

**[SECTION 4: Perceptions of Communication]**

**Your Perceptions of the Communication**

For these next questions, we would like to understand how you felt about the communication you had with the members of your group.

***Your responses will not be shared with the group****, and will remain confidential. Please remember that there are no right or wrong answers. We sincerely want to learn your opinion about the situation.*

**Procedural Fairness (α = .90)**

*Item prompt:* Indicate how much you agree or disagree with the statement below.

1. I felt like the people in my group encouraged open communication and welcomed my input. [*procedural*]
2. I felt like the people in my group acted respectfully when they communicated with me. [*interpersonal*]
3. I felt like the way my group communicated with each other was fair. [*general*]

DeCaro, D.A., Janssen, M.A., & Lee, A. (2021). Motivational foundations of communication, voluntary cooperation, and self-governance in a common-pool resource dilemma. *Current Research in Ecological and Social Psychology*, 2:100016.

Colquitt, J. A. (2001). On the dimensionality of organizational justice: a construct validation of a measure. *Journal of Applied Psychology, 86*(3), 386–400.

van Prooijen, J. W. (2009). Procedural justice as autonomy regulation. *Journal of Personality and Social Psychology*, *96*(6), 1166-1180.

**[SECTION 5: Perceptions of Group Decision Making]**

**Group Decision Making**

Next, we would like to ask some questions about the way your group made important decisions together, about how to manage the tokens.

***Your responses will not be shared with the group****, and will remain confidential. Please remember that there are no right or wrong answers. We sincerely want to learn your opinion about the situation.*

**Procedural Justice and Self-Determination (α = .85)**

*Item prompt:* The way my group made decisions about what to do in the token task made me feel…

1. as if I was able to influence important decisions [*procedural justice*].
2. as if I could determine what I could do [*self-determination: internal perceived locus of control*].
3. as if I had some choice about what to do [*self-determination: choice*]
4. free to do things that agree with my true interests and values [*self-determination: self-concord*].

DeCaro, D.A., Janssen, M.A., & Lee, A. (2021). Motivational foundations of communication, voluntary cooperation, and self-governance in a common-pool resource dilemma. *Current Research in Ecological and Social Psychology*, 2:100016.

Colquitt, J. A. (2001). On the dimensionality of organizational justice: a construct validation of a measure. *Journal of Applied Psychology, 86*(3), 386–400.

Levenson, H. (1980). Differentiating among internality, powerful others, and chance. In H. M. Lefcourt (Ed.), *Research with the Locus of Control Construct* (Vol. 1., pp. 15-63). New York, NY: Academic Press, Inc

Ryan, R. M. (1982). Control and information in the intrapersonal sphere: An extension of cognitive evaluation theory. *Journal of Personality and Social Psychology, 43,* 450-461.

Sheldon, K. M., Elliot, A. J., Kim, Y., & Kasser, T. (2001). What is satisfying about satisfying events? Testing 10 candidate psychological needs. *Journal of Personality and Social Psychology, 80(2)*, 325-339.

**[SECTION 6: Monetary Penalties]**

**How did your group use monetary penalties?**

**For these next questions, we would like to understand how your group used monetary penalties.**

[*Question:*]

Please describe and explain how your group used monetary penalties. When and how were they used? Why were they used?

If your group decided not to use monetary penalties please explain why.

*Click the box below to type your response. Please be sure to describe and explain your group's rules/agreements well enough for us to understand.*

**[SECTION 7: Perceptions of Penalties]**

**How Did You Feel About The Way Your Group Used Monetary Penalties?**

​​
Some groups may decide not to use monetary penalties. Other groups may decide to use them in particular ways. For these next questions, we would like to ask how your group's decision to use monetary penalties, *the way it did,*made you feel.  

Thus, if your group decided not to monetary penalties, how did that make you feel? Or if your group used monetary penalties in particular ways, how did that make you feel? (Answer based on what matches your group's situation the best.)

*Again, there are no right or wrong answers. We are interested in your honest opinion.*

**[*Questions*:]**

**Acceptance of How Group Used Penalties**

1. I approve of how my group used monetary penalties.
2. I am satisfied with how my group used monetary penalties.

*Note: preliminary analyses revealed that most treatments highly accepted the way their group used penalties. However, PCN reported generally lower acceptance.*

**Legitimacy**

*Item prompt:* The way my group used monetary penalties…

1. felt fair.
2. felt justified and legitimate.

*Note: these 2 items are Legitimization items of Restorative Justice (see Section 10 of Survey)*

**Benefits**

1. felt beneficial.
2. helped me reach my goals.
3. helped the group reach its goals.

*Note: preliminary analyses revealed that most treatments perceived moderate/high benefits to how their group used monetary penalties. However, PCN reported lower perceived benefits overall, especially compared to FNN, NFN, and PFN.*

**Coercion/Oppression**

1. felt oppressive
2. felt excessive and coercive

*Note: preliminary analyses revealed no reliable/consistent differences among treatments for perceive coercion/oppression.*

**[SECTION 8: Personal Use of Monetary Penalties]**

**Next, please describe and explain how you personally used monetary penalties.** When and why did you use them?

If you decided not to use monetary penalties please explain why.

*Click the box below to type your response. Please be sure to describe and explain your thoughts well enough for us to understand.*

**[SECTION 9: Reasons for Using Penalties]**

**What Were Your Reasons for Using Monetary Penalties the Way You Did?**

Individuals may use monetary penalties in different ways. Some individuals may not use monetary penalties at all. Others may choose to use them only in particular situations.

These next questions ask about your reason(s) for using monetary penalties the way you did.

[*Questions:*]

Item prompt: The reason I used monetary penalties, *the way I did,* was because...

Necessity

1. I felt that I did not need to use them.
2. I saw no reason to use them.
3. I did not think I would be able to justify using them.

Prevent, Punish, Gain Control (PPC)

1. I wanted to prevent individuals that might disobey the group's strategies/agreements from doing so. [prevent]
2. I wanted to punish or penalize group members that disobeyed the group's token management strategies/agreements. [punish]
3. I wanted to gain more control over the situation [control]

Revenge

1. I wanted to get revenge on someone in the group. [revenge]
2. I was worried that others would seek revenge on me, if I used monetary penalties on them. [*fear revenge*]

Janssen, M.A., Holahan, R., Lee, A. and Ostrom, E., 2010. Lab experiments for the study of social-ecological systems. *Science*, *328*(5978), 613-617.

Other (exploratory)

1. I did not think they would actually stop people from disobeying the group's token management strategies/agreements. [ineffective]
2. It was too costly (cost too much money) to use them. [cost]
3. It took too much time to use them. [time]
4. I wanted to protect our group’s token management strategies/agreements. [protect]
5. I wanted to make sure that the tokens were being collected fairly, and divided fairly, among the members of the group. [equity]

**[SECTION 10: Restorative Justice]**

**Group Discussion of Monetary Penalties**

Next, we would like to ask some questions about how your group discussed monetary penalties.

[*Questions:*]

**Responsiveness**

1. The group did a good job of discussing whether we wanted to use monetary penalties, and the reasons why.
2. The group did a good job of discussing how we wanted to use monetary penalties.
3. If someone had a problem or concern with the way we were using monetary penalties, the group did a good job of discussing how to fix it.

**Restitution**

1. The group did a good job of giving the person *who was penalized* an opportunity to discuss the situation and explain their point of view.
2. The person that used the monetary penalty did a good job of explaining (justifying) why they used it.

**Legitimization**

*Item prompt:* The way my group used monetary penalties…

1. felt fair
2. felt justified and legitimate

**[SECTION 11: Perceptions of the Dilemma]**

**Your Current Thoughts about the Token Task Situation**

We would like to ask about your current perceptions of the Token Task Situation: this includes your feelings and beliefs about your group's behavior and how the token task is going.

*We are interested in your honest opinion. There are no right or wrong answers. Your responses are anonymous. Other participants will not know your responses, and the experimenter cannot trace them specifically back to you.*

**[Perceptions of Group]**

**Questions about Your Group**

First, we would like to ask you some questions about your perceptions of the other people in your group.

***Your responses will NOT be shared with the group****, and will remain confidential. Please remember that there are no right or wrong answers. We sincerely want to understand your opinion about the situation.*

**Self-Other Merging and Trust (α = .95)**

1. Below you see seven pairs of circles. One circle represents yourself, and the other circle represents the other members of your group. At this moment, which pair of circles best reflects how you feel towards the other people in your group?


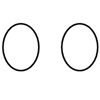

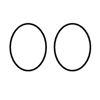

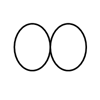

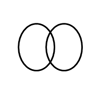

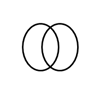

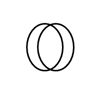

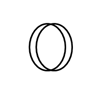


*Item prompt:* Indicate how much you agree or disagree with the statement below at this moment.

1. I feel positively towards the other people in my group.
2. I feel like I can trust the people in my group *NOT* to take advantage of me in the token task right now.

Cameron, J. E. (2004). A three-factor model of social identity. *Self and Identity, 3,* 239-262.

Chen, X. P., Pillutla, M. M., & Yao, X. (2009). Unintended consequences of cooperation inducing and maintaining mechanisms in public goods dilemmas: sanctions and moral appeals. *Group Processes & Intergroup Relations, 12*(2), 241–255.

Karremans, J. (2002). *Forgiveness: Examining its consequences.* Doctoral dissertation, Free University Amsterdam, the Netherlands.

**[Perceptions of Dilemma/Fundamental Needs]**

*Item prompt:* Indicate how much you agree or disagree with the statement below at this moment.

Dilemma

1. The token task situation feels disorganized and chaotic right now. [chaos]
2. I feel like the tokens are being managed well right now. [resource management]
3. I feel like the people in my group are doing a good job cooperating right now. [cooperation]
4. Overall, I feel like things are going well in the token task situation right now. [overall]

Needs

1. I feel a sense of security, about the way the token task situation is going right now. [security]
2. I think we are sharing the tokens fairly among the members of our group now. [equity]
3. The way the token task situation is going right now makes me feel competent. [capability]
4. I am satisfied with how much control I have over the token task situation and my group right now. [control]
5. I feel like I belong in my group [belong]

DeCaro, D.A., Janssen, M.A., & Lee, A. (2021). Motivational foundations of communication, voluntary cooperation, and self-governance in a common-pool resource dilemma. *Current Research in Ecological and Social Psychology*, 2:100016.

Tenbrunsel, A. E., & Messick, D. M. (1999). Sanctioning systems, decision frames, and cooperation. *Administrative Science Quarterly*, *44*(4), 684–707.

Sheldon, K. M., Elliot, A. J., Kim, Y., & Kasser, T. (2001). What is satisfying about satisfying events? Testing 10 candidate psychological needs. *Journal of Personality and Social Psychology*, 80(2), 325-339.

Ryan, R. M. (1982). Control and information in the intrapersonal sphere: An extension of cognitive evaluation theory. *Journal of Personality and Social Psychology, 43,* 450-461.

Ajzen (2002/2006). Constructing a TpB questionnaire: conceptual and methodological considerations. [online] <https://www.semanticscholar.org/paper/Constructing-a-TpB-Questionnaire%3A-Conceptual-and-Ajzen/6074b33b529ea56c175095872fa40798f8141867>

Cameron, J. E. (2004). A three-factor model of social identity. *Self and Identity, 3,* 239-262.

### 2.2.2 Survey 2

*Administered at the end of the experiment. Assessed standard demographics and a social value orientation scale for use in a separate, meta-analysis study.*

# 3.0 CODING METHODOLOGY

To analyze group communication, we used an updated version (2.0) of DeCaro’s (2023) *Codebook for Analyzing Content and Function of Communication in Social-Ecological Experiments*, which is public archived and available at the Open Science Framework ([link](https://doi.org/10.17605/OSF.IO/FU84J)). The codebook describes the basic data preparation and coding procedures. In this section, we describe the coding methodology we used to analyze group communication to identify conservation agreements, enforcement systems, conceptual understanding of enforcement, and the degree to which group used democratic decision-making to make constitutional decisions about their fundamental social contracts (e.g., conservation agreements).

## 3.1 Conservation Agreements

To identify each group’s conservation agreement(s), we coded their in-game communication. DeCaro’s Codebook 2.0 directs analysts to identify all major constitutional decisions made by the group, including decisions about conservation agreements and enforcement systems. Such decisions typically begin with a proposed conservation strategy [proposal] and end with group selection (2 or more members voice their choice). Groups often discuss multiple proposals within/across a series of such “decision events.” The lead project PI and senior research assistant separately identified and labeled all decision events, then collaborated over several meetings to reach consensus, before beginning to co-identify conservation strategies [cf. 5]. Each decision event was labeled by the observed conservation strategies. To identify conservation strategies, we used standard identifiers developed in prior experiments using the same task environment [3,6]. Groups typically create strategies with one or more of these elements:

- ***Private Property*.** Groups divide the playing field into even sections, with each individual have completely independent or quasi-independent control of “their” section. Typically, groups divide the field into four, equally-sized corners.
- ***Delayed Harvest.*** Group members wait a particular length of time before harvesting any tokens. Common delays are 30, 60, or 120 seconds, but any is possible.
- ***Cultivate Clusters.*** Group members attempt to encourage growth of clustered tokens by not harvesting from clustered tokens until near the end of the round. They do harvest lone tokens (single tokens) that are not associated with a cluster.
- ***Thin Clusters.*** Group members attempt to cultivate clusters while selectively harvesting a small number (1 to 2) of tokens from the center of the cluster during the round. At the end of the round they harvest everything, including the clusters. This strategy is an approximation of the superior, checkerboard harvest pattern.
- ***Checkerboard Pattern.*** Group members attempt to encourage maximal regrowth during the round by harvesting tokens in a checkerboard pattern (harvesting tokens such that there is one open space between every-other token): x o x o x

o x o x o

- ***Slower Harvest.*** Group members slow down the rate of harvest (i.e., they do not harvest as fast as possible).
- ***Cyclic Harvest.*** Group members harvest in a cyclic fashion (e.g., harvest 30 seconds, pause 30 seconds).
- ***End-of-Round Harvest***. Group members wait until a specific amount of time is left at the end of the round to harvest (e.g., wait until the *last* 30, 45, or 60 seconds to harvest).
- ***Quota/Equality.*** Group members divide the available tokens up equally among the players, ensuring that each person collects the same number of tokens each round (e.g., everyone collects 75 tokens).
- ***Free-for-All at End-of-Round.*** Group members harvest anywhere they want and as quickly as they want *at the end of the round*.

We identified each instance of these strategy elements in each group’s decision events.

To simply analysis, we categorized each group’s strategy into five categories. These categories are nested, representing strategies that increase in complexity (add more elements) and, therefore, potential optimality [cf. 7,8]. For example, Category 1 represents slower harvest or private property; Category 3 additionally includes strategies to cultivate clusters (stock piles) of the resource. Coders selected the lowest code (0-4) that provided the best match to the strategies observed in the communication.

1. **None.** Group has no strategy (free-for-all).
2. **Slow or Private Property.** The group either uses (a) at least one method to slow the rate of harvest (i.e., slow down, delay harvest, end-of-round harvest, cyclic harvesting) OR (b) private property (not both “a” and “b”). *Note: end-of-round harvest may be a free-for-all or a faster final harvest without free-for-all.*
3. **Slow and Private Property.** The group uses a combination of (a) at least one method to slow the rate of harvest and private property.
4. **+Sustainable Management of Clusters.** In addition (or instead of private property), the group cultivates/grows clusters until end of round.
5. **+Checkerboard.** In addition (or instead of private property), the group harvests the tokens in a checkerboard pattern, quasi-checkerboard pattern, thins clusters by selectively harvesting tokens from the middle of clusters.

We coded/recorded each group’s final strategy at the end of each communication round, so that change in conservation strategy could be tracked across communication rounds (see Section 4.2 *Conservation Strategies*).

## 3.2 Enforcement Systems (ES)

To identify each group’s enforcement strategies, we identified each group’s constitutional decision events pertaining to the use of monetary penalties, using the same methods mentioned earlier (Section 3.1) to identify decisions about conservation agreements. To our knowledge prior research has not systematically coded enforcement systems created by self-governing groups in prior experiments. We therefore developed the following categories based on theory, knowledge of empirical case studies of community-based self-governance in the field (e.g., Ostrom, 1990) and lab (Ostrom et al., 1992), and direct observation in the current experiment [cf. DeCaro (2011) Codebook 2.0]:

1. **N/A** (none created; no formal enforcement system/agreement).
2. **Do Not Use.** The group *actively*chooses not to use monetary penalties (by vote or voiced consensus). In essence, this arrangement constitutes an agreement to voluntarily cooperate or a "treaty" against using economic sanctions.
3. **Independent Sanctions.** Group members decide that individuals may independently use penalties to punish individuals who violate the group's conservation or enforcement agreement(s).
4. **Coordinated Punishment.** Group members decide to coordinate their penalties to punish/deter someone who violates the group’s agreement(s). For example, two or more players agree to both penalize anyone who breaks an agreement.

Given the novelty of this coding system, we (Lead PI and a second, junior research assistant) collaborated to develop the initial coding categories based on a subset of groups. After revising the coding categories, the two coders separately coded all groups (blinded); inter-coder reliability was high, indicating sufficient reliability of the coding system (*Kappa*=.78).

## 3.3 Conceptual Understanding of Enforcement (CUE)

We used a similar approach in developing, and assessing the reliability of, the coding system to identify group’s conceptual understanding of enforcement (CUE). CUE was based on group’s discussion of the pros/cons, rationales, perceived effects, and believed effects or implications of using monetary penalties (sanctions), observed in their communication. Individuals often discussed the merits/implications of penalties within decision events about potential enforcement systems; however, individuals also discussed these ideas outside specific events. Therefore, when coding CUE, we examined (included) any/all statements about penalties, enforcement, etc.

We used the following coding categories, again inspired by theory [7,8]. These categories represent increasing sophistication in the group’s understanding of enforcement, increasing from no apparent understanding (i.e., fails to discuss or can provide no rationale), to understands the costs/drawbacks of using penalties, to also understands potential benefits and ways to mitigate the costs.

- 1. **None.** Did not discuss enforcement (use of monetary penalties) or discussed it but could not (or did not) figure out a rationale/purpose for its use. Example: “P1: Why would we want to use monetary penalties? P2: I have no idea.”

- 1. **Too Costly/Harmful.** Conceptualize enforcement (monetary penalties) as too costly, harming oneself, and/or harming everyone if used. Example: “P1: Monetary penalties are stupid. We should not use them, because it only hurts us. P2: yeah for real.”
  2. **Useful Deterrent.** Conceptualize enforcement (monetary penalties) as useful for correcting or preventing (deterring) rule/agreement violations. Example: “P1: I don't really like them [penalties] unless we are trying to keep each other from causing the tokens not to regenerate. P2: same”
  3. **Efficient Deterrent and/or Credible Threat.** Recognize or act upon the assumption that monetary penalties are more beneficial/effective (i.e., more potent) and/or efficient (i.e., less costly to any single individual) when they are coordinated. Example: “P3: But who would administer the fine? Everyone?” P1: Yeah, itd hurt more And you cant retaliate against 3 people as easily.”

Intercoder reliability was high (*Kappa=*.86) indicating excellent reliability.

## 3.4 Democratic Decision-Making (DDMI)

To assess the extent to which groups made constitutional decisions democratically, we coded and computed a democratic decision-making index (DDMI) proposed by DeCaro [6] based on prior indices by Vollan (2008) [9] used during voting]. After identifying each decision event (e.g., group decision about conservation agreements), we determined how many people endorsed a particular option, by explicitly voicing their support. The number of people endorsing an option can be interpreted as a proxy for self-determination and fairness, with more endorsement indicating greater self-determination and fairness in the decision process. For example, four people choosing a particular option can be assumed more fair and self-determined than if two people chose the option. This number has previously been shown to correlate strongly with perceived self-determination and fairness [6]. In a four-person group, this methodology results in the following categories (and ordinal values): unresolved/contested (0), unilateral (1), 2-person majority (2), 3-person majority (3), and consensus (4). To get an overall score (DDMI) for each group, we coded each decision event and then took the average.

# 4.0 ADDITIONAL ANALYSES

## 4.1 Perceptions and Motivations

Here, we report the descriptive statistics and results of analysis comparing treatment effects for perceptions of the dilemma, need satisfaction, rule acceptance and internalization, and cohesion (self-other merging and trust. As noted in the main paper, there were no consistent or substantive differences in these factors by treatment. Descriptive statistics for these factors appear in Table S1. Statistical results are reported in each subsection below.

**Table S1. Descriptive Statistics (Perceptions and Motivations)**

|  | **T1 (CNN)** | **T2 (FNN)** | **T3 (NCN)** | **T4 (NFN)** | **T5 (PCN)** | **T6 (PFN)** |
| --- | --- | --- | --- | --- | --- | --- |
| **Variable** | *Md*(*IQR*) | *Md*(*IQR*) | *Md*(*IQR*) | *Md*(*IQR*) | *Md*(*IQR*) | *Md*(*IQR*) |
| Percept D | 6.13(2.19) | 6.38(0.44) | 6.38(1.06) | 6.53(0.53) | 5.81(0.97) | 6.40(1.48) |
| Need Satis | 7.37(2.63) | 7.81(0.94) | 7.88(0.88) | 7.84(0.34) | 7.31(1.59) | 7.50(1.39) |
| Rule Accept | 6.50(1.50) | 6.75(0.42) | 6.75(1.50) | 6.83(0.33) | 6.33(0.96) | 6.63(1.21) |
| Rule Intern | 6.00(0.88) | 6.38(0.88) | 6.50(0.75) | 6.44(0.50) | 6.25(0.81) | 6.19(0.94) |
| Soc Press | 4.46(1.58) | 5.00(1.79) | 5.21(1.75) | 4.23(1.26) | 4.58(1.33) | 4.96(1.64) |
| Ant Earn | 6.38(0.63) | 6.25(0.50) | 6.23(1.00) | 6.50(0.75) | 6.38(1.19) | 6.44(1.06) |
| Fear Sanct | 4.50(2.38) | 4.88(1.63) | 5.63(1.88) | 5.25(1.47) | 4.75(1.63) | 5.38(1.31) |
| Cohesion | 5.58(1.58) | 6.08(0.83) | 6.08(1.25) | 6.08(0.65) | 5.83(1.50) | 6.13(1.23) |

*Percept D* (group perceptions of the dilemma). *Need Satis* (group need satisfaction in the dilemma). *Rule Accept* (group rule acceptance). *Rule Intern* (group rule internalization). *Soc Press* (cooperative motivation: group social pressure). *Ant Earn* (cooperative motivation: group anticipated earnings of conservation strategy/agreement). *Fear Sanct* (cooperative motivation: group fear of economic sanctions). *Cohesion* (group self-other merging and trust). *Md* (median). *IQR* (interquartile range).

### 4.1.1 Perceptions of the Dilemma

To examine perceptions of the dilemma as a function of treatment, we combined (averaged) the dilemma perception items (perceived chaos *reverse-scored*, resource management, cooperation, and overall) into a single factor. We then analyzed treatment differences using the Independent Median Test. Overall, no significant effects emerged among the treatments, *Independent-Samples Median Test (5)* = 3.59, *p*=.610 (Fig S1). Pairwise comparisons revealed no reliable/consistent differences, though PCN exhibited slightly worse perceptions of the dilemma than NFN (Table S2).

**Fig S1. Perceptions of the Dilemma** Table S2. Perceptions of the Dilemma


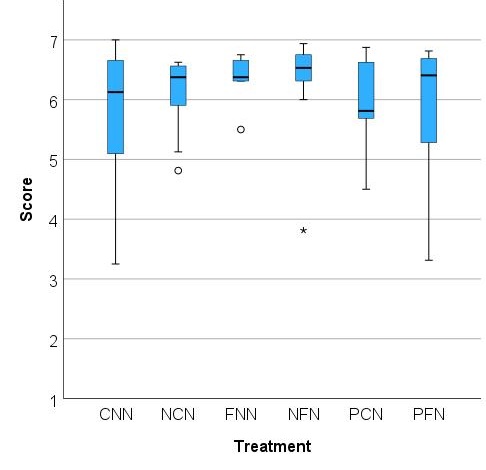


| Comparison | Test Statistic | Sig. |
| --- | --- | --- |
| PCN-CNN | 0.168 | .682 |
| PCN-FNN | 1.510 | .219 |
| PCN-NCN | 1.510 | .219 |
| PCN-PFN | 0.987 | .320 |
| PCN-NFN | 3.033 | .082 |
| CNN-FNN | 0.188 | .665 |
| CNN-NCN | 0.188 | .665 |
| CNN-PFN | 0.048 | .827 |
| CNN-NFN | 1.066 | .302 |
| FNN-NCN | 0.000 | 1.000 |
| FNN-PFN | 0.048 | .827 |
| FNN-NFN | 0.465 | .495 |
| NCN-PFN | 0.048 | .827 |
| NCN-NFN | 0.465 | .495 |
| PFN-NFN | 0.181 | .671 |

### 4.1.2 Need Satisfaction

To examine need satisfaction (in the dilemma) as a function of treatment, we combined (averaged) the dilemma perception need satisfaction items (security, equity, capability, control, and belonging) into a single factor. We then analyzed treatment differences using the Independent Median Test. Overall, no significant effects emerged among the treatments, *Independent-Samples Median Test (5)* = 4.87, *p*=.432 (Fig S2). Pairwise comparisons revealed no reliable/consistent differences (Table S3).

**Fig S2. Need Satisfaction** Table S3. Need Satisfaction


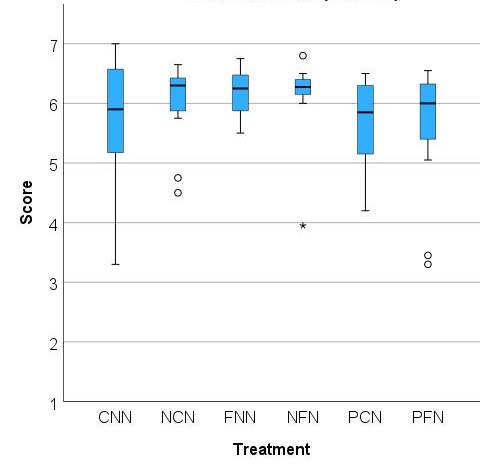


| Comparison | Test Statistic | Sig. |
| --- | --- | --- |
| PCN-CNN | 0.168 | .682 |
| PCN-PFN | 0.051 | .821 |
| PCN-FNN | 0.621 | .431 |
| PCN-NFN | 1.033 | .310 |
| PCN-NCN | 0.168 | .682 |
| CNN-PFN | 0.034 | .855 |
| CNN-FNN | 0.182 | .670 |
| CNN-NFN | 0.465 | .495 |
| CNN-NCN | 0.182 | .670 |
| PFN-FNN | 2.112 | .146 |
| PFN-NFN | 0.735 | .391 |
| PFN-NCN | 2.112 | .146 |
| FNN-NFN | 0.051 | .821 |
| FNN-NCN | 0.182 | .670 |
| NFN-NCN | 0.244 | .622 |

### 4.1.3 Cooperative Motivations

To understand potential treatment effects on cooperative motivation, we assessed a range of motivations, including rule acceptance, rule internalization, social pressure, anticipated earnings, and fear of economic sanctions, commonly measured in this environment [4,6]. As noted in the main paper, we observed no consistent or reliable patterns, which could account for observed differences in cooperation. This is most likely due to the global benefit of open communication and democratic decision-making, which most groups achieved (as reported earlier): open communication and democratic-decision making are typically associated with improved rule acceptance, internalization, and anticipated earnings. Since most groups achieved high democratic decision-making across all conditions, we would not anticipate treatment differences in their downstream motivations. Other motivational factors (e.g., social pressure, fear of economic sanctions) are more idiosyncratic to the particular group (not treatment), for example, natural variation in groups’ use of social sanctions such as warnings.

#### 4.1.3.1 Rule Acceptance

To examine rule acceptance as a function of treatment, we combined (averaged) the rule acceptance items into a single factor. We then analyzed treatment differences using an Independent Median Test. Overall, no significant effects emerged among the treatments*, Independent-Samples Median Test (5)* = 6.45, *p*=.265 (Fig S3). Pairwise comparisons revealed a potential difference between NFN and both PCN and PFN, with PCN and PFN both having somewhat lower acceptance than NFN (Table S4). This potential difference seems to be driven by the comparatively low variability among groups in the NFN condition, which is likely to be idiosyncratic rather than a reliable occurrence (for perspective see also, Rule Internalization in the next subsection).

**Fig S3. Rule Acceptance** Table S4. Rule Acceptance


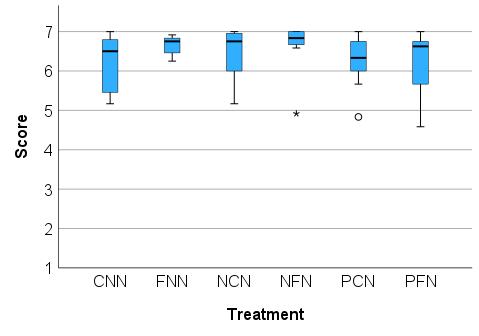


| Comparison | Test Statistic | Sig. |
| --- | --- | --- |
| PCN-CNN | 0.168 | .682 |
| PCN-PFN | 0.037 | .848 |
| PCN-FNN | 1.386 | .239 |
| PCN-NCN | 2.593 | .107 |
| PCN-NFN | 3.240 | .072 |
| CNN-PFN | 0.048 | .827 |
| CNN-FNN | 0.182 | .670 |
| CNN-NCN | 0.786 | .375 |
| CNN-NFN | 2.231 | .135 |
| PFN-FNN | 1.155 | .283 |
| PFN-NCN | 2.246 | .134 |
| PFN-NFN | 4.473 | .034 |
| FNN-NCN | 0.188 | .665 |
| FNN-NFN | 1.066 | .302 |
| NCN-NFN | 0.108 | .742 |

#### 4.1.3.2 Rule Internalization

To examine rule internalization as a function of treatment, we combined (averaged) the rule internalization items into a single factor. We then analyzed treatment differences using an Independent Median Test. Overall, no significant effects emerged among the treatments*, Independent-Samples Median Test (5)* = 3.11, *p*=.684 (Fig S4). Pairwise comparisons revealed no reliable/consistent differences (Table S5).

**Fig S4. Rule Internalization** Table S5. Rule Internalization


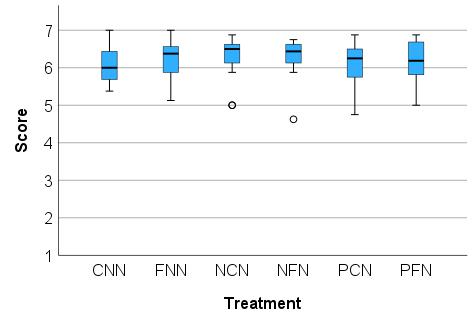


| Comparison | Test Statistic | Sig. |
| --- | --- | --- |
| CNN-PFN | 0.048 | .827 |
| CNN-PCN | 0.120 | .729 |
| CNN-FNN | 0.786 | .375 |
| CNN-NFN | 1.326 | .250 |
| CNN-NCN | 1.692 | .193 |
| PFN-PCN | 0.071 | .790 |
| PFN-FNN | 2.112 | .146 |
| PFN-NFN | 0.735 | .391 |
| PFN-NCN | 1.051 | .305 |
| PCN-FNN | 1.510 | .219 |
| PCN-NFN | 1.033 | .310 |
| PCN-NCN | 1.386 | .239 |
| FNN-NFN | 0.051 | .821 |
| FNN-NCN | 0.182 | .670 |
| NFN-NCN | 0.001 | .973 |

#### 4.1.3.3 Social Pressure

To examine social pressure as a function of treatment, we combined (averaged) the social sanctions, social disapproval, and guilt items into a single factor. We then analyzed treatment differences using an Independent Median Test. Overall, no significant effects emerged among the treatments*, Independent-Samples Median Test (5)* = 4.98, *p*=.418 (Fig S5). Pairwise comparisons revealed no reliable/consistent differences (Table S6).

**Fig S5. Social Pressure** Table S6. Social Pressure


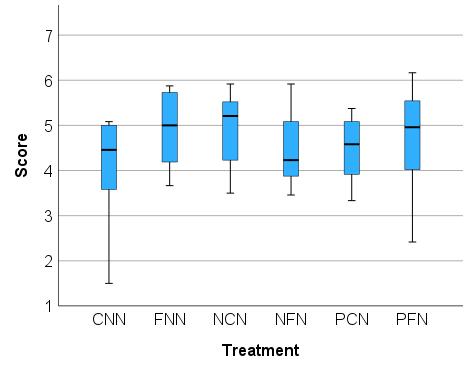


| Comparison | Test Statistic | Sig. |
| --- | --- | --- |
| NFN-CNN | 0.337 | .561 |
| NFN-PCN | 1.801 | .180 |
| NFN-FNN | 1.924 | .165 |
| NFN-PFN | 2.476 | .116 |
| NFN-NCN | 1.924 | .165 |
| CNN-PCN | 0.168 | .682 |
| CNN-FNN | 0.182 | .670 |
| CNN-PFN | 1.110 | .292 |
| CNN-NCN | 0.182 | .670 |
| PCN-FNN | 1.510 | .219 |
| PCN-PFN | 0.987 | .320 |
| PCN-NCN | 0.168 | .682 |
| FNN-PFN | 0.048 | .827 |
| FNN-NCN | 0.182 | .670 |
| PFN-NCN | 0.381 | .537 |
| Comparison | Test Statistic | Sig. |
| NFN-CNN | 0.337 | .561 |
| NFN-PCN | 1.801 | .180 |
| NFN-FNN | 1.924 | .165 |
| NFN-PFN | 2.476 | .116 |
| NFN-NCN | 4.812 | .028 |
| CNN-PCN | 0.168 | .682 |
| CNN-FNN | 0.733 | .392 |
| CNN-PFN | 1.110 | .292 |
| CNN-NCN | 1.636 | .201 |
| PCN-FNN | 1.510 | .219 |
| PCN-PFN | 3.222 | .073 |
| PCN-NCN | 1.510 | .219 |
| FNN-PFN | 1.110 | .292 |
| FNN-NCN | 0.182 | .670 |
| PFN-NCN | 0.381 | .537 |

#### 4.1.3.4 Anticipated Earnings

To examine anticipated earnings as a function of treatment, we combined (averaged) the anticipated earnings items into a single factor. We then analyzed treatment differences using an Independent Median Test. Overall, no significant effects emerged among the treatments*, Independent-Samples Median Test (5)* = 7.13, *p*=.211 (Fig S6). Pairwise comparisons revealed potential for participants in FNN to report lower motivation to cooperate due anticipated earnings than NCN and NFN (Table S7). However, these differences appear to be driven by a single outlying group in FNN

**Fig S6. Anticipated Earnings** Table S7. Anticipated Earnings


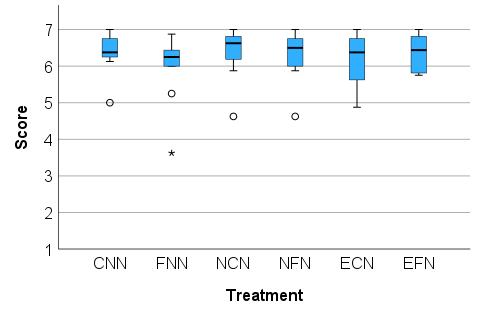


| Comparison | Test Statistic | Sig. |
| --- | --- | --- |
| FNN-CNN | 0.786 | .375 |
| FNN-PCN | 0.734 | .392 |
| FNN-PFN | 1.110 | .292 |
| FNN-NFN | 3.381 | .066 |
| FNN-NCN | 4.545 | .033 |
| CNN-PCN | 0.001 | .973 |
| CNN-PFN | 0.048 | .827 |
| CNN-NFN | 0.017 | .897 |
| CNN-NCN | 0.000 | 1.000 |
| PCN-PFN | 0.037 | .848 |
| PCN-NFN | 0.054 | .816 |
| PCN-NCN | 1.510 | .219 |
| PFN-NFN | 0.004 | .951 |
| PFN-NCN | 0.023 | .879 |
| NFN-NCN | 0.001 | .973 |

#### 4.1.3.5 Fear of Economic Sanctions

To examine fear of economic sanctions as a function of treatment, we combined (averaged) the economic sanctions motivation items into a single factor. We then analyzed treatment differences using an Independent Median Test. Overall, no significant effects emerged among the treatments*,* though the results indicate some potential differences, *Independent-Samples Median Test (5)* = 9.76, *p*=.082 (Fig S7). Pairwise comparisons revealed potential for CNN participants to report lower fear of enforcement than NCN, and potentially somewhat less fear than NFN and PFN (Table S8). This is likely due to the relatively higher frequency of the “Do not use” and “Nothing” enforcement systems in CNN and PFN. PFN also exhibited higher fear of enforcement as a motivator for compliance compared to PCN. This finding is likely due to the relative lack of enforcement in PCN and ineffectual nature of penalties when used in PCN (in other words, penalties were not a strong deterrent in PCN even when used).

**Fig S7. Fear of Monetary Penalties** Table S8. Fear of Economic Sanctions


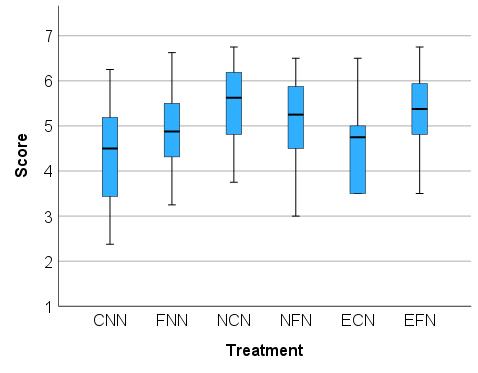


| Comparison | Test Statistic | Sig. |
| --- | --- | --- |
| CNN-PCN | 0.734 | .392 |
| CNN-FNN | 0.733 | .392 |
| CNN-NFN | 3.381 | .066 |
| CNN-PFN | 3.569 | .059 |
| CNN-NCN | 4.545 | .033 |
| PCN-FNN | 0.168 | .682 |
| PCN-NFN | 0.942 | .332 |
| PCN-PFN | 6.740 | .009 |
| PCN-NCN | 4.033 | .045 |
| FNN-NFN | 0.051 | .821 |
| FNN-PFN | 1.110 | .292 |
| FNN-NCN | 1.636 | .201 |
| NFN-PFN | 0.181 | .671 |
| NFN-NCN | 1.924 | .165 |
| PFN-NCN | 0.068 | .795 |

### 4.1.4 Group Cohesion: Self-Other Merging and Trust

To examine group identity/cohesion and trust as a function of treatment, we combined (averaged) the self-other merging items and trust item into a single factor. We then analyzed treatment differences using the Independent Median Test. Overall, no significant effects emerged among the treatments*, Independent-Samples Median Test (5)* = 2.48, *p*=.780 (Fig S8). Pairwise comparisons revealed no reliable/consistent differences, though PCN exhibited slightly worse self-other merging and trust than NFN (Table S9).

**Fig S8. Group Cohesion**  Table S9. Group Cohesion


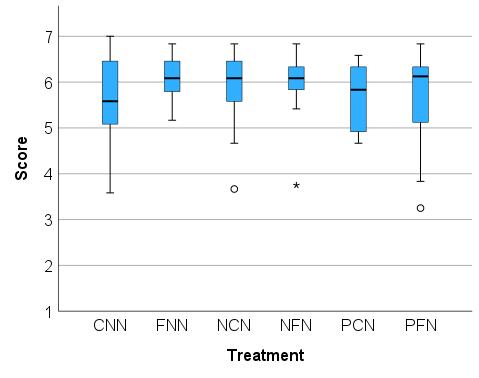


| Comparison | Test Statistic | Sig. |
| --- | --- | --- |
| CNN-PCN | 0.168 | .682 |
| CNN-FNN | 0.182 | .670 |
| CNN-NCN | 0.182 | .670 |
| CNN-NFN | 0.051 | .821 |
| CNN-PFN | 0.048 | .827 |
| PCN-FNN | 1.510 | .219 |
| PCN-NCN | 0.621 | .431 |
| PCN-NFN | 3.033 | .082 |
| PCN-PFN | 0.987 | .320 |
| FNN-NCN | 0.000 | 1.000 |
| FNN-NFN | 0.017 | .897 |
| FNN-PFN | 0.048 | .827 |
| NCN-NFN | 0.017 | .897 |
| NCN-PFN | 0.048 | .827 |
| NFN-EFN | .133 | .716 |

## 4.2 Conservation Strategies

Here, we report the frequency that particular conservation strategies (agreements) were used by groups in each of the conditions. We also report the significance tests. Fig S9 presents each treatment’s average (median) conservation strategy score (0, 1, 2, 3, 4) across each round of communication (Round 1, 2, 3). Table S10 shows the frequency of final conservation strategies in each treatment. Fig S10 shows boxplots depicting the distribution of scores; for simplicity we show each group’s final conservation strategy (typically Rd 3 strategy). As mentioned in the main paper, though there were no overall differences in conservation strategy among treatments, *Independent-Samples Median Test (5)* = 2.48, *p*=.780, FNN did exhibit a more sophisticated strategy (Table S11). However, as mentioned, this did not translate into better cooperation/performance.

**Fig S9. Evolution of Conservation Strategy**


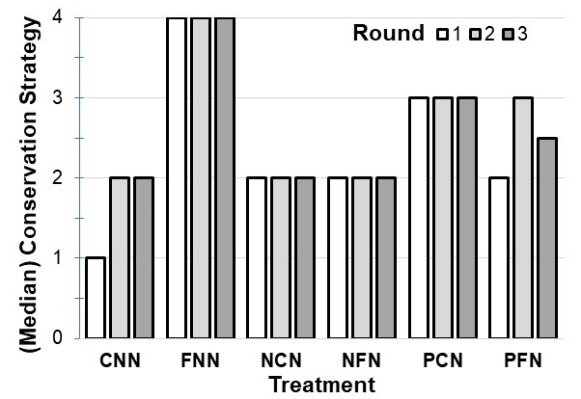


The figure shows the average (median) conservation strategy score for each treatment during Rounds 1, 2, and 3 of communication.

**Table S10. Final Conservation Strategy**

|  | None | Slow or Private | Slow & Private | Clusters | Checker |
| --- | --- | --- | --- | --- | --- |
| CNN | 0 (0%) | 4 (29%) | 5 (36%) | 0 (0%) | 5 (36%) |
| FNN | 0 (0%) | 0 (0%) | 2 (18%) | 1 (9%) | 8 (73%) |
| NCN | 0 (0%) | 2 (18%) | 4 (36%) | 2 (18%) | 3 (27%) |
| NFN | 2 (18%) | 0 (0%) | 4 (36%) | 0 (0%) | 5 (45%) |
| PCN | 0 (0%) | 1 (8%) | 2 (15%) | 4 (31%) | 6 (46%) |
| PFN | 1 (8%) | 1 (8%) | 4 (33%) | 4 (8%) | 5 (42%) |

The table shows how many groups (number, percentage) used particular conservation strategies in each treatment. Ind (*independent sanctions*). Coord (*coordinated sanctions*).

**Fig S10. Final Conservation Strategy** Table S11. Final Conservation Strategy


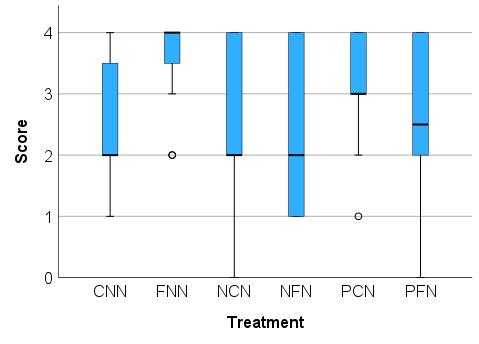


| Comparison | Test Statistic | Sig. |
| --- | --- | --- |
| CNN-NCN | 0.000 | 1.000 |
| CNN-NFN | 0.244 | .622 |
| CNN-PFN | 0.048 | .827 |
| CNN-PCN | 0.906 | .341 |
| CNN-FNN | 4.545 | .033 |
| NCN-FNN | 0.000 | 1.000 |
| NFN-FNN | 0.000 | 1.000 |
| NCN-NFN | 0.244 | .622 |
| NCN-PFN | 0.048 | .827 |
| NCN-PCN | 0.001 | .973 |
| NFN-PFN | 0.540 | .462 |
| NFN-PCN | 0.304 | .581 |
| PFN-PCN | 0.051 | .821 |
| PFN-FNN | 0.000 | 1.000 |
| PCN-FNN | 0.000 | 1.000 |

**References**

[1] M.A. Janssen, A. Lee, T.M. Waring, Experimental platforms for behavioral experiments on social-ecological systems, Ecol. Soc. 19 (2014) art20. https://doi.org/10.5751/ES-06895-190420.

[2] M.A. Janssen, Introducing Ecological Dynamics into Common-Pool Resource Experiments, Ecol. Soc. 15 (2010) art7. https://doi.org/10.5751/ES-03296-150207.

[3] M.A. Janssen, R. Holahan, A. Lee, E. Ostrom, Lab Experiments for the Study of Social-Ecological Systems, Science. 328 (2010) 613–617. https://doi.org/10.1126/science.1183532.

[4] D.A. DeCaro, M.A. Janssen, A. Lee, Synergistic effects of voting and enforcement on internalized motivation to cooperate in a resource dilemma, Judgm. Decis. Mak. 10 (2015) 511–537.

[5] E. Ratajczyk, U. Brady, J.A. Baggio, A.J. Barnett, I. Perez-Ibarra, N. Rollins, C. Rubiños, H.C. Shin, D.J. Yu, R. Aggarwal, J.M. Anderies, M.A. Janssen, Challenges and opportunities in coding the commons: problems, procedures, and potential solutions in large-N comparative case studies, Int. J. Commons. 10 (2016) 440. https://doi.org/10.18352/ijc.652.

[6] D.A. DeCaro, M.A. Janssen, A. Lee, Motivational foundations of communication, voluntary cooperation, and self-governance in a common-pool resource dilemma, Curr. Res. Ecol. Soc. Psychol. 2 (2021) 100016. https://doi.org/10.1016/j.cresp.2021.100016.

[7] E. Ostrom, Governing the Commons: Evolution of Institutions for Collective Action, Cambridge University Press, 1990.

[8] E. Ostrom, J. Walker, R. Gardner, Covenants with and without a Sword: Self-Governance Is Possible, Am. Polit. Sci. Rev. 86 (1992) 404–417. https://doi.org/10.2307/1964229.

[9] B. Vollan, Socio-ecological explanations for crowding-out effects from economic field experiments in southern Africa, Ecol. Econ. 67 (2008) 560–573. https://doi.org/10.1016/j.ecolecon.2008.01.015.
