# Supplementary material for: Learning from regulatory failure: How Ostrom’s restorative justice design principle helps naïve groups create wiser enforcement systems to overcome the tragedy of the commons
Source: PLoS One. 2024 Aug 23;19(8):e0307832. doi: 10.1371/journal.pone.0307832 (PMC11343373; doi:10.1371/journal.pone.0307832)
Supplement: S1 Protocol — (DOC) [file pone.0307832.s002.doc]

**TREATMENT 6 (PFN)**

Phase 1 (P) – Phase 2 (F) – Phase 3 (N)

**STEPS TO SET UP PROGRAM FOR AN EXPERIMENT**

**!! You must do this step before running the experiment**

1. **LOAD THE CORRECT TREATMENT**
2. First, **check the *Treatment Order Log*** to make sure you are about to run the correct treatment condition for that day/session.
3. **Load Treatment 6 (T6):** **Do these steps at Experimenter’s Station in Lab*
4. Open a Command Terminal (right click, *open terminal*)
5. Enter the following into the command lines (see picture):

cd ~/work/foraging-configuration [press enter]

**./uol.sh t6**  [press enter] **This runs a script to get T6 (from GitHub)*

<Enter Username> ###### [press enter] **This is GitHub account*

<Enter Password> #### [press enter] **Account password*

1. **If you’re successful** a “successful build” message will be displayed. Close the terminal, and move on to the next steps.

*If you make a mistake at any point above, start over from the beginning.

1. **START THE SERVERS**
2. Do these steps at the Experimenter’s Station in the Lab.

*These steps tell you how to launch the Facilitator Window, which is the interface that lets you monitor and operate the components of the experiment.*

1. First start the Maven/Jetty Server.[[1]](#footnote-2) Our experimenter station has two network cards. So, we need to tell the computer which network card (server) to use for the Maven server.
2. Open a new terminal (right click, *open terminal*)
3. Enter the following into the command lines (see picture):
   1. cd ~/work/foraging
   2. mvn jetty:run
4. If you did this step correctly, you should see this as the last line in the terminal window: “[INFO] Started Jetty Server” (see picture).


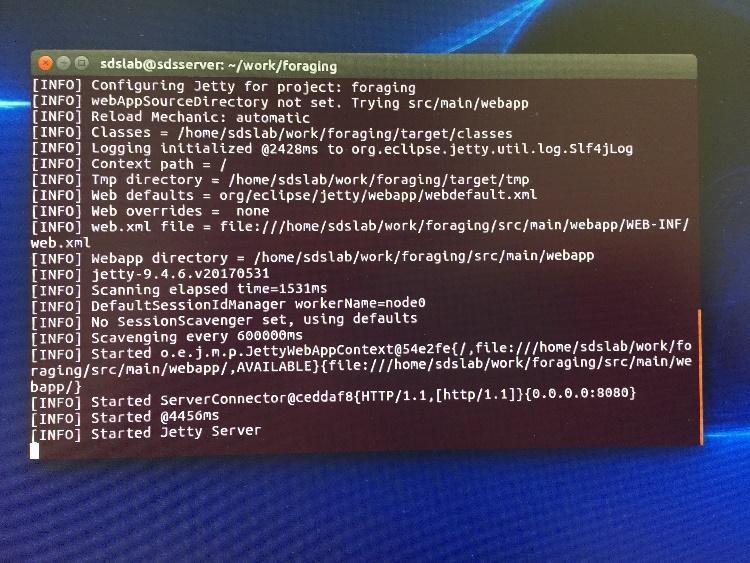


1. DO NOT close the window. It must remain open the entire experiment.
2. **Next start the Ant Server.**
3. Open a second terminal (right click, *open terminal*)
4. Enter the following into the command lines (see picture):
   1. cd ~/work/foraging
   2. ant server
5. If you did this step correctly, you should see this as the last line in the terminal window: “[java] INFO: Waiting for all quizzes to be submitted.” (see picture below):


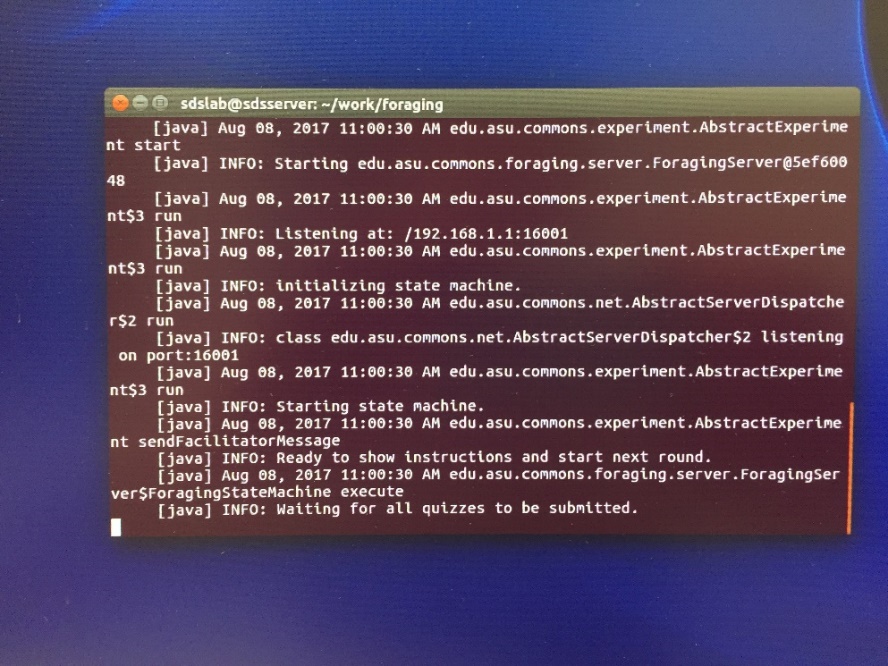


1. **DO NOT close the window.** It must remain open the entire experiment.
2. **Launch the Facilitator and Clients**

***Attention:** The ***facilitator.jnlp* file should be on the desktop of the Experimenter Station**, and the ***client.jnlp* file should be on the desktop of each Participant Station**. If not, then go to guide called *How to Download the Facilitator and Client jnlp files*.

1.
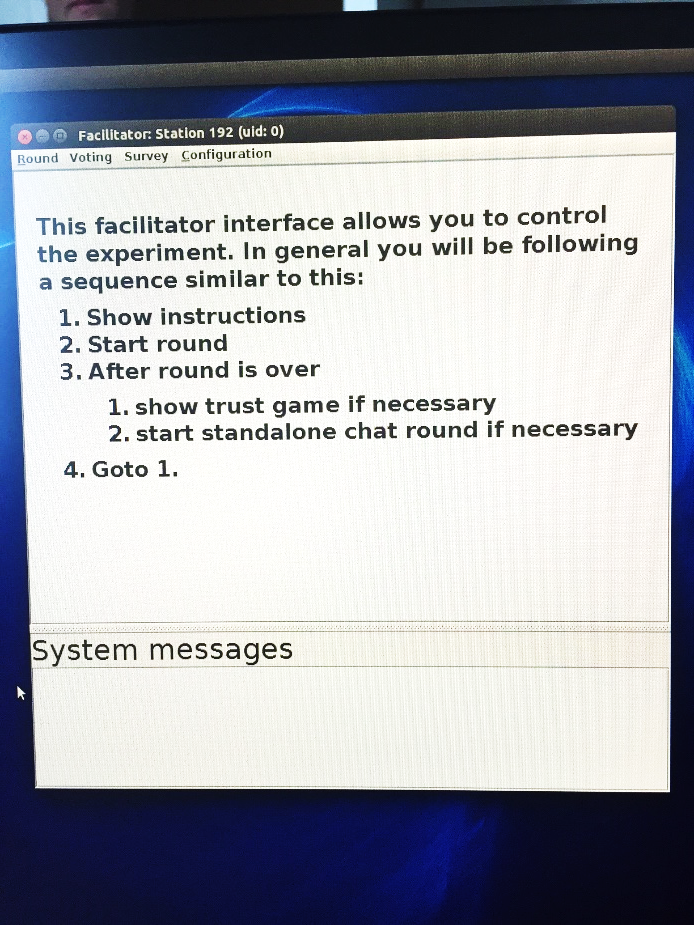
**Launch the Facilitator Window**
2. Do this at the **Experimenter Station**.
3. Double click the *facilitator.jnlp* file on the Desktop.
4. A warning message pops up. Accept the risk, click ok.
5. The Facilitator Window should launch (see picture):

***IMPORTANT:** If you did this correctly, then the *uid number* will be zero “0.”

***IMPORTANT:** If you did this correctly, then the *uid number* will be zero “0.”

1. **Launch the Client Windows**
2. Do this at **each Participant Station**.
3. Only launch 8 computers at first.
4. Launch them in this order: Station 1, 2, 5, 6, 7, 8, 11, 12.
5. Double click the *client.jnlp* file on the Desktop.
6. A warning message pops up. Accept the risk, click ok.
7. The “Welcome” screen should appear (see picture):


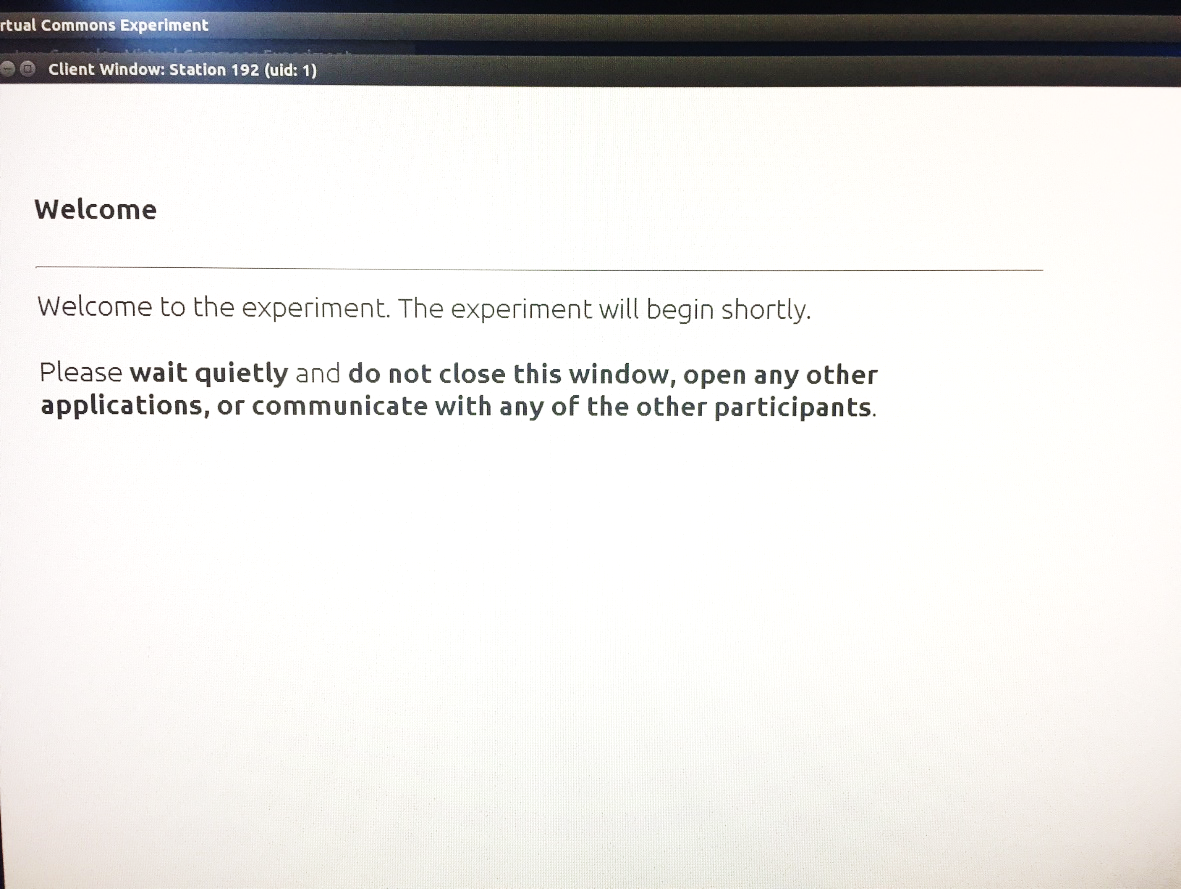


1. Record the *uid#* of each station on the *Session Log*. If you did this correctly, then the *uids* should be: Station 1 (uid 1), Station 2 (uid 2), Station 5 (uid 3), Station 6 (uid 4), Station 7 (uid 5) Station 8 (uid 6) Station 11 (uid 7), Station 12 (uid 8)
2. **Only launch the remaining 4 client windows (Stations 3, 4, 9, 10) when 12 people have actually arrived to the study location (during Consent).**

**THE PROGRAM IS READY!**

**GENERAL INSTRUCTIONS, QUIZ, AND PRACTICE**

**Introductory Instructions & Quiz**

**SAY:** Okay. From this point forward, I will give you important instructions and other information on your computer. Be sure to read all the information completely and carefully.


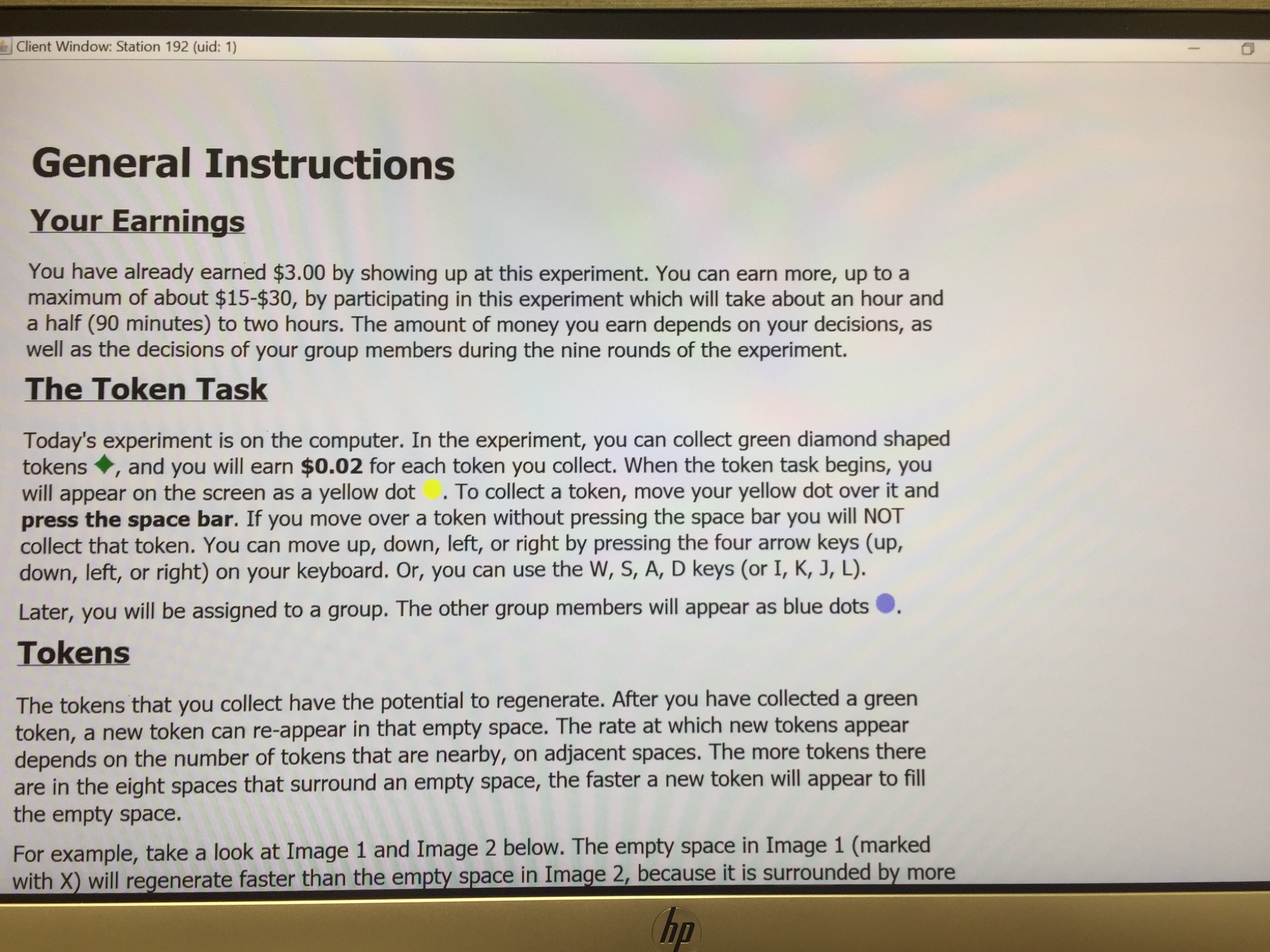


1. **(Facilitator)**

**Round 🡪 Show Instructions (General Instructions)**

**SAY:** You should see the introductory instructions on your screen now. It will explain the basic information about today’s decision task. You can go ahead and read that now, and complete everything on the page, to get started. Please be sure to scroll down to see everything.

- Also, please do not open other programs, or close this program. Our software is a little sensitive, so if you accidentally close the wrong thing, you might freeze the experiment.
- And, if a pop-up appears on the screen (e.g., if a program tries to update itself), just try to ignore it. If it becomes a problem let me know, so I can take care of it.
- Finally, from this point forward we ask that you do not communicate with each other or look at other people’s screens.

**Quiz and Quiz Results**

*Participants complete a Quiz at the bottom of the General Instructions Screen. After that, they are taken automatically to a Quiz Results screen, where they review their answers. After reviewing, they press Continue to go the Practice Instructions Screen.*

🡪*Wait for everyone to complete the quiz, review the results, and read the Practice Instructions.*

***After 1-2 minutes** **SAY:**

Has everyone had time to read the General Instructions and complete the Quiz at the bottom of the screen? After you complete the Quiz, review your results and press Continue to go to instructions for a Practice Round. (If you have any questions, just raise your hand)

**Practice Round**

***After 1 minute, SAY:** Has everyone had time to read the Practice Round Instructions?


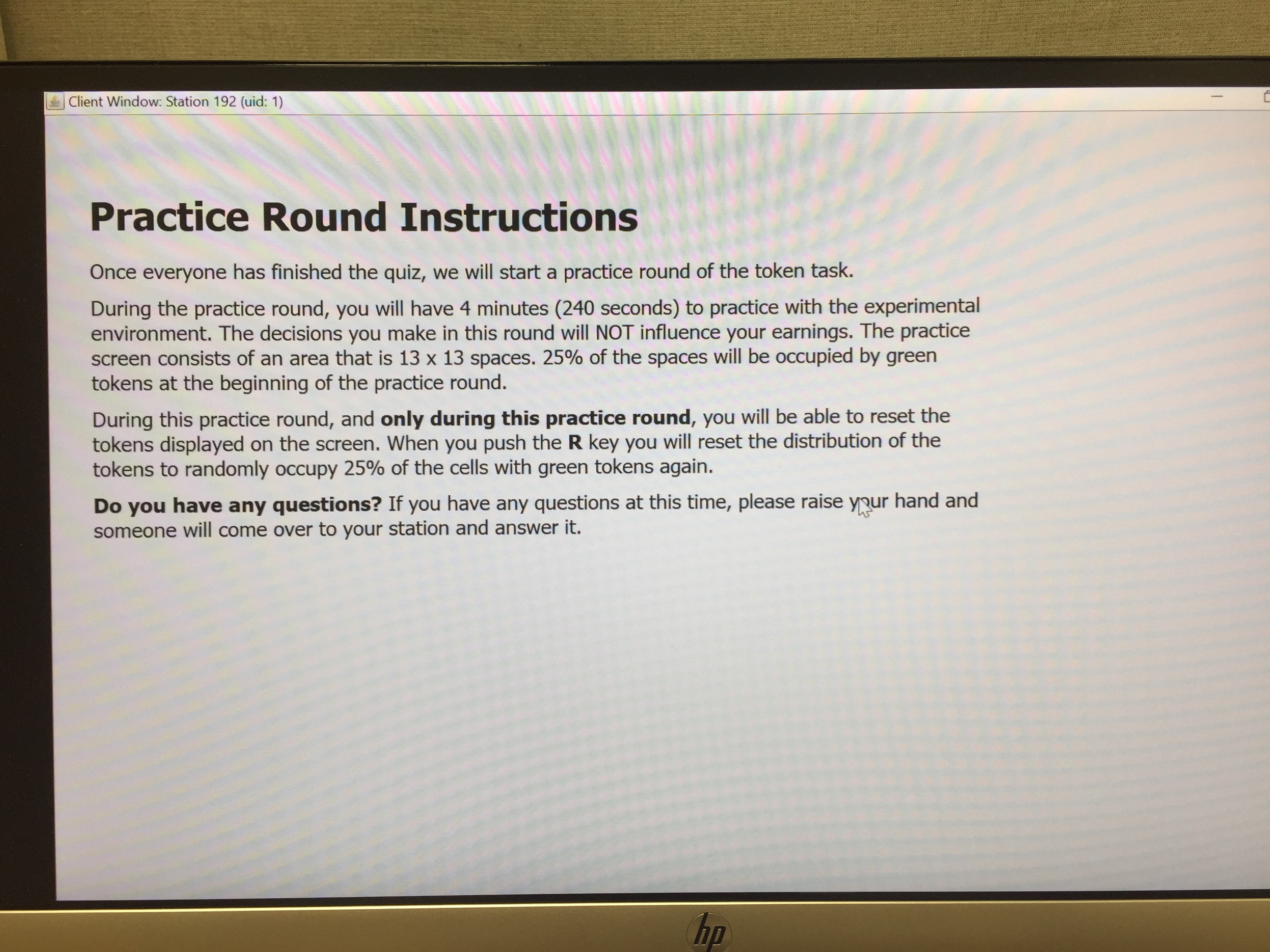


**SAY:** Okay. We’ll do a practice round next. Any questions before we get started? (If you have a question at any point during the experiment, just raise your hand)

- You will do all your movement with the arrow keys and pick up tokens with the spacebar. You need to press the arrow key again each time you want to move.

1. **(Facilitator)** **Round 🡪 Start (Practice Round)**


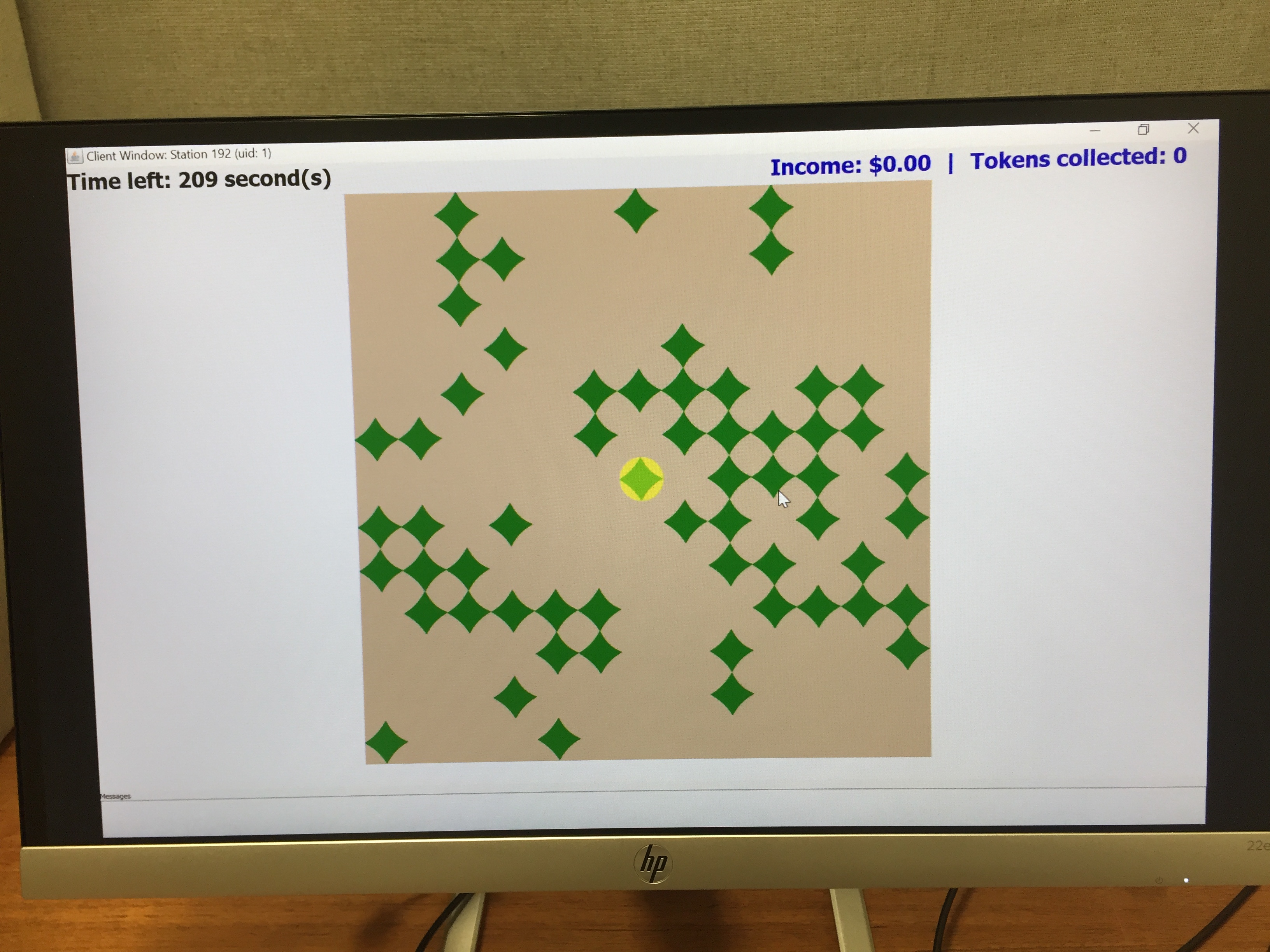


*Practice Round Results will be displayed automatically (after 4 minutes).*


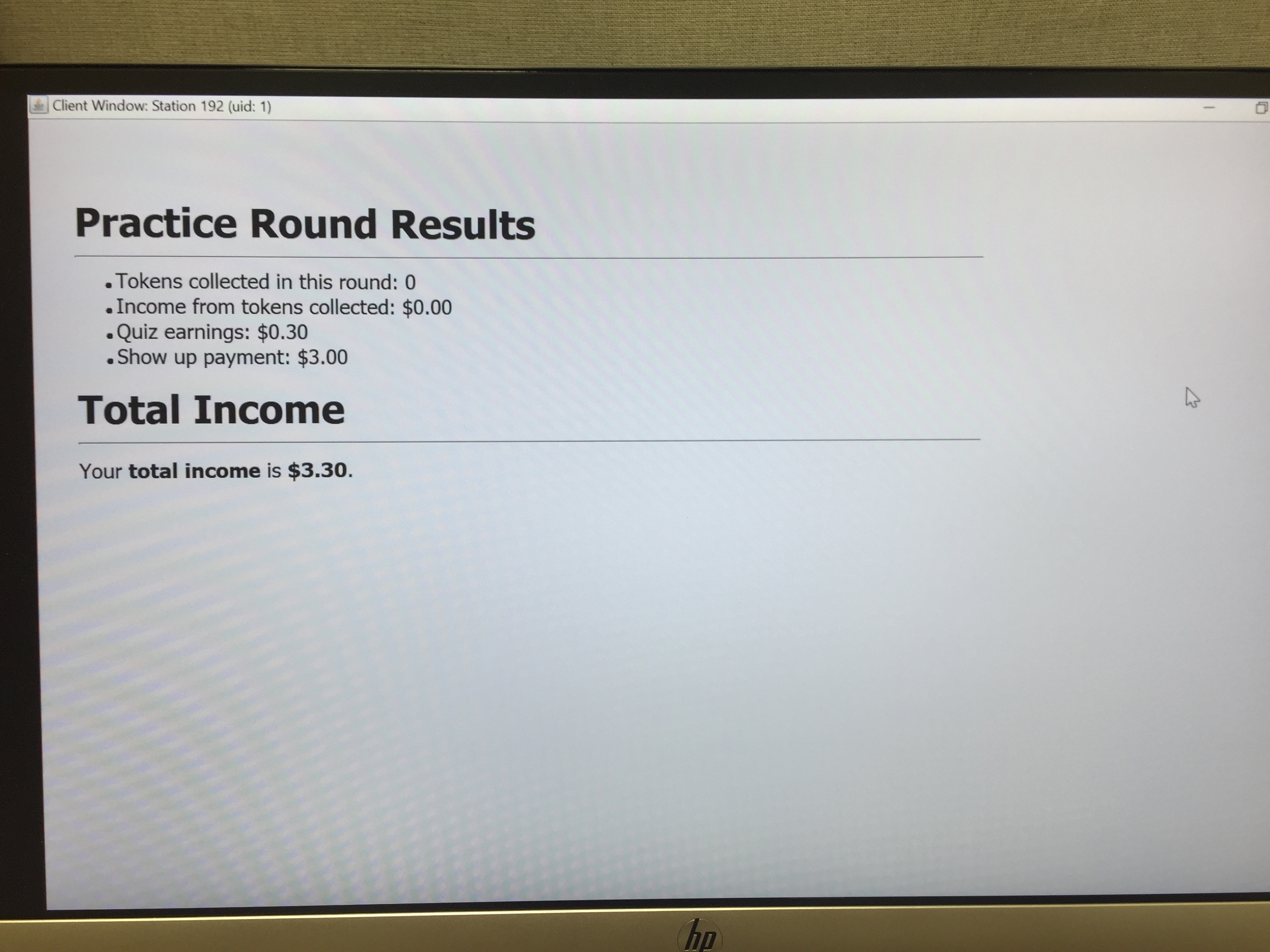
 *Let Ps review this for about 20 seconds.

**PHASE 1: Initial Monetary Penalties (P)**

**Round 1**

1. **(Facilitator) Round 🡪 Show Instructions (Round 1 Instructions)**

- **SAY:** Okay the Instructions for Round 1 are on the screen now.


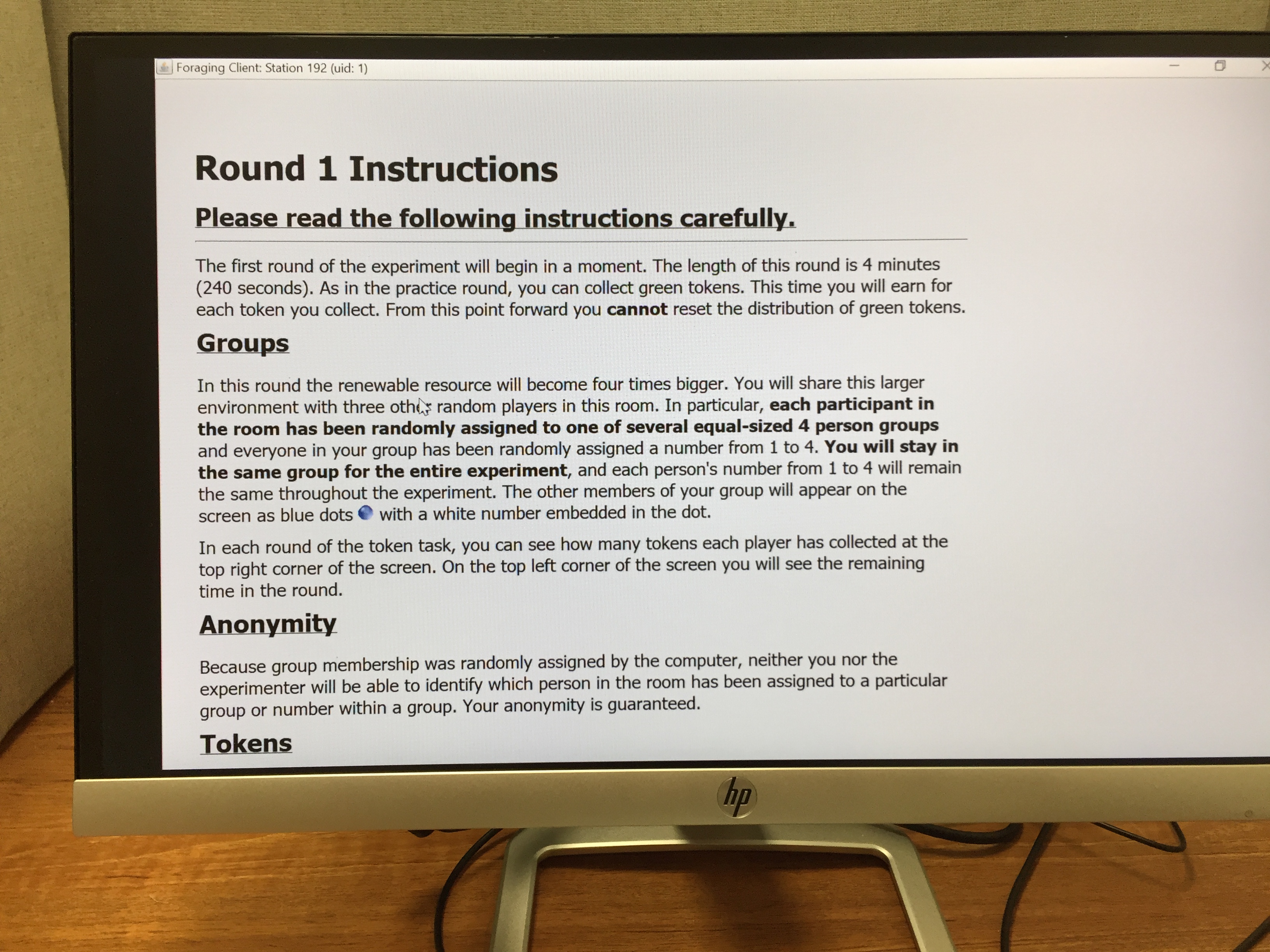


- *Wait for everyone to read (about 1 minute)…*

**SAY:** Okay. We’re ready to begin the first round of the experiment. From this point forward, you will get 2 cents for each token you collect.

I’ll explain a few important points to make sure everyone understands.

Each of you has been randomly assigned to a group with 4 people in it (and each of you has a Player ID number from 1 to 4 within that group). The members of your group will stay the same throughout the entire experiment. And your numbers will too. However, you will not be able to identify which person in the room has been assigned to your group, because it was randomly assigned.

I also can’t determine what group you are in, or any of your other behavior either for this same reason. The computer will keep track of your earnings and report it anonymously, so that you get paid what you earn at the end of the experiment, without being specifically identified to me.

Any questions?

**Tell participants about communication**

**SAY:** At this point, I have important new instructions to inform you about.

1. **“Monetary Penalties Instructions”**

**(Facilitator) Round 🡪 Show NEXT INSTRUCTIONS**

***Wait for about 1 minute 🡪***

**SAY:** Starting with this round, you have the option to place monetary penalties on other people in your group. This means you can use some of the money you collect to penalize another person.

Each time you want to penalize a player, find their number key at the top of the keyboard and press that number (1, 2, 3, or 4). For example if you want to penalize Player 1 press the number 1, push number 2 for Player 2, push number 3 for Player 3, or number 4 for Player 4. When you penalize someone, 1 of your tokens (2 cents) will be used to remove 2 tokens (4 cents) from that person. You can do this as much as you want, as long as you have at least 1 token that round, and the other person has some tokens, too. If you do not have at least 1 token, then you cannot make a monetary penalty. When someone uses a monetary penalty or is penalized, a message will be displayed on the right-hand side of the screen. Their player avatars will also flash for a moment.

Do you have any Questions?

1. **(Facilitator) Round 🡪 Start (“Round 1”)**

Round 1 Results will be displayed automatically (after 4 minutes).

- *Let Ps review for about 20 seconds (10 secs from now on).*

**Round 2**

1. **(Facilitator) Round 🡪 Show Instructions**

**SAY (after 10 seconds):** Ok. Round 2 instructions are on the screen now. I will start the round now.

1. **(Facilitator) Round 🡪 Start (“Round 2”)**

***Round 2 Results*** will be displayed automatically (after 4 minutes).

- *Let Ps review for about 10 seconds.*

**Round 3**

1. **(Facilitator) Round 🡪 Show Instructions**

**SAY:** Ok. Round 3 instructions are on the screen now. (Pause) I’ll start the round now.

1. **(Facilitator) Round 🡪 Start (Round 3)**

Round 3 Results will be displayed automatically (after 4 minutes).

**PHASE 2: Facilitated Communication and Enforcement (F)**

**Tell participants about communication**

**SAY:** At this point, I have important new instructions to inform you about.

1. **Round 4 “Communication Instructions”**

**(Facilitator) Round 🡪 Show Instructions**

**SAY:** You should be able to see the instructions on your screen now. Again, be sure to read them fully and carefully. If you have any questions, just raise your hand.


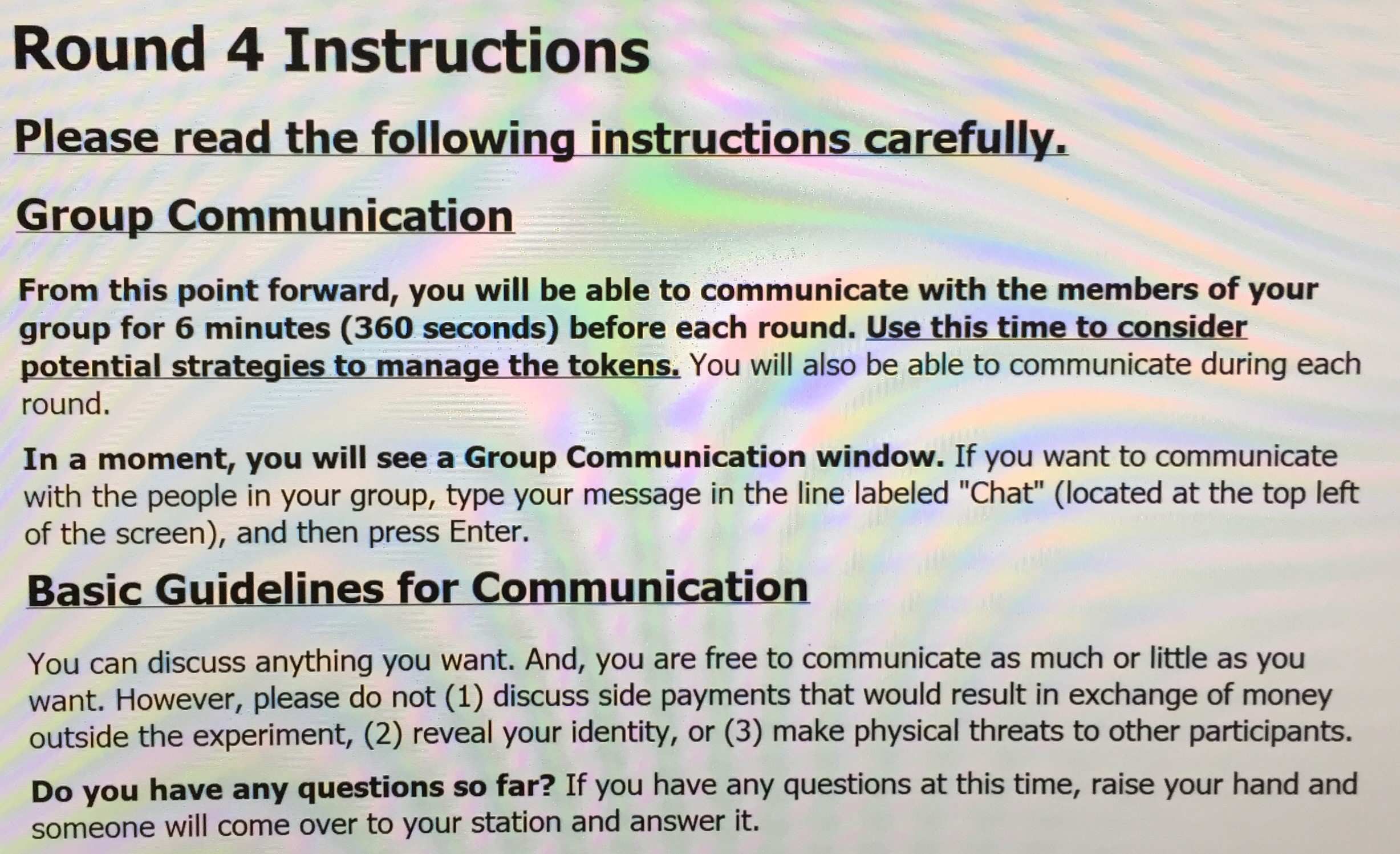
***Wait for about 1 minute 🡪***

**SAY:** Beginning with this round, you will have the option to communicate with the other members of your group by using the chat window on your computer, which you will see in a moment. When you want to say something, just type your message in the Chat field, and press enter. Your message will be displayed to your group. It will be identified only by your Player number (1 through 4).

You will have 6 minutes to communicate before each round of the token task. After that, you will also be able to communicate during each round.

You can discuss anything you want. However do not discuss side payments that would result in exchange of money outside the experiment. And, do not reveal your identity or make physical threats to other participants.

Questions?

- Ok. I’ll show the next instructions now.

1. **“Monetary Penalties Instructions”**

**(Facilitator) Round 🡪 Show NEXT INSTRUCTIONS**

***Wait for about 1 minute 🡪***

**SAY:** Like before, you will have the option to place monetary penalties on other people in your group. Each time you want to penalize someone, press the number key at the top of the keyboard that matches that person’s Player Number (1, 2, 3, or 4). 1 of your tokens (2 cents) will be used to remove 2 tokens (4 cents) from that person. You can do this as much as you want, as long as you have at least 1 token that round, and the other person has some tokens, too. If you do not have at least 1 token, then you cannot make a monetary penalty. When someone uses a monetary penalty or is penalized, a message will be displayed on the right-hand side of the screen, and the avatars will flash for a moment. …Do you have any Questions?

1. **Additional Guidelines Instructions”**

**(Facilitator) Round 🡪 Show NEXT INSTRUCTIONS**

***Wait for about 1 minute 🡪***

- **SAY:** Ok. I’m going to start the 6-minute Communication period now. As you communicate, discuss strategies or agreements your group members may be able to use to manage the collection of tokens. Also discuss whether or not your group wants to use monetary penalties. Be sure to discuss how they will be used and why. Strategies and payment reductions should be justified among the group.

Finally, as you communicate, have an open discussion where everyone can voice their opinion, and do your best to listen and consider other points of view. Try to be respectful and try not to use overly harsh language or heated arguments, if possible.

- Questions?

**Dedicated Communication 1**

Ok. I’ll start the 6-minute communication period now. To communicate with your group members type your message in the chat field at the top of the screen. Then press enter.

1. **(Facilitator) Round 🡪 Start Chat (Chat 1)**

🡪 Rd 4 Instructions will be displayed automatically.

**Round 4: “About to Begin”**

**SAY:** During this round you will also have the option to communicate with your group members. To do that, just type your message in the chat box on the right side of token task screen. After you are done using the chat box, you will need to press “enter” to exit it and return to the field of tokens.

Any questions? …I’ll start Round 4 now.

1. **(Facilitator) Round 🡪 Start (“Round 4”)**

Round 4 Results will be displayed automatically (after 4 minutes).

- *Let Ps review for about 10 seconds.*

**CONTINUE 🡪**

**Round 5 “Communication Instructions”**

**(Facilitator) Round 🡪 Show Instructions**

**SAY:** Ok. The instructions for Round 5 are on the screen. Please read them carefully.

***Wait for about 1 minute 🡪***

- **SAY:** Ok. In a moment, you will have 6 more minutes to communicate with your group members before the next round starts. Like before, feel free to discuss anything you want.

In addition, if your group used monetary penalties discuss it. Give each person that was penalized the opportunity to discuss their point of view, and try your best to address any concerns they mention. The group members that used the monetary penalty should also have the opportunity to explain their perspective. For example, someone might not understand the group’s strategy for managing the tokens, be dissatisfied with the strategy, or have a problem with the monetary penalties. Whatever the issue, try your best to communicate the reasons for your thoughts, so others can understand your perspective and why it might be important to consider. If possible, try to find a solution that satisfies the group and resolves potential conflicts.

Finally, even if your group did not use monetary penalties, if you have any concerns with the way the group is managing the tokens, or the way the group has decided to use monetary penalties, please discuss it. If possible, see if the group can find a solution that satisfies everyone, or at least improves the situation. …Are there any Questions?

**Dedicated Communication 2**

**SAY:** Ok. In a moment, you will have 6 more minutes to communicate with your group members before the next round starts. I will start the chat period now.

1. **(Facilitator) Round 🡪 Start Chat (Chat 2)**

***After it ends***, **Round 5 “About to Begin” Instructions** will be displayed automatically:

**(After 10 seconds), SAY:** Ok. Round 5 instructions are on the screen now.

1. **(Facilitator) Round 🡪 Show Instructions**

**You must do the “show instructions” command here, to activate the Round menu option.*

**SAY:** Ok. I’ll start the round now.

**Round 5**

1. **(Facilitator) Round 🡪 Start (“Round 5”)**

***Round 5 Results*** will be displayed automatically (after 4 minutes).

- *Let Ps review for about 10 seconds.*

**Round 6 “Communication Instructions”**

**(Facilitator) Round 🡪 Show Instructions**

**SAY:** Ok. The instructions for Round 6 are on the screen. Please read them carefully.

***Wait for them to read (30 seconds) 🡪***

- **SAY:** Ok. I’ll start the next 6-minute communication period in a moment. Like before, feel free to discuss anything you want. And, if you have any thoughts or concerns about the way your group is using monetary penalties, or the group’s strategy for managing the tokens, discuss them as well. As a group, consider if you can find a solution.
- Are there any questions?

**Dedicated Communication Period 3**

**SAY:** Ok. In a moment, you will have 6 more minutes to communicate with your group members before the next round starts. I will start the chat period now.

1. **(Facilitator) Round 🡪 Start Chat (Chat 3)**

***After it ends***, **Round 6 “About to Begin” Instructions** will be displayed automatically:

**(After 10 seconds), SAY:** Ok. Round 6 instructions are on the screen now.

1. **(Facilitator) Round 🡪 Show Instructions**

**You must do the “show instructions” command here, to activate the Round menu option.*

**SAY:** I’ll start the round now.

**Round 6**

1. **(Facilitator) Round 🡪 Start (Round 6)**

Round 6 Results will be displayed automatically (after 4 minutes).

**SURVEY 1**

**Say:** Before we continue to Round 7 of the token task, we would like to ask you some questions on an online survey. This survey takes about 10 to 15 minutes. In a moment, I’m going to show you an instruction screen. Click the part that says “CLICK HERE.” Then give it a couple moments to open.

1. **(Facilitator) Survey 🡪 Show Survey Instructions (Survey 1)**


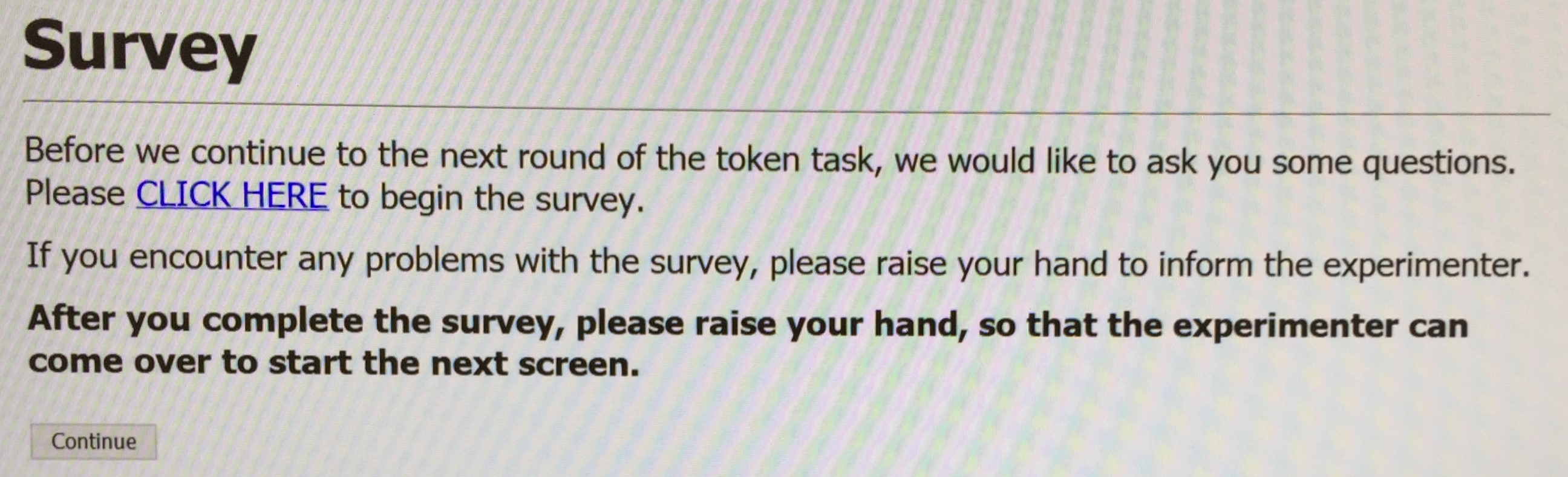

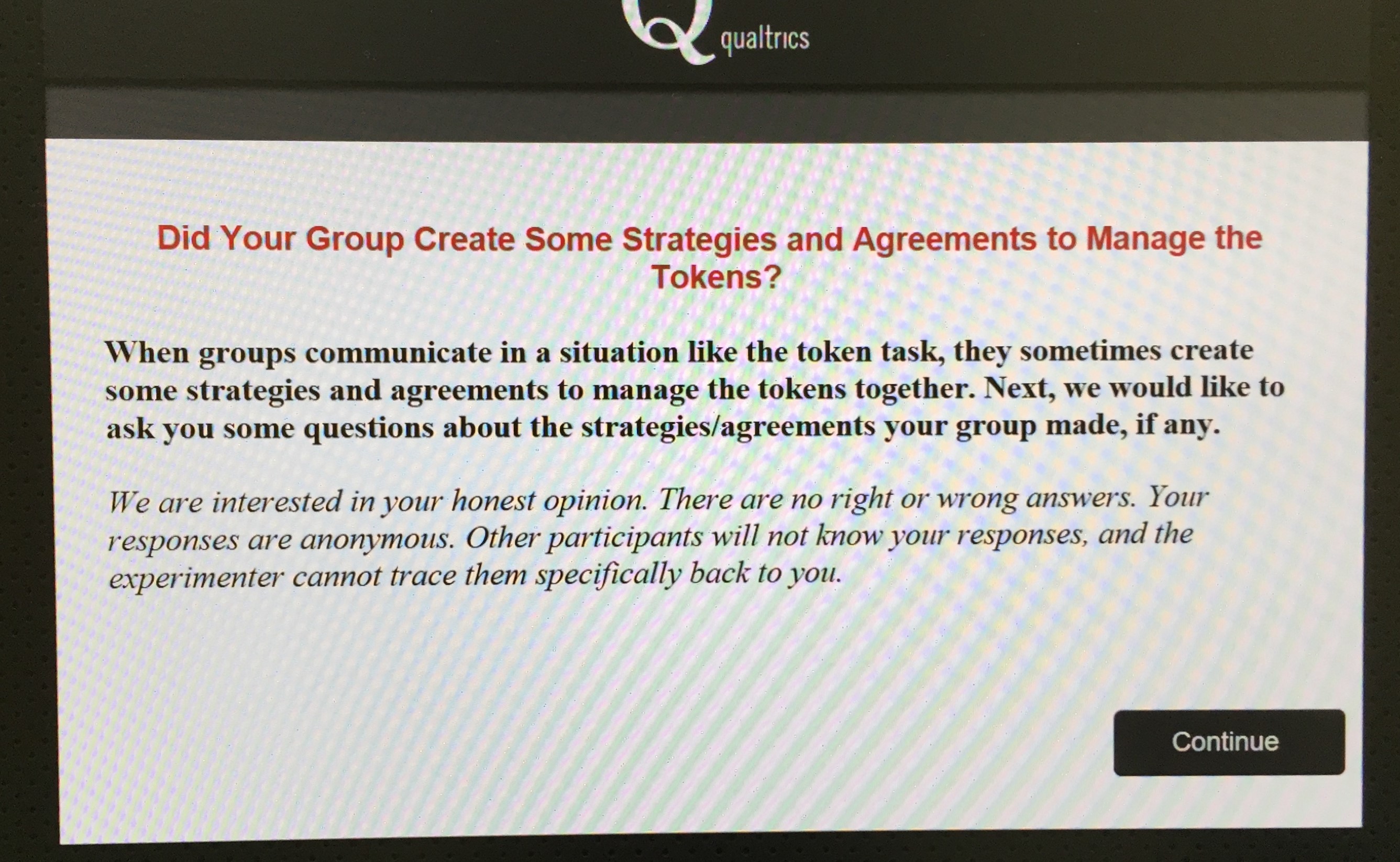


**SAY:** Okay. You should be able to see the instructions now. Click the part that says “CLICK HERE.” If you run into any problems with the survey, please raise your hand for help.

1. ***After about 20 seconds****, ask if they were able to get started okay. Then do a quick walk to check.*
2. ***After about 10 minutes****, you should start seeing people raise their hand that they are done.*(Go over and close the survey, then click “continue.”
3. ***Say:*** When you finish the survey, please raise your hand, so I can close the survey for you and get the next part of the experiment started.
4. ***If it seems like the survey is running long****, because of slow individuals say:* Has everyone had time to complete the survey? We will move forward once everyone is done. **Politely stop surveys that take more than 20 minutes (by letting them know we need to move on and then closing it).*

**AFTER SURVEY, CONTINUE 🡪**

**PHASE 3: No Communication or Enforcement (N)**

**Round 7**

**SAY:** Okay. We are about to begin Round 7 of the token task, and I have important new instructions to inform you about.

1. **(Facilitator) Round 🡪 Show Instructions (“No Communication/Penalties”)**

- **SAY :** You should be able to see the instructions now. Please read them carefully.

*…wait a moment for them to read.*

- **SAY:** Beginning this round, you will no longer have the option to communicate with the members of your group or use earning reductions to reduce the earnings of another person.

I’ll start the round now.

1. **(Facilitator) Round 🡪 Start (“Round 7”)**

Round 7 Results will be displayed automatically (after 4 minutes).

**Round 8**

1. **(Facilitator) Round 🡪 Show Instructions (Round 8 Instructions)**

Okay. Round 8 instructions are on the screen now.

I’ll start the round now.

1. **(Facilitator) Round, Start (Round 8)**

Round 8 Results will be displayed automatically (after 4 minutes).

**Round 9**

1. **(Facilitator) Round 🡪 Show Instructions (Round 9 Instructions)**

Okay. Round 9 instructions are on the screen now.

I’ll start the round now.

1. **(Facilitator) Round, Start (Round 9)**

Round 9 Results will be displayed automatically.

- **Go to next page for Exit Survey**

**SURVEY 2: Exit Survey**

**Tell participants about Survey 2 (The Exit Survey)**

**SAY:** Okay. We would like to ask you a final set of questions, in another online survey. This survey takes about 2-3 minutes to complete. When you see the instruction screen, click on the part that says “CLICK HERE” to open the survey.

1. **(Facilitator) Survey 🡪 Show Survey Instructions (“Exit Survey”)**

**Say:** Ok. You should be able to see the CLICK HERE part now. If you run into any problems with the survey, raise your hand for help.


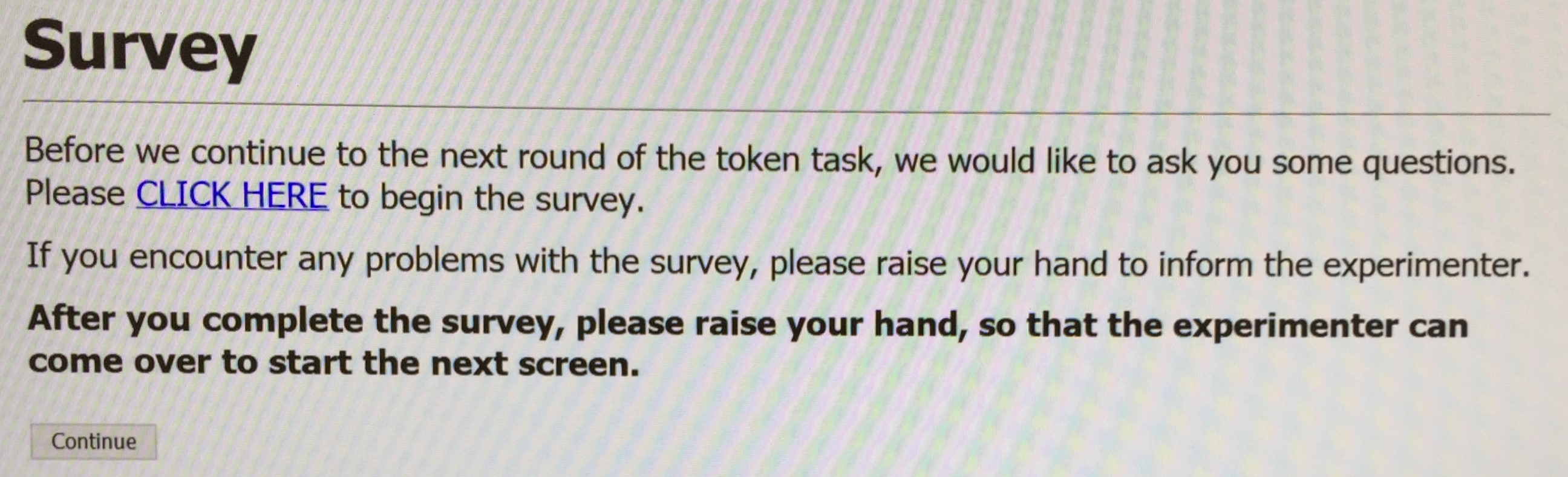

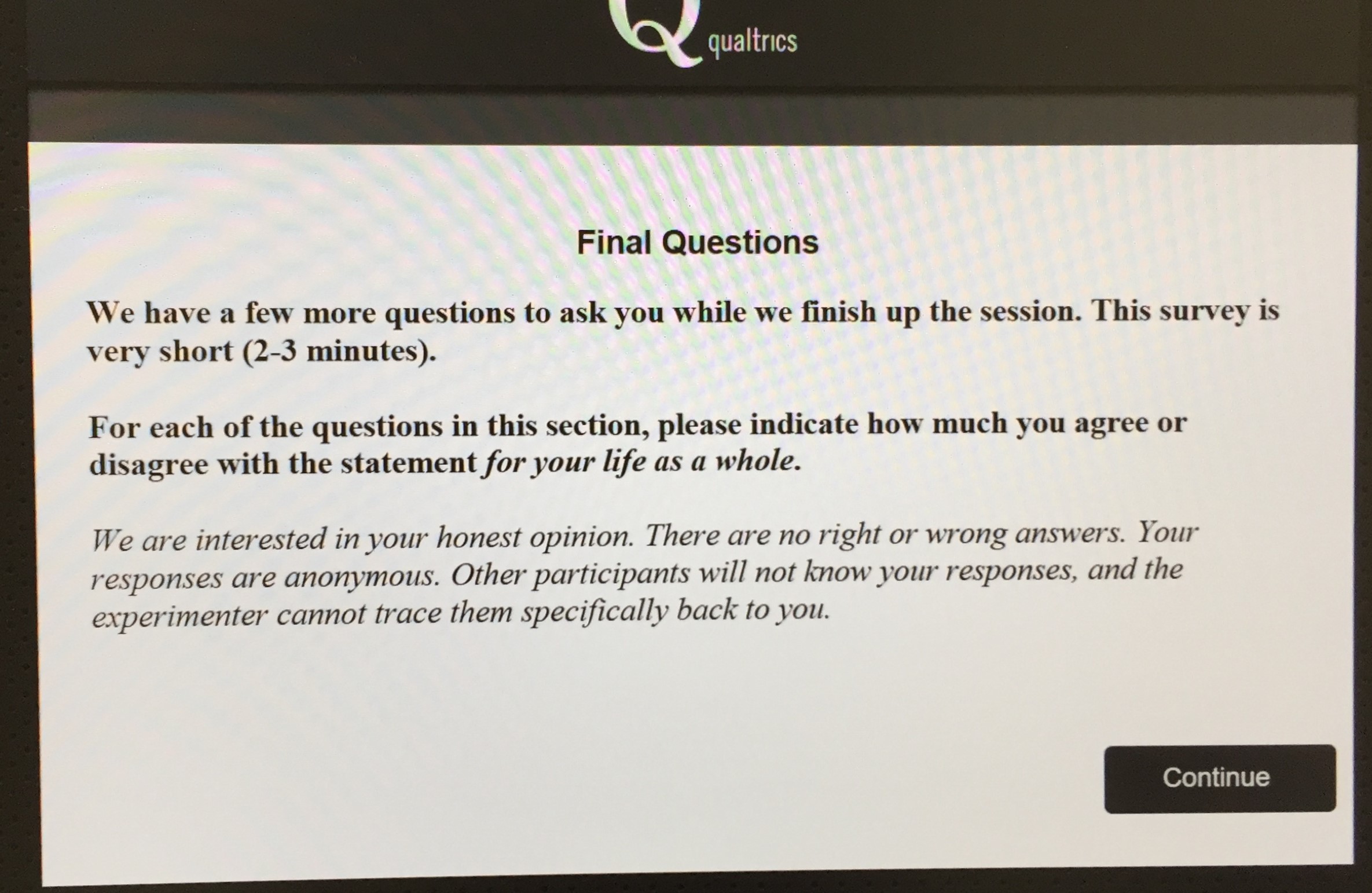


1. Assist participants in closing the survey properly and getting back to the foraging program.

**EXIT INSTRUCTIONS**

1. **(Facilitator) Round 🡪 Show Exit Instructions**

**DEBRIEFING AND PAYMENT**

1. **Pass out the Payment Slips and Pens.** Ask them to fill out the slips. *If they don’t know their Social Security Number, they could try asking a parent/guardian (or we can hold onto their completed slip until they return with that information at a later date).
2. **Read this DEBRIEFING STATEMENT:**

Thank you for your help today! Cooperation is very important to society. The goal of this research is to learn about the factors that affect people’s ability to cooperate and share valuable resources. Today, we were looking at your perceptions of the situation, and how being able to communicate with one another might influence your ability work together. We are also looking out how you might use monetary penalties, and whether that would help you to cooperate better or not. For example, sometimes groups use monetary penalties as a way to enforce the agreements and strategies they have developed. But sometimes this makes things worse. We are trying to figure out when and why it improves cooperation instead of decreasing cooperation.

It is very important that future participants do not know this information. If they know the purpose and details of the experiment it will bias their behavior and contaminate the study. So please do not tell your friends or classmates about the details of this study, in case they decide to participate in a future study. If they ask about it, please just encourage them to participate and let them know that you can discuss all the details *after* they participate.

**Do you have any questions about the study?**

(Offer to answer any questions they may have.)

1. **Explain the payment procedures**, so they know what to expect:
   1. By now, most participants should have filled out the Payment Form.
   2. You will call 2 of them up at a time. And, take them to room 207 (Research Assistant office).
   3. Payments will be made using Visa Cards (like a gift card). The money can be used in most locations.
   4. Ask the Participant to enter their information into the appropriate fields. Then finish up the rest, and ask them to check the info one last time.
   5. Then give them their card, with the value written down, in a private envelope.
   6. The Payment Form will then be shredded before they leave, so that their private information is stored only on the secured payment system.

***See the Payment Procedures guide for additional (internal) payment steps.**

**CLEAN-UP**

**Finish up Payment Procedures**

1. **Record the payments** in the *NSF_Exp2Restore_Payments* excel file, on primary payment computer.
2. Make sure the **safe is locked**, and the **key is put back** in the door lock box.

**Store Important Documents (Signed Consents, Session Logs, etc.)**

1. Update the **Session Log** with any special notes you’ve taken about the session. Put the final log in the *NSF EXP2 Restore SESSON LOG* folder, in the filing cabinet.
2. Put all **signed Consent Forms** in the *NSF EXP2 Restore CONSENT* folder, in the filing cabinet.

**Backup the Data**

1. There is a DATA thumbdrive in the Safe (with the Swift Cards)
2. Copy the experiment data and paste it into the thumbdrive.
   1. The data is stored in the *experiment-data* subfolder here:

Home🡪work🡪foraging🡪experiment-data🡪*find the folder for the date*

- 1. ***If you are the first person to run an experiment today***, there will be one dated folder for the current date. Copy it and paste it to the appropriate folder in the thumbdrive. ***If this is the second session today***, then there will be multiple files within the folder for the current date; these subfolders will have a time stamp in military time (e.g., 15:45:32, which means 3:45pm). Find the one that corresponds to your session, copy that folder and paste into the corresponding location on the thumdrive.

1. Next, take a screenshot of the Earnings Summary Table (fn + prt). Save it in the *NSFrestoreEXP2_Earnings* subfolder in the *Home* directory. Name the file using this format: “CommExp2_P1-12_8-22-17” (i.e., CommExp1_participants_date).
2. Now, copy and paste that file into the thumdrive in the appropriate folder to back it up.
3. Return the Thumbdrive to the safe.

**Reporting**

1. **Check that we have enough** Consent Forms, Debriefing slips Payment Forms, Swift Cards, Session Logs, Pencils/Pens. If not, let the Lab Coordinator know.
2. **Report the basic information** about the session (number of participants, any problems, etc.) to the Lab Coordinator and Dr. ####.
3. Prepare for Lab for the next session (otherwise, close-up the Lab).

1. **Maven/Jetty Server.** Delivers the initial client.jnlp file to each participant and the facilitator. The **JNLP
   files** are responsible for connecting to the server you started via "java -jar -server server.jar" or "ant server". After that, they are not used during the experiment, so they shouldn’t affect performance. [↑](#footnote-ref-2)
