# Supplementary material for: Learning from regulatory failure: How Ostrom’s restorative justice design principle helps naïve groups create wiser enforcement systems to overcome the tragedy of the commons
Source: PLoS One. 2024 Aug 23;19(8):e0307832. doi: 10.1371/journal.pone.0307832 (PMC11343373; doi:10.1371/journal.pone.0307832)
Supplement: S1 File — (PDF) [file pone.0307832.s003.pdf]

# CODEBOOK FOR ANALYZING CONTENT AND FUNCTION OF COMMUNICATION IN SOCIAL-ECOLOGICAL DILEMMA EXPERIMENTS

Daniel A. DeCaro*

Version 2.0 (October 25, 2022)

## Abstract

This codebook provides updated concepts and methodologies for coding and quantifying the content and function of communication in group social dilemma experiments, specifically with a social and ecological component (e.g., common pool resource dilemma). The content that is coded pertains to a refined set of 35 categories such as small talk, information exchange (e.g., ecological, economic, social, institutional), enforcement types (e.g., praise, warnings, threats), and decision-making (e.g., proposals, choosing). Functional categories pertain to key functions needed for group members to govern the dilemma: constitutional decision making (e.g., devise resource management strategies, create enforcement systems, make group decisions, form agreements), enforcement and conflict resolution, and operational activities (e.g., real-time coordination). This codebook provides guidance for metrics (e.g., indices of democratic decision making, restorative justice) to associate coded communication with observed cooperation, based on the Bloomington School of Political Economy, Elinor Ostrom's (1990, 2010) design principles for co-production and societal self-governance, and DeCaro's (2018, 2019) Humanistic Rational Choice Theory of collective action.

## Acknowledgements

This material is based upon work supported by the National Science Foundation under Grant No. 1658608. Any opinions, findings, and conclusions or recommendations expressed in this material are those of the author(s) and do not necessarily reflect the views of the National Science Foundation. The author wishes to thank Marci S. DeCaro (University of Louisville), Lianda Velic (University of Louisville), Alanea Graci (University of Louisville), and Devin Flener (University of Louisville) for their feedback and help in developing this work.

---

*Affiliation: University of Louisville, Email: daniel.decaro@louisville.edu

## TABLE OF CONTENTS

EXECUTIVE SUMMARY ..... 5
0.0 PROJECT ..... 5
0.1 REVISIONS AND UPDATES ..... 5
0.2 IMPORTANT CONSIDERATIONS ..... 6
CHAPTER 1: INTRODUCTION ..... 7
1.0 CONCEPTUAL OVERVIEW ..... 7
1.1 CODING SYSTEM OVERVIEW ..... 8
1.1.1 Coding Function ..... 8
1.1.2 Coding Topical Content ..... 9
CHAPTER 2: DATA PREPARATION ..... 11
2.0 INTRODUCTION ..... 11
2.1 UNITS OF ANALYSIS ..... 11
2.1.1 For Coding Topical Content ..... 11
2.1.2 For Functional Coding ..... 11
2.2 SEGMENTING ..... 12
2.2.1 Step 1: Combine Broken Thought Units ..... 12
2.2.2 Step 2: Reorganize into Major Discussion Topics ..... 13
2.2.3 Step 3: Identify Decision Events ..... 16
2.2.4 Step 4: Identify Restorative Justice Events ..... 20
2.2.5 Step 4: Parse Complex Thought Units into Phrases (Sub-thoughts) ..... 21
CHAPTER 3: CODING TOPICAL CONTENT ..... 23
3.0 INTRODUCTION ..... 23
3.1 CODING CATEGORIES AND DEFINITIONS ..... 24
3.1.1 Greetings [*GREET*] ..... 24
3.1.2 Goal [*GOAL*] ..... 24
3.1.3 Proposal [*PROPOSE*]: ..... 25
3.1.4 Choosing [*CHOOSE+/-*] ..... 26
3.1.5 Ecological Information [*ECO INFO*] ..... 28
3.1.6 Economic Information [*$ INFO*] ..... 28
3.1.7 Social Information [*SOC INFO*] ..... 28
3.1.8 Rule Information [*RULE INFO*] ..... 29
3.1.9 Experiment Information [*EXP INFO*] ..... 30
3.1.10 Monitor Resource [*MON RES*]: ..... 31
3.1.11 Monitor Earnings [*MON EARN*]: ..... 31

3.1.12 Monitor Behavior [*MON BEH*]: .......... 32
3.1.13 Perceived Harm [*POSE HARM*] .......... 32
3.1.14 Concern [*POSE CONCERN*] .......... 32
3.1.15 Apologize [*RJ APOLOGY*] .......... 33
3.1.16 Atone [*RJ ATONE*] .......... 33
3.1.17 Justify [*RJ JUSTIFY*] .......... 34
3.1.18 Refusal to Restore Justice [*RJ-*] .......... 34
3.1.18 Forgive [*RJ FORGIVE+/-*] .......... 34
3.1.20 Coordinate [*COORD*] .......... 35
3.1.22 Praise and Encouragement [*ENF PRAISE*] .......... 36
3.1.23 Ask [*ENF ASK*] .......... 37
3.1.24 Tell [*ENF TELL*] .......... 38
3.1.25 Shame [*ENF SHAME*] .......... 38
3.1.26 Warn [*ENF WARN*] .......... 39
3.1.27 Threaten [*ENF THREAT*] .......... 39
3.1.28 Coordinated Punishment [*ENF PUNISH*] .......... 40
3.1.29 Defection [*DEFECT*] .......... 41
3.1.30 Small Talk [*SM TALK*] .......... 41
3.1.31 Humor [*HUMOR*] .......... 41
3.1.32 Being Polite/Impolite [*POLITE+/-*]: .......... 41
3.1.33 Discourage Input [*INPUT-*] .......... 43
3.1.34 Ambiguous [*AMB*] .......... 43
3.1.35 Uncodable [*UNCODE*] .......... 43
**CHAPTER 4: FUNCTIONAL CODING .......... 44**
4.0 INTRODUCTION .......... 44
4.1 DEMOCRATIC DECISION MAKING .......... 44
4.1.1 Democratic Decision-Making Index .......... 45
4.1.2 Communication Equality .......... 46
4.1.3 Deliberative Equality .......... 46
4.2 ENFORCEMENT INDEX .......... 49
4.3 RESTORATIVE JUSTICE INDEX .......... 51
**CHAPTER 5: CODING INSTITUTIONAL ARRANGEMENTS .......... 55**
5.0 INTRODUCTION .......... 55
5.1 CONSERVATION STRATEGIES .......... 55
5.2 ENFORCEMENT SYSTEMS .......... 58

**REFERENCES ........................................................................................................................61**

## EXECUTIVE SUMMARY

### 0.0 PROJECT

This codebook is a product of a larger research project with Co-PIs Marco A. Janssen, and Allen Lee, funded by the National Science Foundation:

> Psychosocial, motivational, and cooperative effects of communication, enforcement, and participatory decision making in resource dilemmas. (2017-2023). *National Science Foundation (NSF): Decision, Risk & Management Sciences (Award #1658608)*
>
> DeCaro, D.A., Janssen, M.A., & Lee, A. (2021). Motivational foundations of communication, voluntary cooperation, and self-governance in a common-pool resource dilemma. *Current Research in Ecological and Social Psychology*, 2:100016. doi: 10.1016/j.cresp.2021.100016
>
> Janssen, M.A., DeCaro, D.A., & Lee, A. (2022). An agent-based model of the interaction between inequality, trust, and communication in common pool experiments. *The Journal of Artificial Societies and Social Simulations.*

### 0.1 REVISIONS AND UPDATES

The original codebook was developed while analyzing communication data from DeCaro, Janssen, and Lee's (2021) original experiment, which was a cooperative experiment involving communication (without economic sanctions/enforcement or any other interventions). The current Version 2.0 of the codebook introduces several changes/updates, based on observations made during that experiment as well as a second experiment, conducted by the same researchers, which included economic sanctions and additional interventions.

***Purpose:*** (a) introduce novel coding categories to better account for group dynamics in creation, use, and fairness of enforcement systems, (b) streamline existing coding categories (e.g., refine, remove redundancies) to make the coding system easier to learn and use by coders, and (c) support more targeted analysis for types of collective decision-making processes within groups (e.g., constitutional decisions regarding conservation and enforcement systems), (d) code evolution of conservation agreements and/or enforcement systems, (e) facilitate more accurate (reliable) coding, and (f) facilitate agreement among multiple coders, as well as calculation of inter-rater reliability.

***List of Updates:***

1) Modified segmentation steps to **additionally parse complex (parent) thought units into their component phrases** (sub-thought units). This refinement allows more precision and accuracy, when identifying and coding content; this precision also improves inter-rater agreement/reliability.

2) **Refined the classification of decision events**, clarifying that we focus on constitutional decisions, as well as distinguish those that pertain to conservation agreements versus enforcement systems. This refinement facilitates identification and quantification of conservation systems and enforcement systems, as well as their evolution.

3) **Added Restorative Justice Event designation** to identify group decisions that pertain to the fair resolution of harms/injustices caused by conflict, rule violations, use of enforcement, or institutional inequities (e.g., in design or systemic costs/benefits of the current institutional arrangements). This refinement captures restorative justice processes posited by Ostrom (1990) to be essential to long-term maintenance, legitimization, and evolution of cooperative governance systems (cf.DeCaro et al., 2021).

4) **Added coding categories for greetings (GREET), sharing economic information ($ Info), forgiveness of perceived harms (FORGIVE+/-), and simple real-time coordination (COORD)** to account for these previously overlooked activities. The FORGIVE category facilitates analysis of restorative justice events. The COORD category helps to differentiate coordination of conservation strategies during operational decision events (e.g., time-based coordinated harvests) from their enforcement, especially the TELL enforcement category (i.e., telling others to comply).

5) **Removed the RAT+ (Rationale) coding category**, because we observed it to be redundant with new coding for restorative justice (e.g., RJ Justify).

6) **Removed the INPUT+ coding category** because greater experience with the coding system revealed it to be (a) highly redundant with the primary content code applied to such thought units, and (b) less informative than functional codes designed to capture open deliberation by group members (e.g., the democratic decision-making index).

7) **Added methods to identify the specific institutional arrangements (conservation strategies, enforcement systems) that groups created, as well as quantify the sophistication of those arrangements.** These additions allow researchers to examine the quality of those institutional arrangements as well as keep track of institutional evolution (i.e., changes to the institutional arrangements over time).

8) **Clarity:** Many chapters and sub-sections of the codebook have been reordered, renamed, and generally revised to improve conceptual and practical clarity. The coding category definitions (and examples) have also been refined for improved clarifty.

## 0.2 IMPORTANT CONSIDERATIONS

### Work in Progress:

This is a work in progress. Subsequent versions developed by the author will be published on a rolling basis, as needed to address new experiments and observations.

### Recommended Coder Expertise:

This coding system assumes that coders are familiar with economic rational choice theory, common-pool resource and public good dilemmas (e.g., Ostrom 1990), and the specific experimental task, *Foraging Task* (see Jassen 2010, Janssen et al. 2010). This background knowledge is required to code effectively and understand the contextual meaning of participants’ communication and behavior.

# CHAPTER 1: INTRODUCTION

## 1.0 CONCEPTUAL OVERVIEW

This coding system starts with the recognition that the primary target of the investigation is problem-solving and governance in a social-ecological dilemma. Thus, communication is analyzed in light of, and in service of, problem-solving/governance in a social dilemma.

**Groups Must Solve Two Fundamental Problems (Dilemmas)**:

(1) Managing/sustaining the shared resource (resource management), to improve individual and collective earnings (**CPR Dilemma**). *First Order Dilemma (Appropriation).*

(2) Managing each other (i.e., governing) to encourage and sustain coordination and compliance (i.e., cooperation): this entails creating social order and institutions (i.e., rules, agreements, and social norms) to guide behavior (**Public Good Dilemma**: creating a governance system and social order). S*econd Order Dilemma (Provision).*

The CPR Dilemma generally cannot be reliably (robustly) solved until the Public Good Dilemma of provisioning a viable governance system (governance dilemma) is resolved (see Ostrom 1990: 38-39 *"interdependence, independent action, and collective action*," 42-50 "*three puzzles: supply, commitment, and monitoring*"; cf. Gardner et al. 1990).

**These Dilemmas Pose Several Important Challenges:**

*Governance Dilemma*

Individuals need to collectively create a system of rules, agreements, and norms to coordinate their behavior and improve resource management (**problem of supply**); they also need to find ways to create credible commitment to those governance systems to compel and bolster coordination and compliance (**problem of commitment**); they also need to monitor behavior, and enforce their rules and agreements (**problem of monitoring and enforcement**). Governance systems often need to be updated and improved to address complex patterns of behavior and new developments in the social-ecological system; this may include implicit or explicit experimentation to find effective solutions (**adaptive/transformative governance**).

*CPR Dilemma*

Individuals, and their groups, need to understand the critical ecological features, processes, and dynamics of the resource system and larger ecological system, in order to devise effective management techniques (i.e., rules, procedures, strategies). They may need to adjust these solutions over time, or experiment with new techniques to probe underlying ecological features and improve their management solutions (adaptive management).

## 1.1 CODING SYSTEM OVERVIEW

Our coding system analyzes communication in terms of multiple levels of abstraction, or complexity: coding the intended *function* or purpose of communication (e.g., democratic decision making, enforcement) and coding *how frequently* particular topics are discussed (e.g., ecological information, small talk). The reason we code in terms of both function and frequency of topical content is to capture the greatest descriptive and explanatory range of information from the group communication. In addition, topical content frequency coding is more widely used in prior research, but has yielded only limited explanatory power for understanding cooperation (e.g., Janssen 2010, Pavitt 2011). We use both methodologies to refine them and investigate their potential scientific contributions (cf. Brauner, 2018). **This method how now been tested and published with great effect in DeCaro et al. (2021), helping to account for substantial variance in observed levels of group cooperation in a resource dilemma involving communication, with no other interventions.**

### 1.1.1 Coding Function

Coding for function means that communication content and patterns are examined in terms of their apparent or hypothesized **functional** or **psychological significance** (i.e., intended purpose, meaning; cf. Deci and Ryan 1987; Tschan et al. 2018), specifically for addressing the core dilemmas and challenges posed by the governance and problem-solving tasks inherent to collective management of the social-ecological dilemma. This approach is beneficial because it identifies how individuals are conceptualizing the governance task and social-ecological dilemma, and how individuals interact with each other in a group setting (cf. Pavitt 2011).

This approach contrasts with most previous coding systems, which have primarily focused on counting the frequency of particular types of topical content that were communicated (e.g. the number of statements about ecological information), without determining the context of those statements or their intent or purpose (e.g., goals that individuals/groups were trying to accomplish when sharing ecological information). Some researchers have coded communication in social dilemmas in terms of functional categories (e.g., Pavitt 2011, Janssen 2010). Our approach builds on those prior attempts by focusing on specific governance tasks thought to be fundamental to successful governance in social dilemmas: constitutional versus operational institutional choice (collective choice and rulemaking), democratic decision-making processes (decision fairness), enforcement systems (types of monitoring and enforcement), and restorative justice (i.e., fair implementation of rules and their enforcement, as well as conflict resolution; cf. Gardner et al. 1990, Ostrom 1990):

1) **Constitutional vs. Operational Choice:** Identify major collective decision-making events, and determine whether they are constitutional or operational. ***Constitutional decisions*** pertain to decisions about the group's fundamental social contracts or institutional arrangements (e.g., conservation agreement). ***Operational decisions*** occur during the round (as players harvest tokens); these pertain to real-time coordination decisions to implement existing social contracts (e.g., conservation agreements), without substantive (i.e. constitutional) changes. Decision events (as well as enforcement and restorative justice events) are the basic unit of analysis for functional coding/analyses.
2) **Democratic Decision-Making:** Given the theoretical and empirical importance of shared (i.e., democratic) decision-making in cooperative governance systems (Ostrom, 1990, 2010; DeCaro et al., 2015, 2021), it is necessary to assess (quantify) extent of democratic decision-making across constitutional decision events. This functional

category operationally defines the coding and quantification of such democratic decision-making processes, across a group's key decisions (e.g., for comparison with other groups). This information may be correlated with behavioral outcomes (e.g., cooperation) and psychosocial measures (e.g., perceived procedural justice and self-determination).

3) **Conservation Strategies.** Groups may devise and use a range of conservation strategies to manage the shared resource. However, these strategies generally consist of one or more common elements (e.g., private property, delayed harvest, cultivating clusters). Functional coding of the specific conservation strategies allows the evolution of these strategies to be recorded and associated effects assessed.

4) **Enforcement Systems.** Identify the enforcement systems groups use to encourage and/or compel short- and long-term cooperation and compliance. These systems include positive and negative social sanctions (e.g., praise, encouragement; warnings, threats), as well as tangible financial sanctions (e.g., monetary penalties). We also identify the agreements (rules, norms) groups form to govern their use (e.g., timing, purpose, extent of such enforcement systems), and the overall balance of positive vs. negative sanctions. Doing so allows the evolution/potential effects of enforcement to be analyzed.

**5)** **Restorative Justice:** Conflict resolution and the responsiveness of cooperative systems to perceived harms/injustices (e.g., inequities in costs/benefits, unfair rule-enforcement) is believed to be essential to the long-term legitimization, effectiveness, and robustness of cooperative governance systems (Ostrom, 1990; cf. Johnstone and van Ness, 2013; Tyler, 2006a). Therefore, we identify restorative justice events and code their resolution (*Restorative Justice Index*), to assess the extent to which groups engage in restorative forms of conflict resolution and management of their enforcement systems.

### 1.1.2 Coding Topical Content

In addition to coding and evaluating large segments/passages of communication (e.g., decision events) in terms of functional categories, we code the topical content within specific lines (thought units/statements) of communication. This type of coding, which focuses on content composition and frequency, does not necessarily capture the underlying intent or function(s) contained in a dialogue. Coding topical content-line-by-line dissects larger segments of dialogue into fragments (e.g., breaks up otherwise cohesive decision events). However, topical content coding is the most common method of communication coding in the relatively few social dilemma experiments that have coded communication (Pavitt 2011, Janssen 2010, Janssen et al. 2010). In addition, coding the frequency that particular topics are discussed provides a basic description of communication patterns (Brauner 2008) and could potentially be associated with cooperation, though prior experiments have generally found little such relationship, including DeCaro et al.'s (2021) application of Version 1.0 of this coding system.

Nevertheless, **we recommend topical content coding for scientific comparison and because it serves as the basis for higher-order functional coding** (e.g., functional coding of enforcement systems requires initial line-by-line coding of topical content, to identify the types of enforcement used, and their overall proportion of use).

We identify 35 topical categories (see next page).

## Summary List of Topical Content Codes

| | | |
|---|---|---|
| 1. | **Greet** | Greetings (e.g., hi, hello, yo). |
| 2. | **Goal** | Discuss goals (e.g., sustain, max earn, equity, civility, coop, hold accountable). |
| 3. | **Proposal** | Proposing conservation strategies/other solutions (e.g., enforcement systems). |
| 4. | **Choose +/-** | Voicing acceptance/rejection ("choice") among proposals ("choosing"). |
| 5. | **Eco Info** | Discuss ecological information (e.g., token/resource system dynamics). |
| 6. | **$ Info** | Discuss economics of the dilemma (e.g., econ processes, dynamics, economy); potential economic consequences of decisions/strategies/agreements. |
| 7. | **Soc Info** | Discuss own/others' motivations/drivers of behavior; beliefs/perceptions relevant to social and behavioral processes or dynamics. |
| 8. | **Rule Info** | Clarifying a proposed or existing agreement or strategy. |
| 9. | **Exp Info** | Discuss factual information about the experiment (e.g., # rounds, time left). |
| 10. | **Mon Res** | Monitoring (asking, observing, reporting) resource pool levels. |
| 11. | **Mon Earn** | Monitoring player or total earnings. |
| 12. | **Mon Beh** | Monitoring a player action (without judging/evaluating or enforcing). |
| 13. | **Pose Harm** | Voice a perceived harm or injustice (e.g., stolen tokens, abusive/unfair sanction). |
| 14. | **Pose Concern** | Voice a perceived concern about a proposal or existing agreement/strategy. |
| 15. | **RJ Apology** | Apologize for a perceived harm. |
| 16. | **RJ Atone** | Provide/agree to tangible atonement for perceived harm (e.g., compensation).[1] |
| 17. | **RJ Justify** | State a rationale (info type) to justify a perceived harm (e.g., *RJ Justify: Soc Info*). |
| 18. | **RJ -** | Any deliberate refusal to apologize, atone, or justify for a perceived harm. |
| 19. | **RJ Forgive +/-** | Forgive or absolve[2] a perceived harm; accept apology/atone/justify. |
| 20. | **Coord** | Non-constitutional real-time implementation of existing strategy (e.g., calling out the start time for harvest based on an existing agreement); or non-constitutional decision to address immediate circumstance (e.g., share stray token). |
| 21. | **ENF Ack** | Report own/other's cooperation or intent to cooperate. |
| 22. | **ENF Praise** | Praise past cooperation or encourage future cooperation. |
| 23. | **ENF Ask** | Ask for cooperation. |
| 24. | **ENF Tell** | Tell others to cooperate. |
| 25. | **ENF Shame** | Shame or guilt others for potential/actual non-cooperation. |
| 26. | **ENF Warn** | Warn of potential/actual consequences for non-cooperation. |
| 27. | **ENF Threaten** | Harshly warn or curse at an individual(s) for potential/actual non-cooperation. |
| 28. | **ENF Punish** | Warn/threaten with evidence of punishment (e.g., monetary penalty). |
| 29. | **Defect** | Active refusal to obey/cooperate with existing agreements, including taunts. |
| 30. | **Small Talk** | Discussing non-task topics (e.g., popular events, personal interests). |
| 31. | **Humor** | Deliberate attempts to be silly or funny. |
| 32. | **Polite +/-** | Deliberate attempts to be im/polite (e.g., thanking; interrupting on purpose). |
| 33. | **Input -** | Actively discouraging other's input into constitutional/operational decisions. |
| 34. | **Ambiguous** | Too ambiguous to code reliably. |
| 35. | **Uncodeable** | Statement is incomprehensible (e.g., illegible), or no category exists. |

Note: Generally speaking (unless context strongly suggests otherwise), proposals and enforcement codes trump (over-ride) lower-order codes (e.g., goals, info codes, politeness, humor).

[1] In addition to other forms of compensation for perceived harms, this includes sharing/exchanging some of one's own tokens to compensate for perceived (b) "stealing" or taking of another player's tokens or (c) counteracting inequities among players (e.g., giving one's own tokens to a player to make final harvests more equal).
[2] Absolve = declare free from guilt or blame.

# CHAPTER 2: DATA PREPARATION

## 2.0 INTRODUCTION

Follow these steps to prepare the raw communication data for coding. Before coding can occur, each group's raw communication (chat) data must be organized into complete thought units, and then segmented and labeled into major events, such as decision events.

## 2.1 UNITS OF ANALYSIS

We distinguish between two units of analyses: thought units (parsed into their component phrases) and events (cf. Reed, 2018 for general discussion of units of analysis).

### 2.1.1 For Coding Topical Content

The basic unit of analysis for topical content is stand-alone **thoughts ("thought units")**.

***Simple Thought Units:***

When communication is simple, a thought unit is typically synonymous with individual or discrete text lines in the computer-mediated chat. However, sometimes the components of a single thought will be broken up or spread across multiple lines: this is often due to the nature of communicating by written chat (participants cannot follow typical facial and verbal cues to pace their conversations). Participants may also intentionally divide a single thought across multiple lines (for clarity), or their communication may be accidentally interrupted by other participants as individuals communicate in parallel. In these cases, the components of the single thought unit will need to be combined to complete the intended statement.

***Complex Thought Units:***

Individuals sometimes communicate complex ideas (thought units) consisting of multiple, related sub-thoughts (e.g., an ecological rationale provided with a proposal for a conservation strategy). The **component phrases (i.e., sub-thoughts)** that make up the larger, more complex thought must be parsed, so that codes can be applied to those sub-thoughts: e.g., phrase 1 (*Proposal*), phrase 2 (*RAT: Eco Info*).

**Hence, when coding topical content, stand-alone simple thought units and the sub-thoughts (phrases) of more complex though units are the units of analysis.**

### 2.1.2 For Functional Coding

The basic unit of analysis for functional coding (e.g., of democratic decision making) are **stand-alone events** (e.g., coding within decision events).

## 2.2 SEGMENTING

Before coding can begin, the raw communication data (line-by-line statements) must be combined into (a) the highest-order, stand-alone thought units, (b) organized, (c) segmented into major events, and then (d) complex thought units must be further parsed into their component sub-thoughts (i.e., component thought units).

It is **highly recommended that your coding team work together closely** throughout this step of the coding process, to ensure that multiple perspectives are brought to bear on identifying and properly consolidating thought units, as well as identifying and segmenting events and sequences of events (cf. Ratajczyk et al., 2016). **Consensus here is essential**, because this step is foundational to all other coding, greatly influencing subsequent steps. For this process, ensure that at least one highly experienced coder (e.g., lead investigator), with deep conceptual knowledge of social dilemmas, governance, and the specific experiment (e.g., particular resource dilemma) supervises the process. Their knowledge is often important for disambiguating dialogue that may otherwise be ambiguous to less experienced coders.

Finally**, an important word about context**: when coders agree on the context surrounding a particular passage of text (chat), they will more frequently agree on the code(s) to apply. Thus, during this initial consolidation and segmentation process, it is very important to keep in mind the history of the group and the specific local context of the particular line of communication being considered at a given time: context changes everything in this coding system. Identifying context and proper segmentation is often an iterative process.

You will need to revisit and update the segmentation as new light is shed on a particular group's dialogue, as you gain more experience with that particular group and the other groups within the experiment. This process can take a few weeks.

### 2.2.1 Step 1: Combine Broken Thought Units

The first step of data preparation is to **identify broken thought units and combine them**. You may need to reorder lines. ***Note combined statements by including the timestamp immediately before the combined part.***

Example

*Original*

| | | |
|---|---|---|
| **-332.277** | **4** | **ok everyone** |
| -331.921 | 2 | Okay number 4 |
| ***-326.141*** | ***3*** | ***eat the tokens more slowly*** |
| **-323.588** | **4** | **how bout this** |
| -320.191 | 2 | I have some non physical threat choice words for you |
| ***-319.037*** | ***3*** | ***that way we all get more*** |

*Consolidated*

| | | |
|---|---|---|
| -332.277 | 4 | **ok everyone** |
| **-323.588** | **4** | **how bout this** |
| -331.921 | 2 | Okay number 4 |
| -320.191 | 2 | I have some non physical threat choice words for you |
| -326.141 | **3** | **eat the tokens more slowly** |
| **-319.037** | | **that way we all get more** |

### 2.2.2 Step 2: Reorganize into Major Discussion Topics

Because of the nature of chat-based communication, group members may discuss multiple topics, or trigger multiple events, in parallel—intermingling the statements. Whenever possible, these separate discussion topics and events should be disentangled, and then consolidated and reorganized chronologically. Labeling these larger segments helps to further organize and conceptualize the topics, history, and local context of the discussion.

#### *A. Separating Parallel Dialogue into Distinct Segments*

For example, in Figure 2.1, Player 3 and 4 greet everyone. Then, Player 2 raises the topic of using monetary penalties (economic sanctions) at timestamp -402.971. While Players 3, 4, and 2 continue to discuss that topic, Player 1 interjects a new topic (proposal to use a waiting rule to allow tokens to regenerate) at timestamp -343.100. Player 2 then briefly responds with a third topic (proposal to use a private property rule) at timestamp -324.586.

**Figure 2.1** Example: Separating Parallel Dialogue into Distinct Segments

| Timestamp | Player | |
|---|---|---|
| -414.456 | 3 | Howdy yall |
| -409.577 | 4 | whats up |
| **-402.971** | **2** | **Hey, so do yall want to use these penalties?** |
| -361.503 | 3 | I don't want to but it depends of u guys want to set up any other rules |
| -361.16 | 4 | idk its up to yall it doesnt matter to me what we do |
| -359.119 | 2 | I kinda feel like they're pointless if we both lose money in the end |
| **-343.1** | **1** | **Please note: The pieces do regenerate, if we wait perhaps for the first minute all of us will benefit more from making sure we don't attain all of the pieces.** |
| -334.644 | 2 | TRUE |
| **-324.586** | **2** | **we can all do a corner?** |
| -298.595 | 4 | yeah im cool with that |
| -296.359 | 3 | thats a good idea |
| -273.24 | 4 | so we both dont lose money bc i think that is stupid |
| -261.391 | 3 | I agree |
| -246.746 | 2 | exactly. I thought i was the only one who thought that |
| -233.518 | 3 | But what if someone breaks the rules |
| -221.767 | 3 | what ab instead of using the penalty if someone breaks the rules |
| -218.233 | 4 | then you a fake |
| -214.706 | 2 | Lol |
| -197.703 | 3 | ok cool i trust yall |
| -194.51 | 4 | just stay in your corner |
| -185.818 | 1 | That works for me. I strongly suggest we wait and let pieces regenerate before trying to take them all. The more pieces we can potentiall obtain, the better. [illegible] |
| -172.072 | 4 | if not you can penalize them |
| -162.639 | 4 | i agree 1 |
| -158.652 | 3 | uWu |
| -152.666 | 2 | So if we dont click on all of them they will regenerate over time? |

These discussion topics, which also constitute decision events (see discussion further below), will need to be disentangled, consolidated, and reorganized chronologically to facilitate conceptual understanding, and coding (see Figure 2.2; next page)

**Figure 2.2** Example: Reorganizing and Labeling Topical Segments

| Timestamp | Player | |
|---|---|---|
| -414.456 | 3 | Howdy yall |
| -409.577 | 4 | whats up |
| ***Constitutional Decision 1: Penalties*** | | |
| -402.971 | 2 | Hey, so do yall want to use these penalties? |
| -361.503 | 3 | I don't want to but it depends of u guys want to set up any other rules |
| -361.16 | 4 | idk its up to yall it doesnt matter to me what we do |
| -359.119 | 2 | I kinda feel like they're pointless if we both lose money in the end |
| -298.595 | 4 | yeah im cool with that |
| -273.24 | 4 | so we both dont lose money bc i think that is stupid |
| -261.391 | 3 | I agree |
| -246.746 | 2 | exactly. I thought i was the only one who thought that |
| -233.518 | 3 | But what if someone breaks the rules |
| -221.767 | 3 | what ab instead of using the penalty if someone breaks the rules |
| -218.233 | 4 | then you a fake |
| -214.706 | 2 | Lol |
| -197.703 | 3 | ok cool i trust yall |
| -194.51 | 4 | just stay in your corner |
| -172.072 | 4 | if not you can penalize them |
| -185.818 | 1 | Also, the penalties seem quite pointless yes.. there are no personal gains. |
| ***Constitutional Decision 2: Harvest Strategy (Wait for Regen)*** | | |
| -343.1 | 1 | Please note: The pieces do regenerate, if we wait perhaps for the first minute all of us will benefit more from making sure we don't attain all of the pieces. |
| -334.644 | 2 | TRUE |
| -185.818 | 1 | That works for me. I strongly suggest we wait and let pieces regenerate before trying to take them all. The more pieces we can potentiall obtain, the better. |
| -162.639 | 4 | i agree 1 |
| -158.652 | 3 | uWu |
| -152.666 | 2 | So if we dont click on all of them they will regenerate over time? |
| -144.261 | 1 | yes |
| -140.986 | 2 | Okay, cool |
| -133.054 | 1 | we gain more by waiting |
| -114.418 | 2 | I wondered why some would pop back up lol I see now |
| -92.705 | 1 | The more there are on the screen the faster they reappear. |
| ***Constitutional Decision 3: Private Property*** | | |
| -324.586 | 2 | we can all do a corner? |

### *B. Recognizing "Grand" Segments*

Whenever possible, divide the stream of dialogue into smaller discussion topics and events. However, sometimes groups will discuss multiple topics/decision events as a singular, grand, multi-dimensional discussion topic or decision event. These are identified when participants themselves actively combine the elements into singular, complex proposals instead of addressing each of the components sequentially or separately. For example, when discussing two conservation strategies (e.g., private property, waiting rule) in parallel, participants may clearly combine the elements into a single proposal (i.e., private property with a waiting rule). In such cases, it is very difficult (perhaps impossible) to disentangle the two elements as two separate discussion topics or decision events. Such cases should therefore be treated as a single segment (e.g., decision event), for functional coding purposes.

For example, in Figure 2.3, the group discusses the use of monetary penalties (economic sanctions) and a paced harvesting rule as a grand, combined strategy. Player 2's statement at timestamp -256.069 (and the discussion that follows) clearly indicates that the group is treating these elements as a combined, singular proposal (not two separate proposals). Therefore, this sequence of dialogue is treated as a single (grand) segment.

**Figure 2.3** Example: A Grand Decision Event

| Timestamp | Player | |
|---|---|---|
| ***Constitutional Decision 1: Penalties & Harvest Strategy (Regenerate)*** | | |
| -414.416 | 4 | No monetary penalties |
| -405.769 | 1 | I agree |
| -385.41 | 3 | we need to try and regenerate our tokens |
| -384.848 | 4 | And we should stop taking all the coins the more that regovanate the more tokens we all get |
| -374.126 | 4 | I agree |
| -372.193 | 2 | I think instead of going straight for all the tokens we should let them build up, take some, then let them regenerate again |
| -361.641 | 3 | i agree with 2 |
| -355.402 | 4 | same |
| -354.199 | 1 | sounds good |
| **-256.069** | **2** | **So is everyone on the same page here? No monetary penalties and then allowing our tokens to regenerate** |
| -249.169 | 1 | yes |
| -245.176 | 4 | tes |
| -240.476 | 4 | yes* |

Grand segments are also common after groups revisit social contracts, after having decided on the elements separately, during previous discussions. For example, during the first communication round, the group illustrated below (Figure 2.4) decided how to use monetary penalties and created a harvesting strategy in three distinct decision events. After they tested these elements of their strategy, they revisited the institutional arrangements during the second round of communication as a singular, grand decision. The decision event starts when Player 4 asks if the other players have any concerns with any elements of the overall strategy. Player 3's statement at timestamp -149.654 confirms that this decision is treated as a singular, grand decision about a complex institutional arrangement composed of multiple elements combined and considered all at once as a single package.

**Figure 2.4** Example: Reconsidering Several Institutional Arrangements (Decided Earlier During Separate Decision Events) as A Single Grand Decision Event Later

| Timestamp | Player | |
|---|---|---|
| ***Constitutional Decision 4: Revision of Harvest Strategy (Wait Rule/Lone Tokens/Regen/Free for All )*** | | |
| **-379.42** | **4** | **any concerns?** |
| -349.849 | 1 | I think we shouldnt clean the edges off. Try to leave groups there to and take ines that are in between two others |
| -334.672 | 3 | how about the at 60 sec we just stop and let them grow and the last 20-30 sec do the mad dash |
| -296.266 | 4 | I like cleaning the edges so that we can try to get it to grow in the center. The more centralized, the faster it will grow |
| -296.243 | 1 | Im okay with that part too. Just give it a regen time. Id be down to go at 25-30 secs left |
| -283.599 | 2 | and giving them a few seconds at the beginning to fill in a few more spots |
| -280.114 | 4 | And i like 3s idea |
| -272.07 | 4 | agreed 2 |
| -257.347 | 1 | But the ones in the middle will grow at the same rate as long as you just make sure theyre surrounded still |
| -249.726 | 2 | give time for clusters to form |
| -233.483 | 3 | so how long in the beginning |
| -223.449 | 4 | true. I'm down for that 1 |
| -214.218 | 4 | and id say about 30 seconds in the beginning |
| -218.065 | 1 | So I think by not clearing the edge its just more for everybody to farm and let regen |
| -176.071 | 1 | Okay |
| **-149.654** | **3** | **okay so first 30 secs let grow then just go after lone stars then at the last 60 secs let grow and at 25 secs everyone go at it** |
| -142.955 | 2 | yeah that would give us 2 1/2 minutes to collect until the next hold period and then the mad dash |
| -117.284 | 1 | Yeah and in the 2 and a half farm lonestars and middle of groups |
| -106.082 | 2 | sounds good |
| -89.048 | 4 | awesome. |

### *C. Line Numbers*

After you have identified the segments and reorganized the lines within each segment logically and chronologically, you should number each line. This aids in team discussion and the systematic tracking of particular coded statements.

### 2.2.3 Step 3: Identify Decision Events

Many of the segments you have identified during Step 2, are going to be decision events. During Step 3, your goal is to **identify all major decision events made by the group. Label each event, and record the line numbers for ease of identification. This step finalizes the basic unit of analysis for assessments involving decision events (e.g., calculation of the *Democratic Decision-Making Index* to assess Ostrom's design principle for inclusive and procedurally fair collective choice, i.e., shared decision-making).**

**Decision Events** refer to segments of dialogue in which group members introduce, discuss, modify, and ultimately choose from among various proposals. They typically begin with a proposal (e.g., proposed conservation or enforcement strategy, "Proposal"), perceived concern or injustice ("Pose Concern," "Pose Harm"), or other issue, and end when the group transitions to another topic, runs out of time (i.e., the dedicated chat period ends or round ends), or clearly chooses an option. You must rely on context to determine the end point.

***A) Consolidating Broken Decision Events into One Event***

As implied by the previous instructions on consolidating broken dialogue, a decision event may be spread (broken) across multiple speaking turns, thought units, or segments of dialogue *within the same chat period or round*. A particular decision event can be interrupted or set aside momentarily by the group and then completed during the same chat period or round.[3]

For example, as illustrated in Figure 2.5, the following group began the first chat period with a decision event to decide their resource management strategy (Decision 1: Lines 1-7). This was interrupted by a restorative justice event (see *Step 4: Identify Restorative Justice Events*) to determine how to deal with a perceived injustice (stolen tokens) raise by Player 2 (Restorative 1: Lines 8-9). Afterward, the group returned to Decision 1 to further add to the proposed conservation strategy (Line 10). That was again set aside to deal with another perceived injustice (stolen tokens) raised by Player 3 (Restorative 2: Lines 11-16). The group then returned to Decision 1 and finalized it by choosing a private property rule (Lines 17-21).

*Note:*

*The broken parts of Decision Event 1 in this example will be reorganized (moved and consolidated) in Step 2. They have not been reorganized here, so that they can serve as an example of identifying broken parts of a decision event from an original dialogue sequence.*

---

[3] By default, we follow the convention that a single decision event cannot be carried across multiple chat periods or rounds, unless the context of the dialogue clearly indicates that participants are treating the situation as a single decision that happened to be interrupted midway and is continuing. This is most common when group members are discussing a decision (a) at the end of the chat period (in which case the decision may be continued into the beginning of the harvest round) or (b) the end of a harvest round (in which case the decision may be continued into the beginning of the next chat period).

**Figure 2.5** Example: Reconsidering Several Institutional Arrangements (Decided Earlier During Separate Decision Events) as A Single Grand Decision Event Later

| Time Stamp | Player | |
|---|---|---|
| ***Constitutional Decision 1: Resource Management Strategy (Private Property)*** | | |
| -332.277 | 4 | ok everyone |
| -323.588 | | how bout this |
| -331.921 | 2 | Okay number 4 |
| -314.386 | 4 | the whole grid is a 13x13 square. |
| -306.146 | | i mean its 4 13x13 squares. |
| -298.912 | 4 | we all get our own quadrant |
| -326.141 | 3 | eat the tokens more slowly |
| -319.037 | | that way we all get more |
| -298.22 | 2 | lets all get our own section |
| -282.123 | 3 | stay in your section and take the tokens slowly over the whole period |
| ***Restorative Justice 1: Compensating Player 2 (for "stolen" tokens)*** | | |
| -320.191 | 2 | I have some non physical threat choice words for you |
| -291.178 | 4 | #2 you can have 4 of my tokens. |
| -287.12 | | fairs fair. |
| ***(Returning to Decision 1)*** | | |
| -282.118 | 4 | *1 gets top left* |
| -279.727 | | *2 gets top right* |
| -274.437 | | *3 gets bottom left* |
| -271.255 | | *4 gets bottom right* |
| ***Restorative Justice 2: Compensating Player 3 (for "stolen" tokens)*** | | |
| -271.741 | 3 | also y'all took my tokens i had |
| -267.466 | 2 | yea you took 3's token |
| -252.577 | 2 | 3 gets 4 of your tokens i dont need it |
| -247.258 | 3 | thx 2 <3 |
| -244.412 | 4 | oh fine 3 you get them |
| -237.949 | 3 | thx |
| ***(Returning to Decision 1: End Decision Event 1)*** | | |
| -240.721 | 4 | but everyone got that? |
| -233.277 | | with the whole we get our own quadrant thing? |
| -235.366 | 1 | yeah |
| -233.916 | 2 | Yea yea |
| -229.977 | 4 | good |

### *B) Reconsidering/Reaffirming A Prior Decision (Triggers A New Decision Event)*

Sometimes groups reconsider a previously concluded decision (e.g., after testing a conservation strategy, or when new information/insights emerge): this may be to (a) re-affirm the existing social contract without changes or (b) introduce amendments to the prior social contract, with substantive changes. Either way, these segments should be considered *new* decision events, because they reopen the social contract to collective consideration and choice.

For example, in the previous illustration (Figure 2.5), when the group revisited Decision 1 (see figure above), the context of the discussion indicated that the group intended to continue the ongoing dialogue about private property, and that a final decision had not been reached until the segment labeled "End of Decision Event," which is when Player 4 asked if everyone was in agreement about the private property proposal, and Players 1 and 2 confirmed. Hence, those segments were not considered new/separate stand-alone decision events. In contrast, Figure 2.6 illustrates an example in which the group finalizes a private property rule decision during Chat Period 1 (at Line 24), tests that strategy during Round 4, and then later reopens the institutional arrangement for reconsideration and decision: during Chat Period 2, Players 2 and 3 ask if anything should be done differently (Lines 39-40), reopening their previous decision for revision/re-approval, triggering a new decision event.

**Figure 2.6** Example: New Decision Event Triggered by Reconsidering a Previously Concluded Decision

| | | | | | |
|---|---|---|---|---|---|
| | **GROUP 3** | | | | |
| | | | | | |
| | | **Chat 1 (Rd4)** | | | |
| | ***Sharing Information*** | | | | |
| 1 | -331.431 | 3 | :) | | |
| 2 | -322.86 | 4 | hello | | |
| 3 | -316.592 | 1 | Ok so im a bit confused how the token system works | | |
| 4 | -297.479 | 3 | Are we suppose to let other people get a chance at collecting tokens?? | | |
| 5 | -296.665 | 1 | Do they need to be in a group to regenerate? | | |
| 6 | -270.399 | 2 | We need to let them repopulate more | | |
| 7 | -269.806 | 1 | Gotcha | | |
| 8 | -248.932 | 3 | Do we have to all stick together? | | |
| 9 | -246.48 | 2 | The more around in one space the faster they will regenerate | | |
| | | | -238.432 | but if we take them all out then they wont come back | |
| 10 | -226.842 | 4 | We can seperate and kinda farm" our area" | | |
| 11 | -216.102 | 2 | I have a feeling that we are suppose to somehow work as a group | | |
| | | | -200.514 | But that doesnt make sense because we obviously all want the money lol | |
| 12 | -186 | 1 | its pretty much a way of testing how long we can work as a team before we eventually just go every man for himself | | |
| | | | | | |
| | ***Decision 1: Private Property*** | | | | |
| 13 | -188.616 | 3 | How about we each pick a corner? | | |
| 14 | -164.311 | 1 | But yea pick a corner. i agree | | |
| 15 | -158.65 | 2 | I like that | | |
| 16 | -149.166 | 3 | I'll take the top right cone | -142.416 | *corner |
| 17 | -139.383 | 4 | We all start in a different corner | | |
| 18 | -132.409 | 1 | bottom left | | |
| 19 | -129.767 | 2 | I agree too | | |
| 20 | -124.028 | 4 | okay, i guess im bottom right | | |
| 21 | -105.394 | 2 | Ill take top left corner | | |
| 22 | -97.952 | 1 | Just do that for this round. we can switch it up next time | | |
| 23 | -62.866 | 2 | Wait if we each start in a corner like #4 said, then should we stick in that corner? | | |
| 24 | -56.944 | 1 | go to the corner you picked for this round | | |
| | | | | | |
| | ***Sharing Information*** | | | | |
| 25 | -51.813 | 3 | Anyone over $10 yet? | | |
| 26 | -45.858 | 2 | Jk | | |
| 27 | -45.459 | 1 | not me | | |
| 28 | -41.013 | 4 | no | | |
| 29 | -12.263 | 2 | nope | | |
| | | | | | |
| | **During Rd 4** | | | | |
| 30 | 230.48 | 1 | Id say this is going well so far | | |
| 31 | 237.849 | 2 | Before it ends collect them all | | |
| | | | | | |
| | | **Chat 2 (Rd5)** | | | |
| 32 | -310.347 | 1 | So what do we think? | | |
| 33 | -307.28 | 4 | that worked pretty well | | |
| 34 | -304.583 | 3 | I was too greedy at the sta | -287.959 | They didn't regenerate fast |
| 35 | -249.782 | 2 | The regenerated in spurts | | |
| 36 | -242.101 | 1 | Try to keep clusters of them for as long as you can and eat" the ones that are sitting on the outside of cluster" | | |
| 37 | -218.012 | 3 | Same. | | |
| 38 | -213.838 | 2 | Once one persons would regemerate, then everyone elses would. | | |
| | | | Whoever said it's testing us on how long we last with team work is right | | |
| | | | | | |
| ***Decision 2 : (Reopening Prior Decision) Reaffirming Properties*** | | | | | |
| 39 | -264.61 | 2 | So we didnt really start out in different corners so I think sticking to corners is a good idea | | |
| 40 | -246.629 | 3 | Everyone okay with the corners they picked? | | |
| 41 | -210.535 | 1 | Alright then. Pick em | | |
| 42 | -202.647 | 3 | Top right | | |
| 43 | -202.604 | 4 | bottom right | | |
| 44 | -191.934 | 2 | Im okay with sticking to the corners we just had | | |
| 45 | -190.67 | 1 | Bottom left for me then | | |
| 46 | -176.246 | 2 | Top left | | |
| 47 | -165.148 | 4 | I know i did | | |

### *D. Types of Decision Events*

We differentiate two fundamental types of decision events, and at least two major (i.e., default) decision topics, which are helpful to identify for functional coding. Groups can make **constitutional decisions**, in which they deliberate and choose fundamental social contracts to govern the social-ecological dilemma, or they may make **operational decisions**, in which they deliberate and choose courses of action in the real-time (or near real-time) implementation or coordination of their constitutional social contracts, without fundamentally or substantively altering those underlying social contracts. The most common decision topics pertain to deciding conservation strategies and agreements or enforcement systems.

#### *Identifying Constitutional Decisions*

In the current coding system (Version 2), constitutional decisions are given primacy. Thus, each constitutional decision is carefully identified, labeled, and functionally coded. To identify whether a decision event is a constitutional decision, the coder must use group history and the context of the current dialogue to determine whether the decision is about introducing or substantively altering fundamental social contracts (e.g., conservation agreements). Given the nature of the experimental environment (social-ecological dilemma) used to create this coding system, constitutional decisions will typically occur during dedicated chat periods, when group members have ample time to discuss without time pressure—not during actual harvesting periods (i.e., rounds). Operational decisions typically occur during actual harvesting periods.

Potential constitutional decisions that arise during actual harvesting periods (rounds) are not identified and coded as such unless it is clear that a constitutional decision has occurred – this is most notably when players stop what they are doing in the round (or after harvesting every token that round), specifically to discuss a change to the existing social contracts. We have this caveat, because communication during a round is often incomplete, with incomplete statements and fewer players participating, because the harvest is ongoing in real-time making more comprehensive discussion impractical.

Constitutional decisions may be about anything fundamentally determining the group social contract(s). Typically, these pertain to creation/modification of conservation agreements and enforcement systems (i.e., governance).

#### *Identifying Operational Decisions*

By default, apparent “decisions” or actions taken during an actual harvest period (i.e., round) are considered to be operational decisions – decisions meant to implement pre-existing social contracts (e.g., conservation agreements). To identify whether a decision event is an operational decision, the coder must use group history and the context of the current dialogue to determine whether the decision is fundamentally about the basic coordination to implement an existing social contract. In the context of this experimental environment (social-ecological dilemma), such events typically occurring during harvest periods (i.e., round), in real-time, as players discuss tangible ecological and social/behavioral conditions necessary to coordinate behavior around a particular strategy. A common example is when group members, or a particular player, monitors and announces the passage of particular timestamps to signal when harvesting may begin or end, based on a previous, constitutionally-determined conservation agreement (e.g., announcing the 45-second mark for a group that agreed to start harvesting after 45 seconds has passed at the beginning of the round).

Sometimes, individuals will propose minor changes to previous constitutional decisions in response to immediate environmental circumstances (e.g., deciding to stop harvesting in 40 seconds instead of 50 during the current round, because tokens are regenerating slower than anticipated based on the previous round). By default, we do not consider these to be constitutional decisions, because they do not substantially alter the social contract. However, group members may consider otherwise; if group members stop to deliberate the merits of the proposed operational adjustment, and substantive deliberation ensues, then the decision may be considered a constitutional decision. However, typically, group members themselves will be cognizant of the potential constitutional nature of such an exchange, and bring up the issue as a more direct, formal constitutional decision later, after the harvest round ends.

Though we do not label operational decision events in the formal coding, Figure 2.7 provides an example of both an operational decision and a constitutional decision occurring during a harvesting round, as a clarification. In this example, the group previously chose to implement a private property conservation strategy (Constitutional Decision 1 and 2: not shown). During the subsequent harvest round, Player 1 and 2 coordinate to quickly decide who should get a token that is located in an ambiguous location on the boundary between the two player's properties (Operational Decision). When the harvest round ends, Player 1, 2, and 3 use the remaining time to initiate a constitutional decision (Constitutional Decision 3), where they decide to wait before harvesting tokens in future rounds.

**Figure 2.7** Example: Operational and Constitutional Decisions Occurring During a Harvest Round

| **DURING HARVEST ROUND 4** | | | |
|---|---|---|---|
| **Line** | **Timestamp** | **Player** | |
| | ***Operational Decision 1: Conservation Agreement (Sharing Specific Tokens/Coordinating boundries of previous private property agreement)*** | | |
| 26 | 141.909 | 2 | hey 1 can i get that one |
| 27 | 160.278 | 1 | just one |
| 28 | 171.216 | 2 | thx |
| 29 | 176.286 | 1 | np |
| | | | |
| | ***Sharing Info (Eco Info/Proposal)*** | | |
| 30 | 198.079 | 4 | two and three, leave some in between the ones you collect and theyll regenerate more so you get more $$ |
| | | | |
| | ***Constitutional Decision 3: Conservation Agreement (propose and choose to wait for tokens to regenerate)*** | | |
| 31 | 203.406 | 1 | we have so much time so we should wait to get more |
| 32 | 221.634 | 3 | trueeee |
| 33 | 227.765 | 2 | true will do nxt time |

### 2.2.4 Step 4: Identify Restorative Justice Events

During Step 4, your goal is to identify restorative justice events. Label each event, and record the line numbers for ease of identification. This step finalizes the basic unit of analysis for assessments involving restorative justice (e.g., calculation of the *Restorative Justice Index*).

**Restorative justice events** occur <u>when a player claims (e.g., states)</u> a perceived harm or injustice (*POSE HARM*) has occurred, such as (a) social contract violations that harm or unfairly affect someone (e.g., when a player harms others by violating a conservation agreement), (b) unfair enforcement events[4] (i.e., when social or economic sanctions are used: *ENF* codes), and (c) inequities/unfairness in the group's institutional arrangements, cost/benefits of those arrangements, resource allocations, and/or procedural implementation. The onset of a

[4] See Sections 3.1.20 to 3.1.27 and 4.2 for discussion of identifying and segmenting enforcement events.

restorative event triggers potential for the harm/injustice to be resolved, restoring justice, or worsened, deepening the perceived harm/injustice.

Figure 2.8 a restorative justice event triggered by perceived harms caused by players using unjustified/unfair monetary penalties (sanctions).

**Figure 2.8** Example: Restorative Justice Event Triggered by Perceived Unfair Sanctions

| | | | | | | | |
|---|---|---|---|---|---|---|---|
| | ***Restorative Justice Event 1*** | | | | | | |
| 2 | -393.151 | 5d17 | 2 | Stop giving me penalties please | PROPOSAL | (POSE HARM) | |
| 3 | -292.118 | 66af. | 1 | also sorry for penalties i realize now that's kut less $$ for all of us | | | |
| | -283.944 | 66af. | 1 | just* | | | |
| | | | | *also sorry for penalties* | RJ APOLOGY | | |
| | | | | *i realize now that's just less $$ for all of us* | $ INFO | | |

Note:

Restorative justice events may be embedded within, be part of, or be central (focal), to a constitutional decision event (e.g., constitutional decisions to resolve perceived harms caused by existing enforcement systems). When a restorative justice event overlaps with a constitutional event, or other major segment(s), it is helpful to delineate the event by recording the line numbers specifically associated with the Restorative Justice Event.

The most common forms of restorative justice events observed in case studies and prior experiments pertain to rule violations and enforcement events (e.g., Ostrom, 1990; Tyler, 2006a). Therefore, researchers may wish to restrict their functional coding and analyses of restorative justice events specifically to these two major categories. Doing would facilitate researchers to study potential differences in conflict resolution surrounding conservation agreements (e.g., breaking an agreement) vs. enforcement (e.g., unfair use).

### 2.2.5 Step 4: Parse Complex Thought Units into Phrases (Sub-thoughts)

After identifying, segmenting, and labeling major topics and events, the final step (in what is likely to be an iterative process) is to parse complex thought units, which consist of multiple phrases (i.e., sub-thoughts), into their component parts. Alternatively, this step can be completed concurrently with Steps 3-4: we find that the process of segmenting and labelling major topics and events triggers recognition of complex thought units and their elements, which can be beneficial to the overall task.

The goal is to parse complex but related/nested ideas into meaningful chunks (e.g., proposals and their associated rationale(s)), not basic parsing into syntactic structures *per se*. Thus, sub-thoughts, such as multi-part proposals and their rationales, will be parsed (Figure 2.9).

**Figure 2.9**. Example: Parsing Complex Thoughts into Sub-Thoughts

| *Constitutional Decision 2: Conservation Agreement (propose and wait for token to regenerate before collecting)* | | | |
|---|---|---|---|
| **Line** | **Timestamp** | **Player** | |
| 16 | -343.1 | 1 | Please note: The pieces do regenerate, if we wait perhaps for the first minute all of us will benefit more from making sure we don't attain all of the pieces. |
| | | (Ph1)<br>(Ph2)<br>(Ph3) | *Please note: The pieces do regenerate*<br>*if we wait perhaps for the first minute*<br>*all of us will benefit more from making sure we don't attain all of the pieces.* |
| 17 | -334.644 | 2 | TRUE |
| 18 | -185.818 | 1 | That works for me. I strongly suggest we wait and let pieces regenerate before trying to take them all. The more pieces we can potentiall obtain, the better. Simply follow the guidelines, Allow pieces to regenerate. Obtain them overtime rather than all at once. |
| | | (Ph1)<br>(Ph2)<br>(Ph3) | *That works for me.*<br>*I strongly suggest we wait and let pieces regenerate before trying to take them all. Simply follow the guidelines, Allow pieces to regenerate. Obtain them overtime rather than all at*<br>*The more pieces we can potentiall obtain, the better.* |
| 19 | -162.639 | 4 | i agree 1 |

## CHAPTER 3: CODING TOPICAL CONTENT

### 3.0 INTRODUCTION

In this section each thought unit (or sub-thought) is coded, allowing the frequency (proportion) of different content to be calculated. Such root frequencies do not necessarily capture the full intent or function contained in dialogue, because they dissect larger segments of dialogue into fragments (Brauner, 2018). However, this coding serves as both a basic descriptor of communication patterns and commonly-tested potential correlate of cooperation (cf. Pavitt, 2011; Janssen, 2010).

**Unit of Analysis:**

Here, the unit of analysis the smallest thought unit:

- Simple, stand-alone thoughts
- Phrases (sub-thoughts) of more complex stand-alone thoughts

**Apply at least one code to each (a) simple thought and (b) phrase (sub-thought) of complex thoughts.**

**Coding Primary vs. Secondary/Tertiary Intent:**

Interpreting communication is inherently subjective. Moreover, a single statement can serve multiple purposes (Brauner 2018, Tschan et al. 2018).To best reflect the meaning of communicated ideas, and simplify the coding task to a more manageable scope, we strongly recommend that you focus on coding the ***primary content/intent*** of each thought unit, based on context, and **only code additional, secondary or tertiary meanings, if essential to properly understand/represent the most apparent intent(s)** of the statement.

A. **Most simple thoughts/phrases will receive only one code**, because they communicate only one thought or idea, or serve only one apparent purpose/intent**.**

B. **Some simple thoughts/phrases will receive multiple codes:** They may receive multiple codes when necessary, when such a phrase serves more than one function equally.

(1) This is **especially likely with choice** (i.e., choosing):

It is common for individuals to simultaneously affirm/confirm support (i.e., *CHOOSE+*) for a proposal while (a) adding to the proposal (i.e., *PROPOSE*) and/or providing a justification for the proposal (e.g., *RAT:SOC INFO*) within a single thought unit.

- Example 1:

  A group concludes that they should not use economic sanctions, unless someone clearly violates the conservation agreement, because both players lose money: Player 1 responds, "Also [indicating affirmation: *CHOOSE+*] the penalties seem quite pointless yes.. there are no personal gains [*RAT:SOC INFO*].

- Example 2:

  Player 1 says "Let's all get our own corner [*PROPOSE*]." Player 2 replies, "I'll take bottom left [*CHOOSE+, RULE INFO*]."

(2) It can also happen for **statement with double/triple meaning**:

Examples:

During the harvest round, Player 2 observes everyone overharvest and collapse the resource pool, then says "we messed up" [monitoring the resource: *MON RES*; sharing their perception and belief about others behavior: *SOC INFO*]

While chatting off-topic, Player 1 asks, "what are you going to use your money for?", which is small talk [*SMALL TALK*] asking for group members' goals/beliefs [*SOC INFO*], specifically regarding the currency (economy) of the experiment, which may influence behavior in the experiment [*EXP INFO*].

C. **Simple Replies:** Individuals sometimes respond to someone else's statements (thought units) with a simple reply or conversational convention, which indicates simple acknowledgement and/or agreement (e.g., repeating/reiterating what the other person said, saying "yeah" to confirm shared information, etc.). **Unless context suggests otherwise, code these replies using the same code as the original statement.**

Example:

Player 1, "do the tokens regenerate faster in clusters? [*ECO INFO*]"
Player 2, "yeah" [*ECO INFO*]

*We provide additional guidance below, in the individual code definitions, when needed to clarify common constraints or special considerations.*

## 3.1 CODING CATEGORIES AND DEFINITIONS

### 3.1.1 Greetings [*GREET*]

Thoughts/phrases where players greet one another (e.g., hi, hello, yo). Typically, these greetings occur at the beginning of chat periods, especially the first chat period.

### 3.1.2 Goal [*GOAL*]

Discussing goals: thoughts/phrases that introduce, ask, or discuss a goal (i.e., purpose, desired achievement, or aim). Goals specify aims or outcomes, not the specific steps, solutions, or methods of obtaining those aims/outcomes. Therefore, goals are *general* (e.g., let's make the tokens last longer [*GOAL:SUSTAIN*]), whereas proposals (see *Proposal*) are specific (e.g., let's go slower [*PROPOSE*] so we can make the tokens last longer [*GOAL:SUSTAIN*]). We identify five common goals: sustain, equity, civility, cooperation, and hold accountable. However, individuals/groups may discuss or introduce any kind of goal; therefore, coders may wish to include additional goal categories or use the generic "GOAL" code, without further designation to represent a general "other" goal category.

Common Goal Codes:

- **GOAL: SUSTAIN.** Discuss need/desire to make the tokens last longer, sustaining the resource (e.g., "let's make the tokens last longer")
- **GOAL: MAX EARN.** Discuss need/desire to make more, or the most, money possible.

- **GOAL: EQUITY.** Discuss need/desire to share the tokens/earnings more equally or "fairly" (e.g., equitably) (e.g., "Are we supposed to share the tokens?")
- **GOAL: CIVILITY.** Discuss need/desire to treat each other with civility (e.g., "Everyone, don't be rude," "Be nice to one another")
- **GOAL: COOP.** Discuss desire/need to work together (e.g., "We're not racing each other," "We need to work together") (cf. Shank et al., 2017)
- **GOAL: HOLD ACCOUNTABLE.** Discuss need/desire to hold each other accountable, discuss need or desire for an enforcement system (e.g., "we need some way to make sure everyone follows the rule")

### 3.1.3 Proposal [*PROPOSE*]:

Thoughts/phrases that introduce (i.e., propose) a new (or not yet resolved) solution to one of the major elements of resource governance (e.g., conservation agreements, enforcement). The solution must be a ***specific*** strategy, rule, or agreement to improve resource management, enforcement, or some other aspect of cooperation/governance (if it is not specific, then it is most likely a goal). Hence, the **solution must indicate tangible actions** for individuals to take.

**Special Considerations:**

**(1)** ***Proposals vs. Goals:***

Goals are more general than proposals: they do not specify specific actions for individuals to take. If a thought unit appears to be a proposal but is stated generally, then it may be a goal.

**(2)** ***Proposals and Goals:***

Frequently, but not always, individuals will state goals along with a proposal. In such cases, code both the proposal and goal(s).

**(3)** ***Proposals vs. Enforcement:***

Context (history) matters when identifying proposals and distinguishing them from enforcement, especially the enforcement category *ENF TELL*).

Thoughts units about courses of action individuals should take to manage the resource (or solve any other aspect of the social-ecological dilemma) **must be EITHER (a) novel or (b) not yet resolved by the group (i.e., ongoing deliberation about a strategy) to be considered a proposal**. In contrast, if a social contract (e.g., conservation agreement) has already been established by the group, then *thought units that may otherwise seem like a proposal are very likely a form of enforcement*: e.g., telling others to comply with the social contract [*ENF TELL*]*, or encouraging them to comply* [ENF *PRAISE*]*.*

Example:

The thought units below would be coded as proposals [*PROPOSE*], if the solutions discussed in the statements have not already been decided and established as social contracts by the group. However, the thought units could be coded as a form of enforcement [*ENF TELL*] if the social contracts are already established.

- "We all get our own quadrant"* [*referring to a private property rule*]
- "Eat the tokens more slowly"* [*referring to a slow harvesting rule*]

### 3.1.4 Choosing [*CHOOSE+/-*]

Thought units/phrases central to the act of choosing/selecting a course of action, during a decision event (i.e., in response to a proposal). Generally, *CHOOSE+* refers to the affirming or accepting a proposal, whereas *CHOOSE-* refers to rejecting a proposal.

**A) *CHOOSE+***

Affirmative or confirmatory thought units/phrases that indicate selection/support of a choice option (i.e., proposal), during a decision event. This is *not* the discussion of those courses of action: this is the actual *act of choosing* (e.g., votes cast).

Example:

- "But everyone got that? We get our own quadrant?" (Player 4) [*PROPOSE*]
  "Yeah" (Player 1) [*CHOOSE*]
  "Yea yea" (Player 2) [*CHOOSE*]
  "Sure" (Player 3) [*CHOOSE*]
  "Good" (Player 4) [*CHOOSE*]

**Special Considerations:**

**(1) *Implied Choosing:***

Sometimes an individual will not directly voice a choice but will instead move forward with a proposal, indicating assumed/implied choice.

Example

- Player 3: "Everyone take a corner next time." [PROPOSE]
  Player 4: "sounds good" [*CHOOSE*+]
  Player 2: "I'll take top left" [*RULE INFO*; implied *CHOOSE*+]

**(2) Common Co-occurring Codes:**

It is common for individuals to simultaneously affirm/confirm support (*CHOOSE*+) for a proposal (or reject it: *CHOOSE*-) while:

(a) <u>Adding their own proposal</u> to the original:

Example:

Player 1, "We should wait 30 seconds before we start harvesting" [*PROPOSE*]
Player 2, "We should also have our own corners" [*CHOOSE*+, *PROPOSE*]

(b) <u>Providing a justification</u> for the proposal:

Example:

Player 1, "We should wait 30 seconds before we start harvesting" [*PROPOSE*]
Player 2, "Yes, so more tokens will appear" [*CHOOSE+, ECO INFO*]

(b) <u>Clarifying Parameters of the Proposal</u> (especially with Private Property):

Example:

Player 1, "We should also get our own area" [*CHOOSE+, PROPOSE*]
Player 2, "I'll take bottom left" [*CHOOSE+, RULE INFO*]

**B) *CHOOSE-***

Clear and active disconfirmatory thought units/phrases of choice, indicating rejection of a particular choice option (i.e., proposal). This does NOT INCLUDE the mere absence of confirmation: therefore, failure to voice support for a proposal does NOT count as *CHOOSE-*.

Example:

- Player 2: “PLAN IS 20 SECOND TRUCE” [*PROPOSE*]
  Player 1: “That doesnt work” [*CHOOSE-*]
  Player 3: “no” [*CHOOSE-*]

**Special Considerations:**

**(1) *Counter-Proposals***:

Differentiating rejection of a proposal (CHOOSE-), acceptance (CHOOSE+), and proposals (PROPOSAL) can be difficult in situations where group members are brainstorming potential solutions (e.g., conservation strategies/agreements), because new proposals can essentially act or serve as a rejection (*CHOOSE-*) of the previous proposal(s).

Therefore, for clarity and simplicity, only use *CHOOSE-* when *PROPOSAL* would not apply (i.e., *PROPOSAL* typically trumps *CHOOSE-*). In addition, reserve CHOOSE- for instances where a group member explicitly rejects a prior proposal.

Example:

- Player 1: Let’s all get our own corners [*PROPOSAL*]
  Player 2: We should wait until the last minute to harvest [*PROPOSAL*] ***Not CHOOSE-***
  Player 3: No [*CHOOSE-*] let’s just take center of clusters! [*PROPOSAL*]

**(2) *CHOOSE- targets a conceptually prior PROPOSAL (or CHOOSE-) and does not automatically carry over to subsequent statements***:

Rejection of a proposal (*CHOOSE-*) targets a specific prior proposal (then resolves), which means you must keep track of specific proposals and their order within decision events to determine which proposal a person is rejecting. After the initial rejection (*CHOOSE-*) is resolved, if the deliberation (decision event) continues, it may be followed by subsequent proposals (*PROPOSAL*) and/or acceptance (*CHOOSE+*) of the same proposal by other players or acceptance of a different proposal. Thus, a prior *CHOOSE-* does not automatically carry over to subsequent replies or statements (i.e., guidelines regarding context and counter-proposals/proposals take effect).

Example:

*Context: a private property agreement has just been proposed.*

- Player 4: yeah [*CHOOSE+*] lets make a clear boundary or something that people are always able to clear off [PROPOSAL]
  Player 1: no [***CHOOSE-***] clearing tokens to make a boundary would only shorten restart time [ECO INFO]
  Player 2: yes [**CHOOSE+**, ECO INFO]

### 3.1.5 Ecological Information [*ECO INFO*]

Sharing, asking for, or discussing ecological information. Information-seeking (or information-providing) phrases that introduce, exchange, or ask for information about the nature of the resource system itself (regardless of accuracy). The statement is used/intended to provide information, or is seeking to understand something about, the ecology of the token resource system. This also includes sharing information about the effects of one's harvesting strategy on token ecology. Some common ecological topics: size of the field, scarcity, regeneration or regrowth, token locations and distributions, token density, resource pool dynamics.

Examples:

- "The whole grid is 4 13x13 squares" [*ECO INFO*]
- "Slow down [*PROPOSE*] the tokens replenish [*ECO INFO*]"
- Player 4: "1 how you collect so many. I tried the spacing out thing." [*ECO INFO*] Player 1: "that's all that i have been doing, just spacing and timing." [*ECO INFO*]

### 3.1.6 Economic Information [*$ INFO*]

Providing, asking, exchanging information about the (a) in-game economy or economics of the dilemma (e.g., economic dynamics, ecological-economic processes) or (b) anticipated or actual economic consequences of a particular course of action (e.g., decision, strategy, agreement). This includes appeals to players economic sensibilities (i.e., financial consequences).

Examples:

- "If we use fines we all lose money" [$ INFO]
- "a checkerboard pattern [PROPOSAL] gives you the most bank" [$ INFO]
- "How do we earn $30?" [$ INFO]

### 3.1.7 Social Information [*SOC INFO*]

Information-seeking (or information-providing) thought units/phrases that introduce, exchange, or ask for information about **(a) the social dynamics of the dilemma (i.e., *social* dilemma)** or **(b) people's beliefs and motivations that drive behavior** in the dilemma. This **includes discussing one's own beliefs/motivations, or other people's**. Finally, **(c) this includes beliefs about the purpose of the experiment and experimenter motivations**.

Common topics (thought units about):

(a) *Interdependency* (people's behaviors affect one another)
(b) *Self-Interest/Competition* (dilemma tempts people to be greedy/selfish, or compete)
(c) *Tragedy* (if people behave selfishly without limit then everyone will suffer)
(d) *Motivations* (motives, perceived reasons someone is behaving a particular way).

Examples:

- "If we say it's a free for all at 10 seconds [*PROPOSE*] that encourages people to finish early and start sharking" [*SOC INFO*] (*competition, motivation*)
- Player 2: "1 is just laughing at us now _-_" [*SOC INFO*] (*perceived reason for behavior*). Player 1: "I'm not" [*SOC INFO*] (*explaining one's own behavior*)
- "The experimenters are trying to make us turn on each other" [*SOC INFO*]
- "This study is fun: [*SOC INFO*] (*reveals player's subjective belief, motives)*

### 3.1.8 Rule Information [*RULE INFO*]

Providing, asking, exchanging (clarifying) information about an existing social contract (e.g., conservation agreement), or a proposed social contract:

Examples:

- "Did we say wait 30 seconds or 45 to harvest?" (*clarifying existing agreement*)
- "Is that your corner or mine?" (*clarifying private property rule boundaries*)
- "Wait what?" (*in response to a proposal*) (*clarifying an existing proposal*)

**Special Considerations:**

**(1)** ***Proposals vs. Rule Information:***

**Unless the context suggests otherwise, the first time a rule, strategy, or agreement is stated, that thought unit/phrase is typically coded as a proposal** (not rule information), because the intent of the statement is to inform the group of a new solution idea (potential social contract), not clarify an existing social contract or a solution idea that has already been posed to the group for consideration.

**After a proposal *has been accepted by the group*** restating the same rule, strategy, agreement gain is typically be considered rule information clarifying the existing social contract/rule (or possibly a form of Enforcement, depending on context).

**HOWEVER there are two common caveats:**

(1) Sometimes the same individual will restate their original proposal if others ignored the proposal, or if the individual is trying to insist/persuade the groups members to adopt the proposal: this often takes the form of repeating the same proposal over and over with no new details (i.e., no new information or change to the original proposal). In such a case, the statement will continue to be coded as a proposal (i.e., the *same* proposal).

(2) Sometimes, the individual will seek to clarify some aspect of the original proposal; typically, by adding some new information. Treat that as rule information *unless* the "new information" actually is adding a new component (amendment) to the original proposal: in that case, the *amendment* is treated as another (i.e. new) proposal.

*You must use the context (situational information, group history, knowledge of that person's typical communication patterns) to determine which code is most appropriate.*

EXAMPLE: DISTINGUISHING RULE INFO FROM A PROPOSAL

(Group 1)

The group first establishes a private property agreement, then clarifies it.

*Private Property Proposed*

| | | | | |
|---|---|---|---|---|
| 4 | -298.912 | 4 | we all get our own quadrant [*PROPOSE*] | |
| … | | | | |
| 10 | -282.118 | 4 | 1 gets top left | [*PROPOSE*] |
| | -279.727 | 4 | 2 gets top right | |
| | -274.437 | 4 | 3 gets bottom left | |
| | -271.255 | 4 | 4 gets bottom right | |
| … | | | | |

*Private Property Agreement Established*

| | | | |
|---|---|---|---|
| 17 | -240.721 | 4 | but everyone got that? |
| | -233.277 | | with the whole we get our own quadrant thing? [*PROPOSE*] |
| 18 | -235.366 | 1 | yeah [*CHOOSE*+] |
| 19 | -233.916 | 2 | Yea yea [*CHOOSE*+] |
| 20 | -229.977 | 4 | good [*CHOOSE*+] |
| 21 | -226.97 | 3 | sure [*CHOOSE*+] |

*Private Property Arrangements Clarified*

| | | | |
|---|---|---|---|
| 37 | -66.253 | 4 | **ok so to review [*RULE INFO*]** |
| | -63.653 | 4 | **1 is top left** |
| | -61.417 | 4 | **2 top right** |
| | -58.994 | 4 | **3 bottom left** |
| | -54.588 | 4 | **4 bottom right** |

**(2) *Social Information vs. Rule Info:***

*RULE INFO* differs from *SOC INFO* (see 3.1.7). *SOC INFO* asks clarification about motives, beliefs, social dynamics, or behavior. For example, if a player says the group should not do a free for all at the end of the round because it may encourage some players to collect their own tokens faster so they can take more tokens from the other Players during the free for all, then this would be considered sharing a belief about the drivers of behavior and social consequences of using a particular agreement (not clarifying the agreement itself).

### 3.1.9 Experiment Information [*EXP INFO*]

Information-seeking (or information-providing) thought units/phrases that introduce, exchange, or ask for **information about factual aspects of the experiment rules or design that are relevant to, and may therefore affect, group motivations and decisions**: for example, higher-order "rules of the game" (e.g., task instructions) and design constraints (e.g., number of rounds, length of rounds), which shape and constrain allowable actions and, therefore, motivations and behavior, in the focal social-ecological dilemma situation.

- Example 1:

  *While finalizing (choosing and specifying) a private property agreement, Player 4 interjects to let the group know that the experiment is designed such that each person starts in their own location on the screen, at the beginning of each round in the token task (thus providing information about the experiment that effects decisions within the game).*

| | |
|---|---|
| Player 3 | How about we each pick a corner? [*PROPOSE*] |
| Player 1 | But yea pick a corner. i agree [*CHOOSE*+] |
| Player 2 | I like that [*CHOOSE*+] |
| Player 3 | I'll take the top right coner. *corner [*RULE INFO*] |
| Player 4 | we all start in a different corner [*EXP INFO*] |

- Example 2:

  *After deciding on a conservation agreement, one group member (Player 2), says that they will now start making more money. Another player responds to this statement, thinking that Player 2 has violated one of the instructions of the experiment, which is that participants cannot make monetary deals outside the experiment. The group then discusses this, clarifying the experimental guidelines and in doing so clarifying and affecting the incentive structure, and motivations and decisions, within the group.*

| | |
|---|---|
| Player 2 | now, we'll make some real money [*SOC INFO*] |
| Player 4 | *illegal* [*EXP INFO*] |
| Player 2 | no its not [*EXP INFO*] |
| Player 2 | we'll farm the real tokens then lol [*EXP INFO*] |
| Player 4 | thats against the guidelines [*EXP INFO*] |
| Player 4 | and also i dont care fr [*SOC INFO*] |
| Player 2 | no, its not, theres no exchange of money outside experiment [*EXP INFO*] |
| Player 4 | shit u right [*EXP INFO*] |

**Special Considerations:**

**(1) *EXP Info vs. SOC Info:***

If participants (a) ask about the purpose of the experiment or its design elements/features, or (b) state their own subjective belief (interpretation) of the experiment or its design elements/features, then the proper code is *SOC INFO.* It is also *SOC INFO* if a participant asks, questions, or states the believe motives of the experimenter. In all these cases, the participant is not conveying *factual* information: they are conveying *subjective beliefs and communicate social information about themselves (i.e., their interpretations)*.

### 3.1.10 Monitor Resource [*MON RES*]:

Thought units/phrases where the purpose is to factually notice and report the status of the resource pool (tokens), WITHOUT judging or evaluating someone's behavior. *This category should be strictly distinguished from Enforcement (enforcement trumps monitoring).*[5]

Examples:

- "There's a lot of extra tokens" (*discussing leftover tokens*) [*MON RES*]
- "We have 2 tokens left." [*MON RES*]

### 3.1.11 Monitor Earnings [*MON EARN*]:

Thought units/phrases where the purpose is to factually observe or report the status of one's earnings, or someone else's earnings WITHOUT judging or evaluating someone's behavior. This includes keeping track of individual round, total individual, and collective earnings. If a phrase discusses potential economic consequences of an action/decision/strategy, then the phrase would be coded *$ INFO*.

Examples:

- "i got about $1.00 that time" [*MON EARN*]
- "how much have you all earned so far?" [*MON EARN*]
- "got $2 that time! [MON EARN] this new strategy pays better [*$ INFO*]

[5] ***Note: Enforcement Trumps Monitoring:*** If the statement seems to imply some kind of judgment or evaluation of someone's behavior, carries a moral/normative sentiment or tone, or is used in the same thought unit as enforcement (or in the context of other enforcement), then code the thought unit/phrase under-question as a form of Enforcement, not a form of monitoring.

### 3.1.12 Monitor Behavior [*MON BEH*]:

Thought units/phrases where the purpose is to factually observe or report someone's behavior, WITHOUT judging or evaluating that behavior. *This category should be strictly distinguished from Enforcement (enforcement trumps monitoring).*

Examples (but only if context implies that these are not actually enforcement):

- "Player 1 left their corner." (*In context of private property*)
- "Player 1 started a couple seconds early." (*In context of a delayed harvest*)

### 3.1.13 Perceived Harm [*POSE HARM*]

Thought units/phrases where a player raises a perceived harm/injustice for a particular individual or the group to consider, often triggering a Restorative Justice Event (or the potential for such an event). The perceived harm/injustice may have affected the player him/herself, another player, or a group of players.

For example:

- Player 3: "also y'all took my tokens i had" [*POSE HARM,* ENF SHAME]
- Player 4: "2 you made me really mad cuz u took all the tokens at first" [*POSE HARM, ENF SHAME*]

**Special Considerations:**

Unlike most other coding categories, POSE HARM can co-occur with enforcement, depending on context (i.e., enforcement codes do not necessarily trump pose harm). This caveat exists, because pose home is also the basis for identifying and functionally coding restorative justice events, processes, and outcomes.

### 3.1.14 Concern [*POSE CONCERN*]

Raising a *non-harm-related* (see *POSE HARM*) concern or problem for an individual or the group to consider. Frequently, these will pertain to concerns about the effectiveness of a conservation strategy and trigger a Decision Event to discuss a potential solution (e.g., modification of an existing strategy/agreement).

Example 1:

| | |
|---|---|
| Player 2: | "If there's 10 seconds left and you have a lot of coin thingys, I'll help you clear them out. ;)" [*PROPOSE*] |
| Player 2: | "yea?" [*SOC INFO {Implying: "what do you think?}*] |
| Player 4: | "i say stick to your own area no matter what" [*PROPOSE*] |
| Player 2 | "But then there's a lot extra." [POSE *CONCERN, MON RES*] |

Example 2:

| | |
|---|---|
| Player 2 | Hey, so do yall want to use these penalties? [*PROPOSE*] |
| Player 3 | I don't want to [*CHOOSE*-] |
| | but it depends of u guys want to set up any other rules [*SOC INFO*] |
| Player 4 | idk its up to yall [*CHOOSE*+] |

|  |  |
|---|---|
|  | it doesn't matter to me what we do [*SOC INFO*] |
| Player 2 | I kinda feel like they're pointless [SOC INFO, *CHOOSE+*] |
|  | if we both lose money in the end [*$ INFO*] |
| Player 4 | yeah im cool with that [*CHOOSE+*] |
| Player 4 | so we both dont lose money [$ INFO] bc i think that is stupid [*SOC INFO*] |
| Player 3 | I agree [*CHOOSE+, $ INFO, SOC INFO*] |
| Player 4 | exactly. I thought i was the only one who thought that [*SOC INFO*] |
| Player 3 | But what if someone breaks the rules [***POSE CONCERN***] |

**Special Considerations:**

***Pose Concern vs. Pose Harm, Goals, or Proposals***:

Unless context suggests otherwise, goals, proposals, and posed harms/injustice (*POSE HARM*) trump potential *POSE CONCERN* codes. Thus, if a potential concern could possibly be coded as a goal, proposal, or posed harm, then code it as such.

### 3.1.15 Apologize [*RJ APOLOGY*]

Player apologizes for a perceived harm/injustice (i.e., acknowledges fault and is remorseful).

Example:

Player 1: 2 you stole my token! [*POSE HARM*]
Player 2: Sorry 1 [*RJ APOLOGY*]
I didn't know that was yours. [*RJ JUSTIFY: SOC INFO*]

**Special Considerations:**

- ***Self-Reporting (by apologizing).*** *In order for this code to be applicable, there typically must first be an accusation or claim of harm/injustice (i.e., POSE HARM). However, sometimes a player self-reports a harms/injustice that they caused by apologizing before anyone else raises the concern.*

Example:

Player 1: Sorry player 2 [*RJ APOLOGY*]
Player 2: for what [*SOC INFO*]
Player 1: I accidentally [*RJ JUSTIFY*] took one of your tokens [*POSE HARM*]

### 3.1.16 Atone [*RJ ATONE*]

Player atones for a perceived harm/injustice by correcting the wrong or making amends: for example, by agreeing to allow a player to take some tokens from one's own available resource pool to make up for taking tokens from that player's pool.

Example:

Player 1: "2 you stole my token!" [*POSE HARM, ENF SHAME*]
Player 2: "1 you can take one of mine next time." [*RJ ATONE*]

***Note:*** *to be coded RJ Atone, typically there must first be a POSE HARM (that the RJ ATONE addresses), but not always (See RJ Apology section 3.1.15 for details).*

### 3.1.17 Justify [*RJ JUSTIFY*]

Player provides a rationale (explanation, justification) apparently intended to justify their behavior or attempts to explain the situation (i.e., root cause) of the problem/harm. This is a type of rationale (RAT in Codebook Version 1), so *RJ JUSTIFY* will co-occur with at least one other content code (e.g., *RJ JUSTIFY: ECO INFO*).

Example:

Player 1: “2 you stole my token!” [*POSE HARM, ENF SHAME*]
Player 2: “1 I had to because our boundaries weren’t clear” [*RJ JUSTIFY: RULE INFO*]

***Note:*** *to be coded RJ JUSTIFY, typically there must first be a POSE HARM (that the RJ JUSTIFY addresses), but not always (See RJ Apology section 3.1.15 for details).*

### 3.1.18 Refusal to Restore Justice [*RJ-*]

A player ***actively refuses to*** (a) apologize, (b) justify their behavior/explain the root cause of the problem/harm, or (c) atone/make amends.

Example:

- Player 1: “2 you stole my token!” [*POSE HARM, ENF SHAME*]
  Player 2: “who cares” [*RJ-*]

**Special Considerations:**

(1) **Omissions.** Merely failing to apologize, justify, or atone, **by saying nothing or not responding**, is **NOT coded as RJ-**, because the lack of information (i.e., lack of text to read) makes this situation too ambiguous in its intent to code reliably. For instance, we do not know if the person actively chose to ignore as an act of retaliation or if they simply did not notice that the harm/injustice (POSE HARM) had been stated.

Example:

- Player 1: “2 you stole my token!” [*POSE HARM, ENF SHAME*]
- Player 2: “who cares” [*RJ-*]

(2) **Why is *RJ-* the only negative category?** We distinguish 3 types of restorative justice (apologies, atonement, justification). But we do not distinguish different types for lack of restorative justice, because these often cannot be clearly identified. Specifically, it is possible to identify when someone has apologized, vs. made atonement, vs. justified a wrong (because it is a clear act). In contrast, it is often not possible to reliably identify when a person has refused to specifically apologize, vs. make atonement, vs. justify the wrong, because the absence of a specific act is more difficult to detect than the presence.[6]

### 3.1.18 Forgive [*RJ FORGIVE+/-*]

A player ***actively accepts (or does not accept) another player’s*** (a) apology, (b) justification for the perceived harm or injustice, or (c) their atonement.

[6] Fazio, R.H., Sherman, S.J. & Herr, P.M. (1982). The feature-positive effect in the self-perception process: does not doing matter as much as doing? *Journal of Personality and Social Psychology*, *42*(3), 404-411.

Example:

Player 1: “2 you stole my token!” [*POSE HARM, ENF SHAME*]
Player 2: “1 I had to because our boundaries weren’t clear” [*RJ JUSTIFY; RULE INFO*]
Player 1: “we good” [*FORGIVE+*]

OR

Player 1: “That’s a lie!” [*FORGIVE-*]

### 3.1.20 Coordinate [*COORD*]

Real-time coordination of an existing conservation/enforcement strategy (e.g., pacing/timing, property lines/tokens) during a harvest round, *without substantive/large changes/modifications to the existing strategy*. The intent is to implement the strategy, not enforce the strategy. You *must use context* to determine whether the intent is coordination or enforcement. OR a non-constitutional decision to address an immediate circumstance (e.g., share a stray token).

Examples:

- “you can go ahead and take the ones that are boxed in completely” [*COORD*]

  *Context:* Said during a harvest round, after the group previously decided to use a checkerboard/cluster harvesting strategy (conservation agreement).

- Player 2: “get ready to go nuts” [*COORD*]
  Player 1: “im finna go crazy” [*COORD*]

  *Context:* The group previously established an agreement to start the final harvest in the last minute of each round. During one of the rounds, Player 2 announced that the final harvest is about to start, so everyone could get ready.

- “Player 4 you can have the last one” [*COORD*]

  *Context:* No pre-existing conservation agreement, but Player 4 decides to share a stray token in the moment, during a round (limited coordination decision).

**Special Considerations:**

(1) ***COORD* is trumped by Constitutional Decision-making, RULE INFO, and enforcement.** If what first appears to be coordination turns out to (a) propose substantive changes to an existing strategy or (b) introduce a new strategy (e.g., conservation strategy), then that thought unit or phrase will be coded as a proposal. If the statement instead clarifies an existing conservation agreement (e.g., private property lines), then it will be coded as *RULE INFO*. And if context indicates that the intent is enforcement of an existing agreement, then it will be coded as *ENF* of the appropriate type. *COORD* is reserved for straightforward and constitutionally or informationally benign actions that do not substantively alter existing constitutional social contracts. *You must rely on context to determine what would be considered “substantially altering” existing contracts. This may differ by group. However, generally, a slight change (e.g., wait 45 seconds instead of 30) would not be considered substantial enough to trigger constitutional change, unless the group explicitly treats it as such (e.g., by stopping to discuss as a constitutional decision).*

For example, the excerpt below illustrates some discussion that occurred during a harvest round. There is both a constitutional decision and coordinating activities. In the constitutional decision (lines 29-31), Players 1, 2, and 4 decide to fundamentally change their social contract by introducing a private property arrangement. Prior to this point (during the previous dedicated chat period), the group decided that they would use an adaptive management approach, in which they harvest slowly from clustered tokens for the first two minutes of the round to cultivate more tokens. At lines 32-34, they coordinate the onset of the final harvest at the 2-minute (i.e., 120 second) mark, implementing the prior adaptive management agreement with no substantive constitutional change. From context, it is clear that this exchange is not intended to be an act of enforcement.

| | During Rd4 | | | | |
|---|---|---|---|---|---|
| 28 | 11.782 | 610: | 1 | they are generating | MON RES |
| 3 | ***Constitutional Decision 3: Conservation Agreement (private property)*** | | | | |
| 29 | 32.006 | 610: | 1 | do we want to each take a fourth? | PROPOSAL |
| 30 | 41.239 | c46( | 2 | yes | CHOOSE+ |
| 31 | 41.559 | 35e( | 4 | Yes | CHOOSE+ |
| | ***Coordinating End of Waiting Period*** | | | | |
| 32 | 78.74 | 485( | 3 | go at120? | COORD |
| 33 | 82.31 | 610: | 1 | ye | COORD |
| 34 | 86.879 | 35e( | 4 | yas | COORD |

### 3.1.21 Acknowledge Cooperation [*ENF ACK*]

A form of enforcement (social sanction): Thought units/phrases where someone either (a) announces (reports) or acknowledges their own cooperative intent/behavior or (b) another player's cooperative intent/behavior.

Example:

Player 1: let's not be betraying each other up in here [*ENF WARN*]
Player 2: you have my word [*ENF ACK*]
Player 4: right [*ENF ACK*]
we're just all out here tryna get paid [*SOC INFO*]
Player 1: pinky swear everyone? [*ENF ASK*]
Player 4: pinky promise!!!!!! [*ENF ACK*]
Player 3: pinky promise [*ENF ACK*]
Player 2: pinky promise [*ENF ACK*]
Player 1: pinky promise [*ENF ACK*]

**Note:** Higher forms of enforcement (e.g., praise, warn) trump Acknowledgement. Thus, if a statement appears to acknowledge cooperation but also includes praise, shame, etc., then use the most appropriate higher type of enforcement code.

### 3.1.22 Praise and Encouragement [*ENF PRAISE*]

A form of enforcement (positive social sanction): Thought units/phrases that: (a) complement someone for perceived good cooperation or compliance, (b) celebrate good performance or cooperation/compliance, or (b) positively/supportively encourage future cooperation, or success.

**Note:** Praise/encouragement can come before or after the actual cooperation. For example, group members may praise someone for cooperating. They may also encourage each other to follow a new agreement in the future, before the next harvest round.

Examples

- Praise (rewarding observed cooperation after a harvest round):

  "We did good!" [*ENF PRAISE*]
  "Good job Player 1!" [*ENF PRAISE*]

- Encouragement (encouraging future cooperation):

  "lets go team"

  *Said by a player just before the next round started, after the group created a conservation agreement; statement encourages everyone to cooperate.*

- Encouragement (encouraging future success):

  *Group established a private property agreement. Player 1 was struggling to "farm" his/her plot effectively, and the following encouragement occurred.*

| | | | |
|---|---|---|---|
| *49* | *-253.663* | *2* | *1 is just laughing at us now _-_* |
| *50* | *-242.727* | *1* | *im not* |
| *51* | *-241.862* | *4* | *sorry 1* |
| *52* | *-234.344* | *1* | *you gus got more than me* |
| ***53*** | ***-230.886*** | ***4*** | ***youre the hero to this story [ENF PRAISE]*** |
| *54* | *-216.773* | *1* | *when i try to grow thm they dont work for some reason* |
| *55* | *-209.635* | *2* | *we have to take the middle of the group* |
| ***56*** | ***-204.542*** | ***4*** | ***this is your plot, 1, youre the underdog this is your come up [ENF PRAISE]*** |
| *…* | | | |
| *69* | *-309.272* | *2* | ***1 you killed it [ENF PRAISE]*** |
| *70* | *-305.587* | *4* | ***that was pr solid [ENF PRAISE]*** |
| *71* | *-298.75* | *1* | *thanks* |
| *72* | *-291.612* | *4* | ***the come up [ENF PRAISE]*** |
| ***73*** | ***-269.078*** | ***2*** | ***the come back? [ENF PRAISE]*** |
| ***74*** | ***-259.433*** | ***4*** | ***its 1s come up ([ENF PRAISE]*** |
| *75* | *-254.704* | *2* | **insert thinking face emoji** |
| ***76*** | ***-241.899*** | ***4*** | ***im hardcore rooting for 1 [ENF PRAISE]*** |

### 3.1.23 Ask [*ENF ASK*]

A form of enforcement (social sanction): Thought units/phrases that solicit (ask, request) cooperation, compliance, or commitment to a group social contract (e.g., conservation agreement). The request can target one or more people.

Examples:

- "pinky swear everyone?" [*ENF REQ*]
  *Said by a player when an agreement was first created.*
- "can we please not descend into chaos" [*ENF REQ*]
  *Asking for other players to cooperate/comply with the group agreement.*

***Note:*** To count as enforcement (instead of a proposal), a social contract (e.g., conservation agreement) must already exist; if not, then the statement is likely a proposal.

### 3.1.24 Tell [*ENF TELL*]

A form of enforcement (negative social sanction): Thought units/phrases in which a person tells one or more group members to cooperate, or to do a specific act that amounts to cooperation/compliance. For these statements to count as *Tell*, they must be a directive or regulatory statement.

***Note:*** To count as enforcement (instead of a proposal), a social contract (e.g., conservation agreement) must already exist; if not, then the statement is likely a proposal.

Example 1:

*Group 16 established a conservation agreement for players to harvest slowly at the beginning of the harvest round, taking only single, outlying tokens (tokens not in a cluster), followed by a free-for-all in the last 60 seconds. The following enforcement occurred during the first Round.*

Player 3: we need to let them regenerate faster [*ENF TELL*]
*Not a goal, because they had already made an agreement.*
Player 4: lets slow down for a bit [*ENF TELL*]
Player 3: yes slow down [*ENF TELL*]
Player 4: one slow down [*ENF TELL*]
Player 3: theyre about to be gone [*ENF WARN*]
Player 3: great. [*UNCODEABLE*]
Player 2: bro 1 snatched all the tokens [*ENF SHAME*]
Player 3: exactly...... [*ENF SHAME*]
Player 1: what? No [*SOC INFO*]
Player 3: what happened to the waiting plan [*SOC INFO*] werent we going to wait until the last minute or 30 seconds or something [*RULE INFO*]

Example 2:

Player 4: #1 and #3: stay in your parts of the board [*ENF TELL*]
*Said in context of a private property conservation agreement.*

### 3.1.25 Shame [*ENF SHAME*]

A form of enforcement (negative social sanction): Thought units/phrases in which a person tries to publicly embarrass, guilt, or scold someone for perceived poor cooperation/compliance. This will have a normative (moral) component (e.g., dealing with fairness, moral “right,” stealing).

***Note:*** To count as enforcement (instead of a proposal), a social contract (e.g., conservation agreement) must already exist; if not, then the statement is likely a proposal.

Examples:

- Player 3: i think that works [*ENF PRAISE*]
  Player 1: i think 3 at {ate} them {the tokens} to quick [*ENF SHAME*]

*Said after the first harvest round, after testing a new conservation agreement.*

- Player 2: bro 1 snatched all the tokens [*ENF SHAME*]
  Player 3: exactly…. [*ENF SHAME*]

**Note:** If the presumed moral component of the statement is ambiguous (i.e., cannot be determined directly from the statement or indirectly but reliably from the context), then consider coding the statement as Acknowledgement or Tell.

### 3.1.26 Warn [*ENF WARN*]

A form of enforcement (negative social sanction): Thought units/phrases that warn (i.e., inform) someone about a hypothetical, implied, potential, or actual consequence, retaliation, or penalty for failing to cooperate, as a means of preventing future non-cooperation.

***Note:*** To count as enforcement (instead of a proposal), a social contract (e.g., conservation agreement) must already exist; if not, then the statement is likely a proposal.

Examples:

- “don’t come for my crop all i’m saying” [*ENF WARN*]
- “if this devolves into a free-for-all im gonna be pissed” [*ENF WARN*]

- Player 1: NO OFFENSE: BUT 2 you were kinda the problem most of the first rounds. the rest of us were being strategic while the first two rounds you used us and got around 70 tokens each time while the rest of us were in the 30s [*ENF SHAME*]

  Player 3: yes and if you back out of the agreement we're all coming for your corner in the next rounds [*ENF WARN*]

**Special Considerations:**

**(1) *Warn vs. Tell:*** If in doubt, code as *TELL*.

**(2) *Warn vs. Threats:*** If the “warning” includes cursing or disparaging comments (e.g., calling people names, degrading their intelligence or character; e.g., “Player 3 you f**k”), then the statement should be coded as THREAT, not *WARN*.

### 3.1.27 Threaten [*ENF THREAT*]

A form of enforcement (negative social sanction): Heated and forceful (hostile) thought units/phrases used as “weapons” and intended to punish individuals for non-cooperation, or prevent non-cooperation. Threats typically include a warning or penalty statement, but are delivered in a more hostile and extreme tone, with: curses, insults, name-calling or disparaging remarks (e.g., attacks on a person’s character). When curses are clearly in response to non-cooperation, treat them as threats.

***Note:*** To count as enforcement (instead of a proposal), a social contract (e.g., conservation agreement) must already exist; if not, then the statement is likely a proposal.

Examples:

- Player 4: #2 gtfo {get the f*** out} [*ENF THREAT*]

*Said when Player 2 entered Player 4's area (in context of private property).*

- Player 1: hey 2 go f*** yourself. Seriously
  *Said when Player 2 violated conservation agreement.*
- Player 4: f***ing stop
  *Multiple group members violating the conservation agreement.*

### 3.1.28 Coordinated Punishment [*ENF PUNISH*]

A form of enforcement (negative sanction): Thought units/phrases *plus contextual evidence* that individual(s) actually retaliated against someone, specifically by (a) using a monetary penalty (if those are enabled for the current experiment), (b) individually or (c) collectively taking tokens from the player (by harvesting tokens that are clearly theirs) with the clear (unambiguous) intent to punish or discourage non-cooperation.

***To be considered PUNISH, there must be evidence in the chat that individuals actually carried out the punishment, otherwise it is mere words and is considered a warning (WARN) or a threat (THREAT).***

- **Example of WARN:**

  Players 1, 3, and 4 warned that they would each take tokens from Player 1 if he/she continued to violate the agreement (*see Example 3 in the Warn subsection, above*). If Players 1, 3, and 4 had actually carried out this punishment (they did not), then their statements would have been coded PUNISH, instead of WARN.

- **Example of Social Sanction version of ENF PUNISH:**

  *Based on context, the bolded excerpts below are coded as PUNISH:*

  *During Round 5, Player 1 violated the group agreement:*

  Player 3: next roumd we takin everything from 1 [*ENF WARN*]
  …
  Player 1: ha goodluck [*DEFECT*]
  …

  *Before Round 6, the group had the opportunity to communicate for 5 minutes. Player 1 continued to defy the agreement and taunted the other players about it. Player 4 warned Player 1 of a potential punishment:*

  Player 4: 1 aint gon learn, might as well WILD {*go wild*} [*ENF WARN*]

  *During Round 6, Player 4 and Player 2 took as many tokens as they could from Player 1, carrying out Player 3 and 4's proposed punishment. This action resulted in Player 1 getting less tokens, and equalized the share of tokens among the players:*

| | |
|---|---|
| Player 3 | damnnnn [*MON RES: notices tokens being taken from Player 1*] |
| Player 1 | wow okay try hard [*DEFECT: retaliates with an insult*] |
| Player 3 | how did that happen lol [*SOC INFO*] |
| Player 1 | this was pretty even [*MON EARN*] |
| Player 2 | **THIS IS WHY CAPITALISM here dont work boi [*ENF PUNISH*]** |
| Player 4 | HOW ARE WE MORE EVEN [*SOC INFO, MON EARN*] |
| Player 1 | idk 4 has been ass the whole time [*DEFECT*] |

| | |
|---|---|
| Player 4 | **im S H O O K E T H [*ENF* *PUNISH*: celebrating/gloating]** |
| Player 1 | you stilll get last place but atleast you got more than 10 [*MON EARN*] |
| Player 4 | **i dont know i mean i pulled up on ya [*ENF PUNISH*]** |

### 3.1.29 Defection [*DEFECT*]

Thought units/phrases that either directly communicate or exhibit (i.e., amount to) a deliberate act of defection or defiance of a group's social contract(s). This includes taunts.

Example:

*Stated in the context of repeated agreement violations:*

Player 4: “1 you still greedy” [*ENF SHAME*]
Player 1: “lol” [*DEFECT*]
…
Player 3: “I look over and you acting wild @1” [*ENF SHAME*]
Player 1: “get rekd XD” [*DEFECT*]
….
Player 1: “L” {*repeatedly types “L” (meaning “Losers”)*} [*DEFECT*]
…
Player 1: “im going ballsdeep next round just a warning” [*DEFECT*]
Player 1: “goodluck” [*DEFECT*]

### 3.1.30 Small Talk [*SM TALK*]

Off-task statements not directly related to governance of the social-ecological dilemma. For example, sharing or discussing personal interests, popular events or topics (e.g., movies, school), and personal information (self-disclosure; e.g., personal experiences, intimacies). When coding *include both active (e.g., raising a topic) and passive comments (replies).*

***Note:*** *When coding exclude statements clearly intended as humor (though you may choose to combine SM TALK and HUMOR during analyses; cf. DeCaro et al., 2021).*

### 3.1.31 Humor [*HUMOR*]

Obvious and intentional attempts to amuse another person (e.g., jokes, silly statements, and unexpectedly odd statements). **Only use the HUMOR code if amusing other players is the *sole/primary purpose* of the statement**.

### 3.1.32 Being Polite/Impolite [*POLITE+/-*]:

### A) Polite [*POLITE+*]

Thought units/phrases in which the primary purpose (content) is to be polite or respectful. This includes (1) statements of gratitude (giving thanks) and (2) apologizing or saying sorry as (a) a gesture of kindness, (b) courtesy (e.g., for accidentally saying inaccurate information), or (c) sympathy for something unfortunate that happened to a player, outside of anyone’s control (e.g., the random distribution of tokens was less in a particular area).

Examples:

- Sharing tokens:

  Player 3: “thx 4” [*POLITE+*].
  Player 4: “got you babe” [*POLITE+*].

- Saying sorry for something outside one’s control:

  Player 1: My area started with a lot less tokens this time [*MON RES*]
  Player 2: sorry 1 [*POLITE+*]

- Restorative Justice:
  Player 4: 3 you took my tokens [*POSE HARM*]
  Player 3: sorry 4 [*RJ APOLOGY*]

**Special Considerations:**

**(1) *Enforcement trumps Politeness:***

If a thought unit/phrase includes enforcement intent, then code it as the enforcement type, not *POLITE+*. This situation is especially common with *Praise/Encouragement* (because praise/encouragement can seem polite).

**(2) *Restorative Justice (e.g., Apologies, Atonement) trump Politeness:***

If the context of a thought unit/phrase indicates that a person is apologizing (*RJ APOLOGY*), atoning for (*RJ ATONE*), or explaining/justifying (*RJ JUSTIFY*) a perceived harm or wrong (*POSE HARM*) to ameliorate the harm/wrong), code the statement as the relevant type of restorative justice: doing so better represents the statement’s primary intent. The intent is not just to be polite—it is to correct a harm to restore a sense of justice.

**B) Impolite [*POLITE-*]**

Thought units/phrases where the primary purpose (content) is to actively behave in an impolite or discourteous way, or disrespect someone. This includes intentional rude interruptions, actively refusing to give thanks, name calling, etc.

Example (Impolite):

- Player 3: “So how is everyone” [*SM TALK*].
  Player 4: “nahhhhh” [*POLITE-*]

  *Player 3 tried to start small talk, and Player 4 immediately and impolitely shuts the conversation down, dismissing it outright.*

***Note:*** Just like POLITE+, impoliteness [POLITE- ] is trumped by Enforcement, Restorative Justice, and other primary codes.

Example:

*Enforcement and Restorative Justice Trump Impoliteness*:

Player 4: 2 you made me made cuz u took all the tokens at first [*POSE HARM*]
I was trying to be equal [*SOC INFO, GOAL: EQUITY*]

Player 2: not really I was working in one quadrant [*RJ JUSTIFY: RULE INFO*]
Player 4: but no one else knew that [*ENF SHAME, SOC INFO*]
Player 2: I was like these {tokens} are mines [*RJ: JUSTIFY: SOC INFO*]
Player 4: **tf you mean? [*ENF SHAME, RJ FORGIVE-*] (*trumps POLITE-*)**
we couldn't talk how were we supposed to know [*SOC INFO*]

### 3.1.33 Discourage Input [*INPUT-*]

Thought units/phrases where someone actively tries to prevent an individual from contributing to the discussion, or encourages others to ignore particular individuals.

Examples:

- "Shut up player 1!"
- "I don't care what you have to say."

### 3.1.34 Ambiguous [*AMB*]

Thought units/phrases where the purpose/intent or meaning of the content is unclear, or that may fall into a particular category but is unclear (i.e., coding will be unreliable). *This is most likely to occur when the context for the specific statement is unclear.*

### 3.1.35 Uncodable [*UNCODE*]

Thought units/phrases for which there is no established code, or the chat text is undecipherable.

***Note:*** *If there is no existing code, then consider making a new code to capture the content.*

# CHAPTER 4: FUNCTIONAL CODING

## 4.0 INTRODUCTION

This chapter provides guidance for functional coding of group discussion/deliberation in terms of democratic decision making (i.e., shared/participatory collective choice), proportion usage of various enforcement types, and restorative justice when resolving harms/conflicts within the group. In these analyses, the units of analysis are the stand-alone/complete decision events, enforcement events, or restorative justice events. The purpose of conducting functional coding on these events is to assess group processes, which are thought to be essential to successful and robust cooperative governance of social-ecological dilemmas. These elements correspond with key dimensions (design principle) of governance articulated by Elinor Ostrom (1990, 2010).

## 4.1 DEMOCRATIC DECISION MAKING

Open communication and collective (i.e., shared/participatory) decision making are central components of cooperative governance in social-ecological dilemmas (Ostrom 1990, 2010). Communication and decision making within a group or collective governance context can be classified as relatively fair and autonomy-supportive (facilitative), or unfair and controlling (coercive). This fairness can influence individual motivation and group cooperation (e.g., DeCaro et al. 2015, 2021). Our concept of decision fairness and autonomy-support is based on foundational concepts in procedural justice research, self-determination/autonomy-support, and cooperative governance and democracy (e.g., Colquitt 2001, Tyler 2006, Deci and Ryan 2000, 2017; Ostrom, 1990, 1994; see DeCaro et al. 2015, 2021; DeCaro et al. 2020 for synthesis).

Vincent Ostrom (1980, 1994) distinguishes between two fundamental types of power. When group members attempt to forcefully control or dominate others—even for mutual or paternalistic/benevolent benefit—they are exerting ***power over*** others. Generally, this will have the appearance of unilateral decision making by an individual, leader, or faction (i.e., minimal winning coalition), thereby restricting some members from participating fully in deliberation and institutional design (voice), institutional selection (choice), or both. In contrast, when group members endeavor to share power, making decisions inclusively, they are exercising ***power with*** each other. This type of power generally involves shared decision making and obedience to democratic processes, rather than to a leader or center of authority (DeCaro 2011).

This basic concept of power has been meaningfully developed in other theoretical traditions, including controlling versus autonomy-supportive interpersonal and organizational environments (Ryan and Deci 2017), procedural justice in law and organizations (Tyler 1990, 2006), procedural utility (Frey et al. 2004), and directive versus facilitative organizational leadership (e.g., De Cremer and Tyler 2005). Our coding system is inspired by these theoretical traditions but is further informed by social cognition, behavioral economics, and theory of collective action, especially Humanistic Rational Choice Theory (DeCaro, 2018, 2019). The system is also grounded in the study of social dilemmas (see DeCaro et al. 2015, 2021 for review).

We describe 3 potential indicators of fairness/autonomy-support based on prior research and theory: the extent to which (1) groups make decisions democratically, using inclusive group decision strategies, (2) individuals communicate equally, and (2) individuals communicate equally specifically during decisions. Researchers do not need to calculate all three indicators. By default we recommend calculating the democratic decision-making index, which exhibited

the strongest association with psychosocial processes and cooperative outcomes in DeCaro et al.'s (2021) first test of the coding system in a communication experiment.

### 4.1.1 Democratic Decision-Making Index

The *democratic decision-making index* (DDMI) quantifies the extent to which group decisions and their resultant social contracts (e.g., conservation agreements) were, on average, decided together, with each group member contributing to the overall preference (i.e., collective choice). As described in DeCaro et al. (2021), this index is based on the principle of revealed preferences in behavioral decision making and is inspired by similar indices in prior experiments (Vyrastekova and van Soest, 2003; Yu et al., 2016 ):

**To Calculate the DDMI:**

Identify each major decision event (see *Section 2.2.3*: *Identify Decision Events*). Then, examine each decision event to determine (count) how many group members chose (i.e., *CHOOSE+*) the focal proposal (e.g., conservation strategy). With 4 group members, this score could range from 0 (*no members; or unresolved /contested*) to 4 (*4-person majority*). Average the scores across the group's total number of decision events. The result is an overall indicator that represents the extent that group decisions were decided by all four group members.

**To Classify Each Decision Event:**

When classifying each decision event, use the following operational definitions and scale (e.g., an unresolved/contested decision = "0"). **The *Choose+* code is used to identify positive affirmation (i.e., choosing) in conjunction with the *PROPOSE* code (the person who proposes the proposal is assumed to also *choose* that proposal); therefore, these codes are the basis for counting support for proposals.** *We recommend maintaining a list of each decision event at the end of each group's coding form (e.g., worksheet), with the line numbers and a brief description for easy reference; record the score for each decision event there.*

**(0) *Unresolved/Contested Decision.*** Either the decision is not resolved (no conclusion reached) or is contested (i.e., 2 people *CHOOSE*+ and 2 people *CHOOSE-*).

**(1) *Unilateral Decision.*** A decision is made by a single individual (i.e., proposed and imposed by the same single person), without apparent choice (*CHOOSE*+) by others.

**(2) *2-Person Majority.*** Two individuals support (*CHOOSE*+) the proposal, with the remaining individuals being split (supporting different proposals), undecided, or non-vocal (i.e., not stating their preference).[7]

**(3) *3-Person Majority.*** Three individuals support (*CHOOSE*+) the proposal.

**(4) Consensus (4-*Person Majority*).** All four group members support (*CHOOSE*+) a proposal. This decision strategy will sometimes be synonymous with a unanimous majority vote, where all four players explicitly approve a proposal by vote (4/4 choosing). In other cases, consensus may be simple voicing of support or indicated/implied by an individuals' implicit adoption of the proposed choice option (e.g., conservation strategy).

---

[7] ***Minimal Winning Coalitions:*** It is possible for 2-3 members to form a coalition that consistently dominates the other member(s). Such a situation would not be considered a fully participatory process, unless explicitly endorsed by the minority member(s). *Note minimal winning coalitions, and consider analyzing as a moderating factor.*

**To Calculate the DDMI Index:**

To calculate the DDMI, we take the average decision-making score across all of a group's decision events. If we assume that individuals exercise and express their willful (autonomous) preference by voicing support for proposals, then the following index serves as a proxy for individual/group self-determination (cf. Vollan 2008:568).

Examples from DeCaro et al. (2021):

| | Group 1 | Group 2 | Group 41 |
|---|---|---|---|
| Decision 1 | 4 (*consensus*) | 4 (*consensus*) | 4 (*consensus*) |
| Decision 2 | 2 (*2-person majority*) | 4 (*consensus*) | 0 (*contested/unresolved*) |
| Decision 3 | 3 (*3-person majority*) | 4 (*consensus*) | |
| Decision 4 | 1 (*unilateral*) | | |
| Decision 5 | 2 (*2-person majority*) | | |
| *DDMI:* | 12/5 = 2.40 | 12/3 = 4.00 | 4/2 = 2.00 |

### 4.1.2 Communication Equality

Here, we focus on overall communication, in order to calculate an overall indicator of communication fairness, or equality. To do this, we use a method established by Janssen (2010; see also, Rogat and Adam-Wiggins 2014:833).

***Gini Coefficient of Communication*** **(Gini Comm):**

The Gini Coefficient is a method of quantifying the amount of inequality in a population. The Gini Coefficient is often used to calculate economic inequality (Lerman and Yitzhaki 1984). We use it to quantify the extent to which communication opportunity is shared equally among group members. The Gini coefficient ranges from 0 to 1: Gini = 0.00 represents perfect equality (all four group members communicate equally), whereas Gini = 1.00 represents perfect inequality (a single person is the only one communicating). It has been proposed that this coefficient may be a general indicator of fairness and autonomy-support in group interaction (DeCaro 2019), and/or group liking and cohesion (Janssen 2010).

**Calculation:**

A. Count the total number of thought units[8] contributed by each individual, across a group's entire communication (i.e., all of a group's communication/thought units).
B. Compute the Gini Coefficient. For simplicity, we entered the count values into the open-source calculator provided by Conrad (2007).

### 4.1.3 Deliberative Equality

The purpose of this section is to calculate the extent that individual group members contribute equally to deliberation and discussion of important decisions, as an indicator of fairness and autonomy-support during decision making (i.e., extent that individuals have or exercise equal opportunity to voice their opinions during decisions). Hence, this calculation isolates decision-specific communication from other (i.e., overall) communication.

[8] Note that Janssen (2010) computed this index on *statements* (i.e., chat lines), not thought units.

***Gini Coefficients*:**

We calculate two versions: a standard version and an adjusted version.

The **(standard) Gini Coefficient of Deliberation** is computed based on the number of thought units contributed by each individual during each decision event. The assumption is that equal participation, specifically in terms of in contributing one's thoughts or opinions during deliberation, corresponds with equal voice and, therefore, fairness.

The **Adjusted Gini Coefficient of Deliberation** is computed by taking into account the practical and theoretical consideration that some individuals may not wish to communicate much during deliberation of ideas, yet may still perceive the decision process as fair. This situation is especially commonplace when an individual participates in the final decision by voting or indicating their choice among final options, but does not contribute ideas or opinions for the design and deliberation of those options. Research distinguishing between such "voice" and "choice" aspects of fairness and notes that some individuals prefer not to contribute to deliberation, but instead prefer to exercise choice and, therefore, still feel a sense of procedural fairness and autonomy from contributing to the final decision, especially if they endorse the group's leadership and/or overall decision-making process (de Cremer and Tyler 2005; DeCaro et al. 2015). Thus, an individual may defer deliberation to their fellow group members, and exercise choice when it comes time to choose among the options that have been created. A simple (standard) Gini coefficient may assign too much weight to inequalities produced by individuals who willingly contribute few ideas during deliberation for this specific reason, overestimating perceived inequality (cf. DeCaro & Stokes, 2013). We therefore calculate an adjusted coefficient that corrects for this potential bias (see calculations below).

**Calculations:**

(1) **Standard Coefficient.** Calculate a Gini Coefficient for *each decision event*. Use the number of thought units contributed by each individual for each event.

> For example, Group 1 had a total of 5 decision events. In Decision 1, Player 1 contributed 1 thought unit, Player 2 contributed 3, Player 3 contributed 3, and Player 4 contributed 6, resulting in a Gini = 0.2885 for Decision 1.

(2) **Adjusted Coefficient**. If an individual (a) contributes few thought units to the group's decisions (nominally 1-5 thoughts, proportionally approximately less than 10% of the statements), OR (b) none of those statements are *proposals* that contribute to the substance of deliberation or design of a solution/agreement, AND (c) the individual explicitly states their agreement or endorsement of the group's decision, then **this individual is dropped from ALL the counts in Step A, and ALL Gini Coefficients are re-calculated as if that individual does not exist**. This procedure assesses the degree of equality among the remaining (i.e., leading) group members who are engaged in the deliberation process of the decision.[9] *Report all such corrections in the data file and published results.*

[9] If you wish to test the robustness of this correction, then calculate the Gini Coefficient with and without the adjustment, and note the effects doing so has on important perceptions and outcomes.

For example, Group 1 had 5 decision events. We omitted Player 1 because across all 5 decisions, Player 1 contributed only 1 thought unit (in Decision 1), and this thought unit was to express agreement with the group's decision to use a private property rule as their primary conservation strategy. The corrected Gini DM = 0.1667.

(3) **Take the average** of the Gini coefficients calculated for all of a group's decision events to get the overall score for the group.

For example:

Group 1's Gini DM coefficients were:

| Original | Adjusted |
|---|---|
| Gini DM 1 = 0.2885 | Gini DM 1 = 0.1667 |
| Gini DM 2 = 0.5000 | Gini DM 2 = 0.3333 |
| Gini DM 3 = 0.4167 | Gini DM 3 = 0.2222 |
| Gini DM 4 = 0.3214 | Gini DM 4 = 0.0952 |
| Gini DM 5 = 0.3125 | Gini DM 5 = 0.0833 |
| **Average** = 0.3678 | **Average** = 0.1801 |

## 4.2 ENFORCEMENT INDEX

Groups need ways to compel group members to adhere to their agreements. Enforcement is a centrally important activity of governance in a social-ecological dilemma (Ostrom 1990). The effects of various types of enforcement on cooperation, whether positive reinforcement (e.g., praise, encouragement) or negative punishment (e.g., shame, penalties), are debated (see Bowles 2008; DeCaro et al. 2015, 2021 for review). The purpose of this coding step is to identify the enforcement methods used by group members to compel each other to comply with the group's rules, strategies, or agreements, in order to facilitate their empirical analysis.

When groups communicate but are unable to use tangible, economic sanctions or other incentive- or economic-based penalties to prevent defection or punish uncooperative behavior, then individuals will primarily rely on social sanctions (cf. Ostrom 1990, Janssen et al. 2010). We distinguish between ***positive social sanctions*** (e.g., praise), which encourage and reward cooperation, and ***negative social sanctions*** (e.g., shaming, warnings, threats), which seek to discourage defection through verbal penalties, shame, and other directive or negative forms of reinforcement. In some cases, group members may devise ways to coordinate their harvests, specifically to target and reduce the earnings of another group member, or multiple group members, creating a more tangible economic penalty for defection. For simplicity, we include this latter form of tangible, negative sanction with negative social sanctions.[10]

As described in Sections 3.1.20 to 3.1.27, we identify 7 social sanctions (types of enforcement):

1. ***Acknowledge*** self or other's cooperation. (neutral)
2. ***Praise, Celebrate, or Encourage*** cooperation. (positive)
3. ***Request*** cooperation. (neutral/positive)
4. ***Tell*** someone to cooperate. (negative)
5. ***Shame*** someone for not cooperating. (negative)
6. ***Warn*** someone to cooperate. (negative)
7. ***Threaten*** someone to cooperate. (negative)
8. ***Punish*** someone for not cooperating. (negative)

**Special Considerations:**

***Distinguishing Enforcement from Goals and Proposals:***

Enforcement statements (technically, thought units/phrases) **can sometimes be confused with goals and proposals** (e.g., proposed solutions). In order for a statement to count as enforcement, there must first be either (a) a shared agreement (i.e., a previously chosen proposal, resource management strategy) to enforce or (b) a reasonable expectation (common understanding/norm) that individuals ought to behave in a particular way, with respect to the resource or each other. If neither prerequisite has been met, then the statement is most likely either a goal or a proposal, not enforcement.

[10] Another reason for including coordinated punishment with verbal/social negative sanctions is because this type of sanctioning appears to be rare in communication-only lab experiments. The rarity of coordinated punishment is likely due to the fact that this type of enforcement is more complex than, for example, simply verbally shaming a defector, because it requires coordination among multiple individuals. In addition, individuals (in our samples) arguably have more experience using social sanctions on others, not economic sanctions (which is typically left to governments and actors in position of authority, e.g., law enforcement).

**Thus, most thought units that look like enforcement *when group members first start to communicate* are most likely goals or proposals.** And, these goals and proposals must be converted to agreements before they can be "enforced."

***The main exception to this guideline pertains to Shaming and Threats:*** individuals may logically try to shame or threaten others to share tokens more equally before a specific solution has been proposed, because equal sharing of tokens (i.e., equity) is a common norm.

**Scoring:**

With frequency coding (see Chapter 2), researchers can calculate the correlation of the root frequency of particular types of enforcement with psychosocial processes and cooperative outcomes. When scoring sanctions as a functional category, the objective is to calculate the extent that a group relies on particular methods of enforcement overall, specifically the relative use of positive versus negative social sanctions. Generally speaking, groups that rely more on negative sanctions (e.g., shame, threats) can be considered to function differently than groups that rely more on positive sanctions (e.g., praise, encouragement) (cf. DeCaro et al., 2021).

Thus, the following steps guide you to (a) calculate **enforcement scores as percentage usage** out of the total number of enforcement instances (i.e., enforcement events) within each group, and (b) then **subtract the proportion of negative sanctions from the positive sanctions** to derive a difference score (i.e., relative usage index).

**(1) Identify the Enforcement Events**. An enforcement event occurs whenever an individual uses one of the listed enforcement methods: a single thought unit/phrase counts as an enforcement event. *We recommend you list all the enforcement events at the end of a group's coding form (e.g., worksheet), and record the line number associated with each such event for easy reference.*

**(2) Calculate Percentage Use:** Count the number of times (thought units/phrases) that a particular method is used, and then calculate the percentage each is used out of the total number of enforcement events (i.e., out of 100%).

Example: Group 3 (DeCaro et al. 2021)

| Code | Count (%) |
|---|---|
| Acknowledge | 0 (0%) |
| Praise/Encourage | 17(70.83%) |
| Request | 0(0%) |
| Tell | 2(8.33%) |
| Shame | 3(12.50%) |
| Warn | 2(8.33%) |
| Threaten | 0(0%) |
| Punish | 0(0%) |

**(3) Calculate Difference Score:** There are many possible ways to calculate potential difference scores, or other metrics, after you know the proportion usage of each enforcement type. By default, we recommend that you calculate the difference score using/comparing the most obvious (unambiguous) positive versus negative types of sanctions: i.e., praise minus the total proportion of warn, threat, and punish combined. For example (Group 3): 70.83(%) positive – 20.83% (negative) = 50.00.

## 4.3 RESTORATIVE JUSTICE INDEX

Conflict resolution and the responsiveness of cooperative systems to perceived harms/injustices (e.g., inequities in costs/benefits, unfair rule-enforcement) is posited to be essential to the long-term legitimization, acceptance, effectiveness, and robustness of cooperative governance systems (Ostrom 1990; cf. Johnstone and Van Ness 2013; Tyler 2006a-b). Many real-world cooperative governance systems use some form of enforcement to ensure robust forms of cooperation and compliance (Ostrom 1990, Cox et al. 2010). However, enforcement systems may backfire if used inappropriately (Bowles 2008). Ostrom (1998, 2000) argued that effective enforcement systems do more than deter—they serve vital community-building functions, which reduce conflict and promote voluntary cooperation. Many successful cooperative governance systems use graduated sanctions (that increase in severity with each violation) and deliberative democracy (e.g., hearings, open dialogue) to simultaneously enforce rules, justify enforcement, educate, and rehabilitate defectors (Ostrom, 1990). This restorative justice perspective recognizes that there are systemic reasons for defection that may justify non-compliance: e.g., unclear rules, perceived inequity in costs/benefits of compliance, unfair decision procedures, flawed rules and enforcement systems (Johnstone and Van Ness 2013; Tyler 2006a). Effective enforcement systems are responsible for educating individuals about appropriate rules and norms, upholding procedural justice, and binding communities closer together, making enforcers, violators, and victims accountable to one another (cf. Barnes et al., 2013).

In this section, we provide guidelines to assess some important aspects of restorative justice in social-ecological dilemma experiments with communication to examine these broader community-building and rehabilitative aspects of enforcement that seem to underlie effective conflict resolution and sanctioning systems.

### The Restorative Justice Index (RJI):

The unit of analysis for the *restorative justice index* (RJI) is the stand-alone/complete restorative justice event. Thus, first identify each major restorative justice event (see *Section 2.2.4*: *Identify Restorative Events*). Then, examine each restorative justice event to determine (count) how many such events were resolved amicably/productively by the individuals involved. After all the events have been accounted for, you will calculate the percentage of amicably resolved events, as an overall (average) score, which represents the extent to which groups tend to engage in restorative forms of conflict resolution and management of their enforcement systems.

### *Scoring Restorative Justice Events:*

*Restorative justice events* occur when individuals and/or groups attempt (or fail to attempt) to resolve perceived or potential (e.g., anticipated) harms/injustices (*POSE HARM*) cause by (a) rule violations (e.g., when a player harms others by violating a conservation agreement), (b) enforcement events[11] (i.e., when social or economic sanctions are used: *ENF* codes), or (c) inequities/unfairness in the group's institutional arrangements, cost/benefits of those arrangements, resource allocations, and/or procedural implementation.

When scoring individual events, look to see how the proposed harm was resolved. By default, the restorative justice codes *RJ APOLOGY, ATONE, JUSTIFY, RJ-, and FORGIVE +/-* can be used to make this judgment, though groups may potentially use other methods to resolve the event (e.g., initiate a constitutional decision to resolve a root, institutional cause of the harm).

[11] See Sections 3.1.20 to 3.1.27 and 4.2 for discussion of identifying and segmenting enforcement events.

**Restorative justice events will be considered amicably/productively resolved when:**

a) The player(s) who are deemed responsible for the perceived harm/injustice (*POSE HARM*) make amends by apologizing (*RJ APOLOGY*) or atoning (*RJ ATONE*). *Note: forgiveness is not required here, BUT FORGIVE- does cancel amicable resolution.*

b) Or: The responsible player(s) provide a rationale (*RJ JUSTIFY*) for their apparently harmful behavior *<u>and</u>* the justification is accepted (*FORGIVE+*) by the harmed player(s) (i.e., the harmed player(s) forgive the player(s) that harmed them).

c) Or: Some other action to resolve the harm/injustice is undertaken *and* the harm is forgiven (*FORGIVE+*) by the harmed player(s).

d) Or: A Constitutional Decision is made, which subsequently resolves a root institutional cause of the harm/injustice (e.g., conservation agreement revised to make costs/benefits more equitable; enforcement system revised to address an injustice). In order for this solution to count as amicable/productive, the Constitutional Decision process will have to result in a successful resolution, accepted by the key players.

***Calculating the RJI:***

After all the restorative justice events have been scored, you will count the number of events that were resolved amicably and divide by the total number of events: this calculation will give you the percentage of amicably resolved events, or the restorative justice index (RJI).

Example 1:

For example, the following group had two restorative justice events. Both were triggered by individuals taking tokens from other players, prior to communication. Both events were resolved amicably, resulting in RJI of 100%, indicating that the group tended to resolve conflicts using restorative techniques.

Restorative Justice Event 1:

*This event starts with Player 2 upset at the other players for collapsing the resource and taking tokens Player 2 felt should have been Player 2's.*

Player 2: I have some non physical threat choice words for you [*POSE HARM, SHAME*]<br>
Player 4: #2 you can have 4 of my tokens. fairs fair [*RJ ATONE*]

*Score:* 1 (*resolved amicably*)

Restorative Justice Event 2:

| | |
|---|---|
| Player 3: | also y'all took my tokens i had [*POSE HARM*] |
| Player 2: | yea you took 3's token [*POSE HARM*] |
| Player 2: | 3 gets 4 of your tokens [*PROPOSAL, RJ ATONE*] |
| | i dont need it [*SOC INFO*] |
| Player 3: | thx 2 <3 [*FORGIVE+*] |
| Player 4: | oh fine 3 you get them [*RJ ATONE*] |
| Player 3: | thx [*FORGIVE+*] |

*Score:* 1 (*resolved amicably*)

*RJI:* 2/2 = 100%

Example 3:

The following group engages in a series of restorative justice events triggered by various players violating their conservation agreement (slow-harvest agreement). The first violation by Player 3 is immediately resolved amicably, but the remaining violations/harms are not resolved amicably, resulting in continued and greater (i.e., escalating) disputes. Overall, the group has a RJI of 50% which indicates that the group is inconsistent when resolving conflicts (resolving half amicably and not resolving the other half amicably).

Restorative Justice Event 1:

Player 3: my bad [*RJ APOLOGY*]

*Score:* 1 (*resolved amicably*)

Restorative Justice Event 2:

| | |
|---|---|
| Player 4 | CHILLLL [*ENF TELL*] |
| | YALL EATING THM ALL DAMN [*POSE HARM, ENF SHAME*] |
| Player 2 | CHILL [*ENF TELL*] |
| | wATI {wait} [*ENF TELL*] |
| Player 4 | 2 WTFFF [*ENF THREAT*] |
| Player 2 | CHIILLLL [*ENF TELL*] |
| Player 4 | YALL ALL GOT 2x what i got [*POSE HARM, ENF SHAME*] |
| Player 3 | wait till 20 [*ENF TELL*] |
| Player 4 | bastards [*ENF THREAT*] |
| Player 2 | peasant [*POLITE-*] |
| | coem get this 44 [*AMBIGUOUS*] |
| Player 4 | selfish [*ENF SHAME*] |
| Player 3 | see if we can get 1 more [*PROPOSE, RJ ATONE*] |
| Player 2 | 4 come get this\ [*AMBIGUOUS*] |

*Score:* 0 (*unresolved/not amicable*)

Restorative Justice Event 3:

*In this event, Player accidentally penalized (economic sanction) Player 3.*

| | |
|---|---|
| Player 3 | why did you penelize me so much [*POSE HARM, SOC INFO*] |
| Player 4 | i meant to do it on 2 [*RJ JUSTIFY, SOC INFO*] |
| | Sry {sorry} [*RJ APOLOGY*] |

*Score:* 1 (*resolved amicable*)

Restorative Justice Event 4:

*This event begins when a newly created private property agreement is violated by Player 4.*

| | |
|---|---|
| Player 2 | #4 fuck you man [POSE HARM, *ENF THREAT*] |
| | what the shit bro [*ENF THREAT*] |
| Player 4 | life is meaningless [*SOC INFO*] |
| Player 2 | very cool kanye [*ENF SHAME*] |
| | now fuck off [*ENF THREAT*] |
| Player 3 | that hurted [*POSE HARM*] |
| Player 4 | that sit hurted huh [*POSE HARM*] |

| | |
|---|---|
| Player 3 | yes [*POSE HARM*] |
| Player 2 | yea you dick head [*ENF THREAT*] |
| Player 4 | i wanted to work as a team [*SOC INFO*] |
| | but yall too greedy [*RJ JUSTIFY: SOC INFO, ENF SHAME*] |
| Player 4 | now its just free for all [*MON BEH*] |
| Player 2 | you prolly the kid that would throw away his pizzza and not give to a kid [*ENF SHAME*] |
| Player 3 | being more greedy is counter prouctive [*SOC INFO*] |
| Player 4 | not for me [*RJ-*, *DEFECT*] |
| Player 3 | if you don't care about the money stop playing [*TELL*] |
| Player 2 | you suck man EXACTLY [*ENF SHAME*] |
| Player 4 | i need to pay rent [RJ JUSTIFY: *SOC INFO*] |
| Player 2 | JUST STI IN A CORNER TO DIE [*FORGIVE-*, *ENF THREAT*] |

*Score:* 0 (*unresolved/not amicable*)

*RJI:* 2/4 = 50%

# CHAPTER 5: CODING INSTITUTIONAL ARRANGEMENTS

## 5.0 INTRODUCTION

This chapter provides guidance for identifying the institutional arrangements groups create to manage the shared resource pool (i.e., conservation agreements/strategies) and enforce social contracts (i.e., enforcement systems). Here, the term “enforcement system” has a broader meaning to include the *rules and norms surrounding* the use of, for example, monetary penalties. This means we identify when, why, and how specific forms of enforcement (e.g., praise, monetary penalties) are used by the group, as specified by their social contracts. The purpose of this coding is to enable researchers to achieve three potential research goals:

a) Examine potential effects of specific institutional arrangements on group performance (e.g., cooperation, resource sustainability, earnings)

b) Examine evolution of the institutional arrangements across constitutional decision events and/or rounds of the experiment.

c) Assess the quality of those institutional arrangements, as well as group members’ corresponding conceptualization and understanding of the social and ecological dynamics those institutional arrangements are intended to address.

## 5.1 CONSERVATION STRATEGIES

Researchers commonly identify the conservation strategies that groups develop to manage the shared resource pool (e.g., Janssen, 2010; Janssen et al., 2010; Yu et al., 2016). We adopt similar methods (see DeCaro et al., 2021).

To identify the conservation strategies a group uses, you can code their in-game communication, examining the constitutional decision events. If you use surveys to gather information from participants, you can also ask them about the conservation strategies their group created and used. We used both sets of information to code the group strategies in DeCaro et al. (2021); such an approach allows you to triangulate information to derive a more accurate account of the institutional arrangements, but is optional.

Here, we describe how to code in-game communication to identify the conservation strategies (see DeCaro et al., 2021 for details about triangulating with survey information). To identify conservation strategies from the group communication, we recorded any conservation strategy mentioned and supported/approved by at least two group members (see Yu et al., 2016 for a similar methodology). Groups will typically use a combination of strategies, creating a more complex strategy consisting of multiple elements. For example, in DeCaro et al. (2021), most groups (78%) used some form of private property, often (54%) combined with slower harvest rate, waiting for a particular period of time before starting to harvest, and/or harvesting in a particular pattern to optimize regrowth of the resource (e.g., checkerboard pattern).

Group members could potentially create any number of potential strategies, with unique variations (e.g., different private property arrangements) and combinations. However, the most common strategies (and/or elements of complex strategies) are:

- ***Private Property*****.** Groups divide the playing field into even sections, with each individual have completely independent or quasi-independent control of “their” section. Typically, groups divide the field into four, equally-sized corners.
- ***Delayed Harvest.*** Group members wait a particular length of time before harvesting any tokens. Common delays are 30, 60, or 120 seconds, but any is possible.
- ***Cultivate Clusters.*** Group members attempt to encourage growth of clustered tokens by not harvesting from clustered tokens until near the end of the round. They do harvest lone tokens (single tokens) that are not associated with a cluster.
- ***Thin Clusters.*** Group members attempt to cultivate clusters while selectively harvesting a small number (1 to 2) of tokens from the center of the cluster during the round. At the end of the round they harvest everything, including the clusters. This strategy is an approximation of the superior, checkerboard harvest pattern.
- ***Checkerboard Pattern.*** Group members attempt to encourage maximal regrowth during the round by harvesting tokens in a checkerboard pattern (harvesting tokens such that there is one open space between every-other token): x o x o x<br>o x o x o
- ***Slower Harvest.*** Group members slow down the rate of harvest (i.e., they do not harvest as fast as possible).
- ***Cyclic Harvest.*** Group members harvest in a cyclic fashion (e.g., harvest 30 seconds, pause 30 seconds).
- ***End-of-Round Harvest***. Group members wait until a specific amount of time is left at the end of the round to harvest (e.g., wait until the *last* 30, 45, or 60 seconds to harvest).
- ***Quota/Equality.*** Group members divide the available tokens up equally among the players, ensuring that each person collects the same number of tokens each round (e.g., everyone collects 75 tokens).
- ***Free-for-All at End-of-Round.*** Group members harvest anywhere they want and as quickly as they want *at the end of the round*.

Here is an **example of a complex strategy** consisting of multiple elements, observed in DeCaro et al.’s (2021) prior experiment: A group uses private property with a 45-second delay in initial harvest, combined with a checkerboard harvest pattern, and free-for-all during the last 60 seconds of the round. This strategy is close to the optimal strategy (see *Section 1.3 Social Optimum* reported in DeCaro et al.’s 2021 online supplement file/appendix).

**Scoring**

We recommend three general approaches to score the conservation strategies for qualitative or quantitative analyses. These approaches are complementary; they can all be conducted in a single study, depending on the scope and goals of the project.

1) You may wish to record the **frequency** of particular conservation strategies or elements across all groups (i.e., how many groups used particular strategies/elements). In DeCaro et al. (2021), we recorded the *final* conservation strategy/elements that each group ultimately decided to use; we then counted the frequency of particular strategies/elements across all the groups. This information was helpful for qualitative, **descriptive information**.

2) You may wish to **assign a coding category (label)** to each major type of conservation strategy to facilitate quantitative analyses, **correlating the type of strategy with observed outcomes** such as resource sustainable/earnings (cf. Janssen, 2010; Janssen et al., 2010; Yu et al., 2016). In DeCaro et al. (2021), we identified each major strategy and correlated this with group cooperation/resource sustainability. This approach allows researchers to determine whether there is any potential dis/advantage to a particular type of conservation strategy that may affect ease of coordination and/or actual resource sustainability or earning potential of the group. For example, in DeCaro et al. (2021), we conducted two analyses: (a) examining cooperation as function of private property strategies vs. all other strategies and (b) as a function of simple private property, vs. private property combined with other elements, vs. all other strategies.

3) **Quality.** The various conservation strategies differ in terms of their effectiveness at encouraging resource regrowth, and sustainability. Generally speaking, any strategy is better than no strategy (Ostrom, 1990; cf. Janssen, 2010). However, some strategies are more optimal than others. We suggest the following scoring system to approximately score or evaluate the relative (ordinal) difference in optimality. These scores can then be used in a quantitative analysis, correlating the quality of the strategy with outcomes.

   ***To account for complex, multi-component strategies, we treat the scoring categories as "nested," such that each subsequent category builds upon the prior category, in some cases adding additional components. You should select the simplest (lowest) score that most closely describes the observed strategy.***

   **(0) None.** Group has no strategy (free-for-all).

   **(1) Slow <u>or</u> Private Property.** The group either uses (a) at least one method to slow the rate of harvest (i.e., slow down, delay harvest, end-of-round harvest, cyclic harvesting) OR (b) private property (not both "a" and "b"). *Note: end-of-round harvest may be a free-for-all or a faster final harvest without free-for-all.*

   **(2) Slow <u>and</u> Private Property.** The group uses a combination of (a) at least one method to slow the rate of harvest <u>and</u> private property.

   **(3) +Sustainable Management of Clusters.** In addition (or instead of private property), the group cultivates/grows clusters until end of round.

   **(4) +Checkerboard.** In addition (or instead of private property), the group harvests the tokens in a checkerboard pattern, quasi-checkerboard pattern, thins clusters by selectively harvesting tokens from the middle of clusters.

   ***Rationale***

   Groups typically devise a range of strategies. Group researchers often have small sample sizes (too few to allow every unique strategy to be separately coded and correlated with outcomes). This quality scoring system strikes a balance, accounting for diversity with a relatively small set of five categories. The categories increase in institutional complexity, and ecological sophistication. For example, a group that combines slow harvest with a checkboard pattern—with or without private property—(score: 4) creates a more complex institution and demonstrates more sophisticated understanding of the ecological dynamics that determine resource growth and sustainability (accurately addressing more ecological elements). Thus, the scoring system provides an approximation of the complexity seen in institutional arrangements as well as insight into the group's ecological understanding.

**Additional Considerations**

Our goal is to provide researchers with a basic set of options, which they can modify as needed. Thus, researchers may find that their data is better captured by a different ordering of quality scores (or by combining some of the five scoring categories to create a smaller, consolidated set). In addition, researchers may wish to report or evaluate change or evolution in the conservation strategies over different timescales.

*Institutional Evolution*

Researchers have many options in choosing the timescale with which to record and evaluate the conservation strategies. In our typical experiment (e.g., DeCaro et al., 2021), consisting of multiple communication rounds, researchers could record (a) the strategy a particular group used the most across all the rounds, (b) change in the strategy within each round (i.e., from one constitutional decision event to the next), and/or (c) change in the strategy after each round. The latter option would allow researchers to correlate the strategy used each round with group outcomes (e.g., cooperation, resource sustainability) each round and over time, across rounds. This may be helpful if researchers are, for example, determining whether or not and how soon groups in particular experimental treatments reach (or approximately reach) an optimal conservation strategy (cf. Yu et al., 2016).

## 5.2 ENFORCEMENT SYSTEMS

Here, we describe how to code in-game communication to identify the enforcement systems that group members developed. We also describe methods to evaluate the group's conceptualization or understanding of enforcement.

Group members could potentially create any number of potential *enforcement systems* (i.e., institutional arrangements and norms governing the type and usage of social and/or economic sanctions). However, we have identified the most common forms observed in current and prior research, using the foraging task (e.g., DeCaro et al., 2021; Janssen et al., 2010).

**Coding the Enforcement System (ES)**

To code enforcement systems, you will examine constitutional decision events that pertain to use of monetary penalties (i.e., Constitutional Decision: Enforcement), and any other discussion that is *relevant to the rules/norms that determine how group members will us*e economic sanctions. Assign one of the following scores, which range from having no formal system to govern economic sanctions, to having coordinated punishment, the most complex system. An enforcement system is coded if at least two group members agree to the system.

(0) **N/A** (none created; no formal enforcement system/agreement).

(1) **Do Not Use.** The group *actively* chooses not to use monetary penalties (by vote or voiced consensus). In essence, this arrangement constitutes an agreement to voluntarily cooperate or a "treaty" against using economic sanctions.

(2) **Independent Sanctions.** Individuals may independently use penalties to punish/deter someone who violates the group's conservation or enforcement agreement(s).

(3) **Coordinated Punishment.** Group members decide to coordinate their penalties to punish/deter someone who violates the group's agreement(s). For example, two or more players agree to both penalize anyone who breaks an agreement.

***Clarification of "0: no enforcement system":***

This score is appropriate when one of the following criteria is true:

- The group has no constitutional decision events about enforcement systems.
- The group members have constitutional decision events about enforcement but fail to reach an agreement. For example, the group is split 50/50 with half the group members supporting one enforcement system and the other half supporting a different system.

  *Note: In either situation, the group may use informal social sanctioning.*

***Examples:***

1) **N/A (No System).** A group has 2 constitutional decision events, and none of those events are about enforcement systems, OR two group members support one system the other two support another and this is not reconciled.

2) **Do Not Use.** A group holds a constitutional decision event, in which a simple majority (of at least 2 members) agrees to no use monetary penalties/economic sanctions.

   Player 1: no body use penalties [*PROPOSAL*]
   Player 3: I agree [*CHOOSE*+]
   Player 4: me too [*CHOOSE*+]
   Player 2: same [*CHOOSE*+]

3) **Independent Sanctions.** Group members agree that players can individually / independently penalize other players for breaking an agreement. But the group does *not* go the additional step of agreeing to work together to jointly penalize the rule violator.

   Player 2: only use monetary penalties if someone is collecting too soon from the big chunks of tokens [*PROPOSAL*]
   Player 4: I think its fine using them if someone is collecting way to fast [*CHOOSE*+]
   Player 2: I don't really like them unless [*SOC INFO*] we are trying to keep each other from causing the tokens not to regenerate [*RULE INFO*]
   Player 1: Maybe give one if they get in your corner [*PROPOSAL*] (CHOOSE+)

4) **Coordinated Punishment.** Group members agree to jointly penalize anyone who breaks an agreement.

   Player 4: I don't think we should use monetary fines [*PROPOSAL*] unless it is very obvious that someone is just getting every dot they can [*SOC INFO*]
   Player 1: Im okay with penalties as punishment for damaging moves [*CHOOSE*+]
   Player 2: im cool with that [*CHOOSE*+]
   Player 4: But who would administer the fine? [*RULE INFO*] Everyone? [*PROPOSAL*]
   Player 1: Yeah [*CHOOSE*+], itd hurt more and you cant retaliate against 3 people as easily [*SOC INFO*]
   Player 2: so leaving empty spaces with 2 tokens around and fines if someone if farming for tokens [*RULE INFO*]
   ….
   Player 1: yeah [*CHOOSE*+]
   Player 3: alright [*CHOOSE*+]

**Conceptual Understanding of Enforcement (CUE)**

Researchers may wish to assess the group's collective (overall) conceptual understanding of enforcement, especially their conceptualization of using economic sanctions. The following scoring system allows you to determine the extent to which group members collectively recognize fundamental social and economic rationales, and dis/advantages, for using formal sanctioning systems to encourage compliance.

The scoring system increases in level of understanding, beginning with "0" (having no discernable rationale/understanding) to "3" (understanding and reconciling; i.e., balancing) the major pros and cons of formal sanctions. Higher scores indicate greater (more sophisticated) conceptual understanding. These scores are inspired empirically by prominent case studies (e.g., Ostrom, 1990) and experiments (e.g., Ostrom et al., 1992; Yamagishi, 1968), as well as theoretical discussion (e.g., Becker, 1974; Hardin, 1968; Hobbes 1651; Ostrom, 1998).

(0) **None.** Did not discuss enforcement (use of monetary penalties) or discussed it but could not (or did not) figure out a rationale/purpose for its use.

- Example: P1: why would we want to use monetary penalties? P2: I have no idea.

(1) **Too Costly/Harmful.** Conceptualize enforcement (monetary penalties) as too costly, harming oneself, and/or harming everyone if used.

- Example 1: P1: Monetary penalties are stupid. We should not use them, because it only hurts us. P2: yeah for real.
- Example 2: P4: Penalties cost too much. Why would we want to use them? P3: were all broke college kids.

(2) **Useful Deterrent.** Conceptualize enforcement (monetary penalties) as useful for correcting or preventing (deterring) rule/agreement violations.

- Example 1: P1: I don't really like them unless we are trying to keep each other from causing the tokens not to regenerate. P2: same
- Example 2: P1: No penalties unless someone is being greedy. P3: okay.
- Example 3: P3: We should penalize anyone who enters our corner (i.e., breaks the private property agreement). P1: good idea

(3) **Efficient Deterrent and/or Credible Threat.** Recognize or act upon the assumption that monetary penalties are more beneficial/effective (i.e., more potent) and/or efficient (i.e., less costly to any single individual) when they are coordinated.

- Example 2: P4: But who would administer the fine? Everyone?" P1: Yeah, itd hurt more And you cant retaliate against 3 people as easily.

## REFERENCES


Barnes, G.C., Hyatt, J.M., Angel, C.M., Strang, H. and Sherman, L.W. (2015). Are restorative justice conferences more fair than criminal courts? Comparing levels of observed procedural justice in the reintegrative shaming experiments (RISE). *Criminal Justice Policy Review*, *26*(2), 103-130.

Bowles, S. (2008). Policies designed for self-interested citizens may undermine “the moral sentiments”: evidence from economic experiments. *Science*, 320, 1605–1609

Brauner, E. (2018). Coding interaction: a technical introduction. Pages 165-190 *in* E. Brauner, M. Boos, and M. Kolke (Eds), *The Cambridge Book of Group Interaction Analysis.* Cambridge University Press, USA.

Colquitt, J. A. (2001). On the dimensionality of organizational justice: a construct validation of a measure. *Journal of Applied Psychology, 86*(3), 386–400.

Coupland, J., 2003. Small talk: Social functions. *Research on Language and Social Interaction*, *36*(1), pp.1-6.

De Boni, M., Richardson, A. and Hurling, R., (2008). Humour, Relationship Maintenance and Personality Matching in automated dialogue: A controlled study. *Interacting with Computers*, *20*(3), 342-353.

De Cremer, D., & Tyler, T. R. (2005). Managing group behavior: the interplay between procedural justice, sense of self, and cooperation. *Advances in Experimental Social Psychology, 37,* 151–218.

DeCaro, D. A. (2019). Humanistic rational choice: understanding the fundamental motivations that drive self-organization and cooperation in commons dilemmas. (Pages 117-131). In B. Hudson., J. Rosenbloom, & D. Cole (Eds), *Routledge Handbook of the Study of the Commons.* Routledge, New York, USA.

DeCaro, D. A. (2011). Considering a broader view of power, participation, and social justice in the Ostrom Institutional Analysis Framework. *Grassroots Economic Organizing Newsletter*, 2(9). http://geo.coop/node/651

DeCaro, D.A., DeCaro, M.S., Hotaling, J., & Johnson, J. G. (2020). Procedural and economic utilities in consequentialist choice: trading freedom of choice to minimize financial losses. *Judgment and Decision Making, 15*(4), 517-533. http://journal.sjdm.org/12/12425/jdm12425.pdf

DeCaro, D. A., Janssen, M. A., & Lee, A. (with Ostrom, E). (2015). Synergistic effects of voting and enforcement on internalized motivation to cooperate in a resource dilemma. *Judgment and Decision Making*, 10(6), 511-537.

DeCaro, D. A., Janssen, M. A., & Lee, A. (2021). Motivational foundations of communication, voluntary cooperation, and self-governance in a common-pool resource dilemma. *Current Research in Ecological and Social Psychology*, 2:100016. https://doi.org/10.1016/j.cresp.2021.100016.

DeCaro, D. A., & Stokes, M. K. (2013). Public participation and institutional fit: a social–psychological perspective. *Ecology and Society,* 18(4), 40. http://dx.doi.org/10.5751/ES-05837-180440

Deci, E. L., and Ryan, R.M. (1987). The support of autonomy and the control of behavior. *Journal of Personality and Social Psychology*, 53(4), 1024–1037.

Deci, E. L., & Ryan, R. M. (2000). The “what” and “why” of goal pursuits: human needs and the self-determination of behavior. *Psychological Inquiry, 11,* 227–268.

Eggins, S. and Slade, D., (2005). *Analysing casual conversation*. Equinox Publishing Ltd.

Frey, B. S., Benz, M., & Stutzer, A. (2004). Introducing procedural utility. Not only what, but also how matters. *Journal of Institutional and Theoretical Economics, 160,* 377–401.

Gardner, R., Ostrom, E. and Walker, J.M. (1990). The nature of common-pool resource problems. *Rationality and society*, 2(3), 335-358.

Greene, J.A., and Azevedo, R. (2007). A theoretical review of Winne and Hadwin's model of self-regulated learning: new perspectives and directions. *Review of Educational Research*, 77(3), 334-372.

Janssen, M. A. (2010). Introducing ecological dynamics into common-pool resource experiments. *Ecology and Society, 15*(2), 7. http://www.ecologyandsociety.org/vot15/iss2/art7/

Johnstone, G., and Van Ness, D. W. (2013). *Handbook of Restorative Justice.* Willan Publishing

Lerman, R.I. and Yitzhaki, S. (1984). A note on the calculation and interpretation of the Gini index. *Economics Letters*, *15*(3-4), 363-368.

Ostrom, E. (1990). *Governing the Commons: Evolution of Institutions for Collective Action.* Cambridge, UK: Cambridge University Press.

Ostrom, E., Walker, J., & Gardner, R. (1992). Covenants with and without a sword: self-governance is possible. *American Political Science Review, 86*(2), 404–417.

Ostrom, V., (1980). Hobbes, covenant, and constitution. *Publius*, 10(4), 83-100.

Ostrom, V. (1994). *The Meaning of American Federalism. Constituting a Self-Governing Society*. San Francisco: ICS Press.

Pavitt, C. (2011). Communication, performance, and perceptions in experimental simulations of resource dilemmas. *Small Group Research*, 42(3), 283-308.

Ratajczyk, E., Brady, U., Baggio, J.A., Barnett, A.J., Perez-Ibara, I., Rollins, N., Rubiños, C., Shin, H.C., Yu, D.J., Aggarwal, R. and Anderies, J.M. (2016). Challenges and opportunities in coding the commons: Problems, procedures, and potential solutions in large-N comparative case studies. *International Journal of the Commons*, *10*(2), 440-466.

Reed, N., Metzger, Y., Kolbe, M., Zobel, S., and Boos, M. (2018). Unitizing verbal interaction data for coding. (pp. 208-226) In E. Brauner, M. Boos, an M. Kolbe (Eds), The Cambridge Handbook of Group Interaction Analysis. Cambridge University Press, USA.

Rogat, T.K. and Adams-Wiggins, K.R. 2014. Other-regulation in collaborative groups: implications for regulation quality. *Instructional Science*, 42(6), 879-904.

Ryan, R.M, & Deci, E.L. (2017). Economic and political systems. In R.M. Ryan & E.L. Deci (Eds.), *Self-Determination Theory: Basic psychological needs in motivation, development, and wellness* (pp. 591-615). New York, NY: The Guilford Press.

Shank, D.B., Kashima, Y., Peters, K., Li, Y., Robins, G. and Kirley, M., 2019. Norm talk and human cooperation: Can we talk ourselves into cooperation? *Journal of Personality and Social Psychology*, *117*(1), 99-123.

Tschan, F., Zimmerman, J., Semmer, N. K. (2018). Rules for coding scheme development. Pages 191-2017 *in* E. Brauner, M. Boos, and M. Kolke (Eds), *The Cambridge Book of Group Interaction Analysis.* Cambridge University Press, USA.

Tyler, T. R. (1990). *Why people obey the law.* New Haven, CT: Yale.

Tyler, T.R. (2006a). Restorative justice and procedural justice: dealing with rule breaking. *Journal of Social Issues*, *62*(2), 307-326.

Tyler, T. R. (2006b). Psychological perspectives on legitimacy and legitimization. *Annual Reviews of Psychology, 57,* 375–400.

van Prooijen, J. W. (2009). Procedural justice as autonomy regulation. *Journal of Personality and Social Psychology*, *96*(6), 1166–1180.

Vollan, B. (2008). Socio-ecological explanations for crowding out effects from economic field experiments in southern Africa. *Ecological Economics, 67,* 560–573.

Wittenbaum, G.M., Hubbell, A.P. and Zuckerman, C. (1999). Mutual enhancement: Toward an understanding of the collective preference for shared information. *Journal of Personality and Social Psychology*, *77*(5), 967-978.

Yu, D.J., Shin, H.C., Pérez, I., Anderies, J.M. and Janssen, M.A. (2016). Learning for resilience-based management: Generating hypotheses from a behavioral study. *Global environmental change*, *37*, 69-78.
